# Stable electron-irradiated [1-^13^C]alanine radicals for metabolic imaging with dynamic nuclear polarization

**DOI:** 10.1126/sciadv.adz4334

**Published:** 2025-11-21

**Authors:** Catriona H. E. Rooney, Justin Y. C. Lau, Esben S. S. Hansen, Nichlas Vous Christensen, Duy A. Dang, Kristoffer Petersson, Iain D. C. Tullis, Borivoj Vojnovic, Sean Smart, William Myers, Zoe Richardson, Jarrod Lewis, Brett W. C. Kennedy, Alice M. Bowen, Lotte Bonde Bertelsen, Christoffer Laustsen, Damian J. Tyler, Jack J. Miller

**Affiliations:** ^1^ Department of Physiology, Anatomy and Genetics, University of Oxford, UK.; ^2^ GE HealthCare, Waukesha, WI, USA.; ^3^ The MR Research Centre, Aarhus University, Aarhus, Denmark.; ^4^ Department of Oncology, University of Oxford, UK.; ^5^ Centre for Advanced ESR, Department of Chemistry, University of Oxford, UK.; ^6^ Department of Physics, University of Oxford, UK.; ^7^ Department of Material Science, University of Oxford, UK.; ^8^ The National Research Facility for Electron Paramagnetic Resonance, Photon Science Institute and Department of Chemistry, University of Manchester, UK.; ^9^ OCMR, Cardiovascular Medicine, University of Oxford, UK.

## Abstract

Dissolution dynamic nuclear polarization (dDNP) increases the sensitivity of magnetic resonance experiments by >10^4^-fold, permitting isotopically labeled molecules to be transiently visible in magnetic resonance imaging scans. dDNP mechanistically takes place at ~1 K and requires unpaired electrons and microwaves. These electrons are usually chemical radicals, requiring removal by filtration prior to injection into humans. Alternative sources, such as ultraviolet irradiation, generate lower polarization and require cryogenic transport. We present ultrahigh–dose rate electron irradiation as an alternative for generating nonpersistent radicals in alanine/glycerol mixtures. These are stable for months at room temperature, quench spontaneously upon dissolution, are present in dose-dependent concentrations, and generate comparable nuclear polarization (17%) to trityl radicals used clinically (19%) through a previously unknown mechanism we believe to involve partial ordering and electron-electron interactions. Owing to the large radiation doses required, this process is sterilizing, permits imaging of alanine metabolism in vivo in the rat kidney, and may aid clinically translating dDNP.

## Teaser

Six-MeV electrons generate stable radicals for more accessible metabolic imaging with dDNP through a previously unknown mechanism.

## INTRODUCTION

Hyperpolarized magnetic resonance imaging (HP MRI) is a molecular imaging technique that is predominantly used to monitor metabolic fluxes in real time by transiently making a labeled molecular probe visible to magnetic resonance (MR) experiments such as MRI. Over the past two decades, its preclinical use has spanned multiple fields of basic scientific and medical research, with 2013 marking the start of its validation for various clinical applications, including prostate cancer (*1*, *2*–*14*), traumatic brain injury (*15*, *16*–*18*), heart failure (*19*–*26*), and ischemic heart disease (*27*–*30*), among many others (*31*). To perform HP MRI, a hyperpolarizer is required, which is a device that transiently enhances the MR signal of stable labeled isotopes by several orders of magnitude. One method to achieve this is through dissolution dynamic nuclear polarization (dDNP), which uses cryogenic temperatures, high magnetic-field strengths (>3 T), and microwave irradiation. A typical sample for dDNP comprises a metabolic substrate mixed with a chemical radical species and optionally also a glassing agent such as glycerol. At low (~1 K) temperature, the unpaired electron spins present in the radical become thermally polarized. When irradiated with microwaves, a population inversion of these electronic spins can be created, and they are driven to relax, primarily through dipolar interactions, and ultimately transfer their polarization to nearby nuclei. The net process behind DNP therefore is that nuclear polarization above that at thermal equilibrium is created. Once the desired nuclear signal enhancement is achieved, a heated and pressurized solvent is introduced to rapidly melt the solid-state sample and convert it into a form suitable for injection into a living system. Following injection, the enzymatic conversion of the hyperpolarized substrate into other metabolic intermediates can be temporally and spatially monitored in vivo through MR experiments such as MRI.

Hyperpolarized pyruvate has become the most widely used substrate in HP MRI due to its molecular utility and importance in biochemistry and the high degree of polarization (>50%) that can be obtained with dDNP (*32*). However, in clinical settings, its preparation is complicated by the chemical radicals used, which are considered investigational molecular agents and cannot be injected in high concentrations into humans. This requires either (i) chemical engineering to produce a soluble trityl radical that can be filtered out or (ii) photon irradiation to generate transient radicals that serve as the electron source necessary for dDNP and quench rapidly upon dissolution (*33*). Approach (i) has been undertaken by all clinical trials to date, developing a trityl radical, OX063-Me, also known as electron paramagnetic agent (EPA) or AH111501, which precipitates under the acidic aqueous conditions of dissolution and is mechanically filtered. This avoids regulatory concerns, as the median lethal dose (LD_50_) of trityl radicals is ~8 mmol/kg (*34*) and they are present in samples at concentrations above this. The maximum permissible radical concentration is agreed with regulators worldwide, typically <3 mM, and many sites routinely achieve <5 μM (*31*). The key advantage of approach (ii) is the elimination of the filtration step, enabling faster and more efficient use of the sample and the extended duration of the enhanced MR signal (i.e., a lengthened *T*_1_ relaxation time), as it avoids the presence of unpaired electrons that act as relaxation centers following DNP (*35*). To date, both ultraviolet (UV) (*36*) and gamma irradiation (*37*) have shown potential.

However, regardless of whether the electron source is chemical or photon based, there are substantial technical limitations on the ability of clinical sites to ship and receive compounds for hyperpolarization with the radicals in situ. For instance, the chemical instability of the trityl radical, the most commonly used chemical radical, renders centralized production of prefilled fluid paths for global distribution infeasible; fluid paths are transported typically at 195 or 253 K (*31*, *38*, *39*). In contrast, while low-energy gamma-generated radicals are reported to be stable at room temperature for several months to produce a single sample, irradiation can take days to months, and reported nuclear enhancement factors are on the order of 10^2^ and not the 10^5^ obtained with chemical radicals (*40*). DNP with UV-generated radicals is only possible if samples are optically transparent (e.g., pyruvic acid) and require low-temperature (cryogenic) transportation (*33*), which incurs a substantial expense and is not routinely performed, as precise control of magnetic field *B* and temperature *T* is required over many orders of magnitude (*41*). Ultimately, sites conducting human dDNP experiments generally require local access to a pharmacy equipped with sterile compounding facilities to comply with the high degree of sterility assurance required. This is complex from a regulatory view (*31*) and is considered a substantial financial burden that may ultimately limit the accessibility of the technique.

Here, we present an alternative approach to generating sterilized samples for clinical HP MRI that are suitable for convenient transport and centralized manufacture, potentially offering an alternative for sites that do not have access to a pharmacy. Using an ultrahigh–dose rate 6-MeV electron linear accelerator (linac), we demonstrate the creation of endogenous radicals within polycrystalline alanine, the concentration of which can be easily tuned by adjusting properties of the electron beam (e-beam). Alanine is commonly used for radiation dosimetry, and the radicals generated upon irradiation are known to be stable for many years at room temperature if stored anhydrously (*42*, *43*). To improve thermal conductivity and permit efficient spin diffusion between polarizing centers, irradiated polycrystalline alanine was dispersed in glycerol, resulting in radical lifetimes that were stable for several weeks when stored at room temperature with silica desiccant gel. Upon dissolution, radicals were quenched, yielding a nontoxic (*44*, *45*) directly injectable substrate. At 6.7 T, we were able to obtain nuclear polarization levels of ~20% and found that the polarization transfer is not simply explained by the solid effect or models of thermal mixing with the known stable alanine radical (SAR) *g*-factor and hyperfine couplings at this field strength. This is unlike similar analytic models of DNP with pyruvate and disperses chemical free radicals such as trityl (*46*). Crucially, the hyperpolarized ^13^C label on alanine was successfully transferred to pyruvate and lactate after injection in vivo, demonstrating the potential of this method for metabolic imaging.

## RESULTS

### Irradiation of samples

**Fig. 1.**
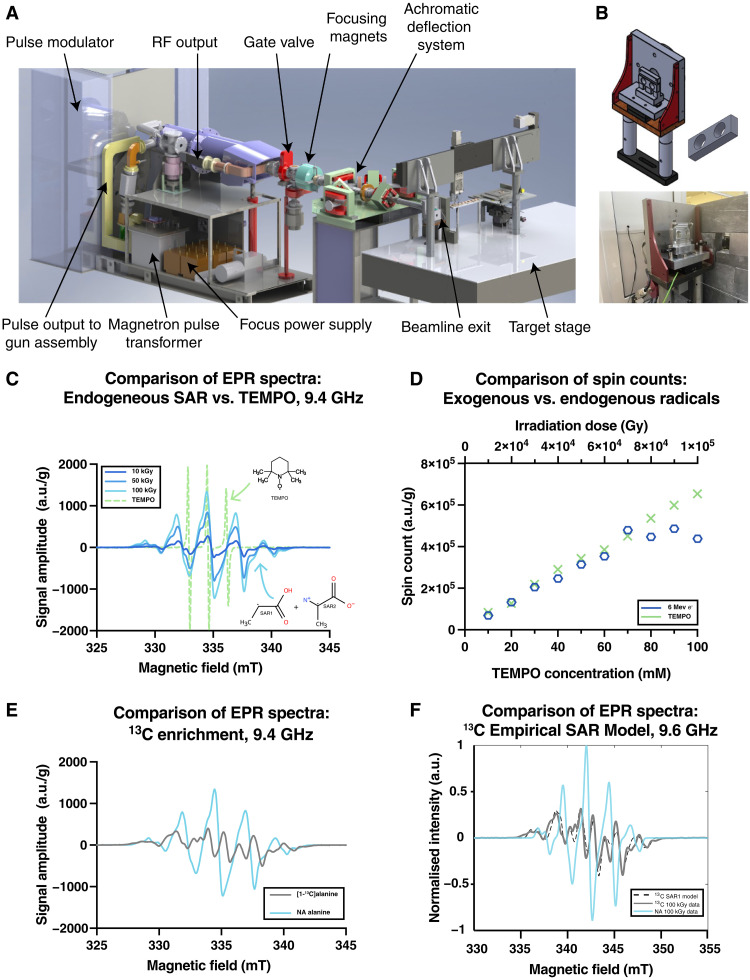
Radical generation and quantification. (**A**) An ultrahigh dose–rate optimized in-house–developed linac was used to bombard (**B**) a target containing polycrystalline [1-^13^C]alanine with 6-MeV electrons. (**C**) These generate SARs detectable by EPR in a dose-dependent fashion compared to (**D**) TEMPO as a concentration standard. (**E**) These EPR data were acquired in a single continuous wave (CW) EPR scan at 9.38611 GHz with a modulation frequency of 100 kHz, and sweep rate of 0.816 mT/s. The ^13^C-hyperfine interaction in the SARs, significantly he EPR spectrum compared to NA alanine, which we modeled (**F**) via simulations in EasySpin, is shown frequency-corrected to a standard frequency of 9.6 GHz, with our ^13^C-model in black (detailed in text S6.8). a.u., arbitrary units.

Dry polycrystalline natural-abundance (NA) and [1-^13^C]l-alanine powders were irradiated using a FLASH-optimized (*47*) in-house–developed 6-MeV (nominal energy) electron linac (*48*). This specialized accelerator (Fig. 1A) was based on a reconfigured Elekta SL75/5 traveling-wave waveguide with an S-band radio frequency (rf) magnetron source (2.89 GHz) and delivers ultrahigh–dose rate horizontal 6-MeV e-beam 3.5-μs macropulses, with adjustable pulse repetition rates (25 to 300 Hz) through precise control of rf and injection parameters (*48*). Unlike conventional medical linacs that use 90° bending magnets, our system delivered electrons in a straight-through beam path with specialized quadrupole focusing and advanced beam monitoring systems (*48*, *49*), allowing exquisite control of dose per pulse across multiple orders of magnitude, and was uniquely able to irradiate alanine samples, provided as a powder sample (Fig. 1B), with up to 100 kilogray (kGy) in seconds. Further details are provided in text S6.1.

We undertook electron paramagnetic resonance (EPR) spectroscopy to quantify radical species generated by electron irradiation. As radiation damage in dry, NA alanine is known to produce SARs that have been extensively studied, both via EPR and theoretically (*50*–*57*), we compared experimental EPR spectra to that of TEMPO [(2,2,6,6-tetramethylpiperidin-1-yl)oxyl], an aminoxyl radical used for DNP, and additionally quantitatively verified their dose/response curve. Endogenous alanine radicals had a broader linewidth than TEMPO (Fig. 1C), and, as expected, an increase in the irradiation dose led to a greater spin count, saturating at ~70 kGy (Fig. 1D) for the irradiated sample. We found that [1-^13^C]alanine had a drastically different EPR spectra compared to NA alanine, reflecting a hyperfine interaction to the ^13^C atom (not altering the corresponding spin count; text S6.7). The *g*-tensor and proton/nitrogen hyperfine interaction constants *A* for NA alanine have previously been well documented (*54*, *55*, *57*, *58*). However, no such anisotropic terms for [1-^13^C]alanine have been measured experimentally, although they have been predicted by density functional theory (DFT) calculations (*59*). The EPR spectra obtained for both the NA and labeled samples at room temperature can be modeled reasonably using published hyperfine coupling values and a weighted combination of two SARs (see text S6.7). For the purposes of predicting DNP behavior, we considered a single dominant species and modeled our system as a powder containing the labeled predominant SAR species, known as R1 (^13^C-SAR1). We determined, using a constrained Bayesian optimization routine (*60*), the values of the hyperfine coupling tensor to the ^13^C, A_13C_, and the *g*-tensor (Fig. 1F and text S6.8); the principal values determined for A_13C_ were [−1.80266, −15.8427, 64.6933] MHz with Euler angles of [1.50914, 1.30749, 0.761318] rad in the previously reported molecular frame (*54*), and these are of a similar order of magnitude to the previously reported theoretical values calculated via DFT (*59*), although the accuracy of the A_13C_ hyperfine values obtained is limited by the resolution of the linewidth of the EPR spectrum obtained. We additionally measured the electronic *T*_1*e*_ and apparent *T*_2_, *T*_m_ at 5 K, and found that 100-kGy irradiated alanine had a long *T*_1_ (7.6 s) in comparison to OX063 (1.4 s) but similar *T*_m_ (332 versus 370 ns; see text S6.7).

### DNP via irradiation-induced radicals

**Fig. 2.**
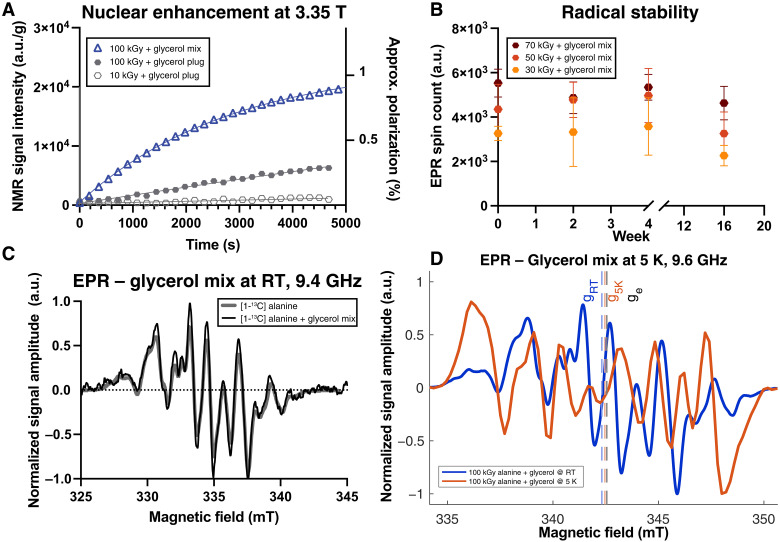
DNP and glycerol addition. (**A**) DNP buildup profiles of ^13^C nuclear magnetic resonance (NMR) signal arising from different high–dose rate irradiated samples at 3.35 T; we found that dispersing irradiated polycrystalline alanine powder in glycerol produced substantially higher polarization. (**B**) Longitudinal measurements of the spin counts showed that there was no significant effect on the endogenous radical concentration with increased duration of storage in anhydrous glycerol up to 4 months, in sharp contrast to pyruvic acid/trityl radical mixtures, which quench within hours (mean ± SD of three technical replicates). (**C**) Alanine/glycerol mixtures did not significantly alter acquired EPR data, but at (**D**) 5 K, the electronic environment altered considerably. These data were obtained via either CW EPR as previously [room temperature (RT)] at 9.38611 GHz or at 5 K on a pulsed instrument at 9.76344 GHz and have been frequency-corrected to 9.6 GHz in (D) or 9.4 GHz in (C), for display on a common scale; see text S6.7.

We first tested the feasibility of using the irradiation-induced radicals for DNP by exploring their polarization characteristics and stability during sample storage. We investigated both dry powder mechanically secured in place with a separate snap-frozen “plug” of anhydrous glycerol and an alanine and glycerol mixture. This plug prevented the dry alanine powder from being displaced by any turbulent gas flow present and additionally matched the dielectric loading conditions of the alanine/glycerol mixture, preserving rf coil loading and microwave power delivery between the two conditions. We further explored the comparatively limited effect of altering microwave power; see text S6.3. We found that the obtained nuclear polarization increased with increasing radical concentration and that substantially greater nuclear enhancement at 3.35 T was obtained with alanine crystallites dispersed in the glycerol matrix (Fig. 2A). These mixtures were stable for weeks independent of radiation dose, as quantified by EPR (Fig. 2B), with no significant change in concentration versus time (see text S6.4). The addition of glycerol did not change the electronic environment at room temperature as seen by EPR (Fig. 2C), but did at 5 K (Fig. 2D) with substantially altered hyperfine interaction constants and a shift in the mean isotropic electronic *g*-value (from *g* = 2.003698 to *g* = 2.002862; shown integrated in fig. S11) that we were not able to model. The shape of the EPR spectrum at 5 K is likely indicative of a larger hyperfine interaction to the ^13^C atom, similar to that previously observed for the primary radical species, which is only stable at low temperature (*61*), indicating a further distribution over interconverting molecular species (see text S6.8). A frequency sweep at 3.35 T (fig. S2) was difficult to interpret, with a small lobe of nuclear enhancement potentially present far away from the central maximum.

**Fig. 3.**
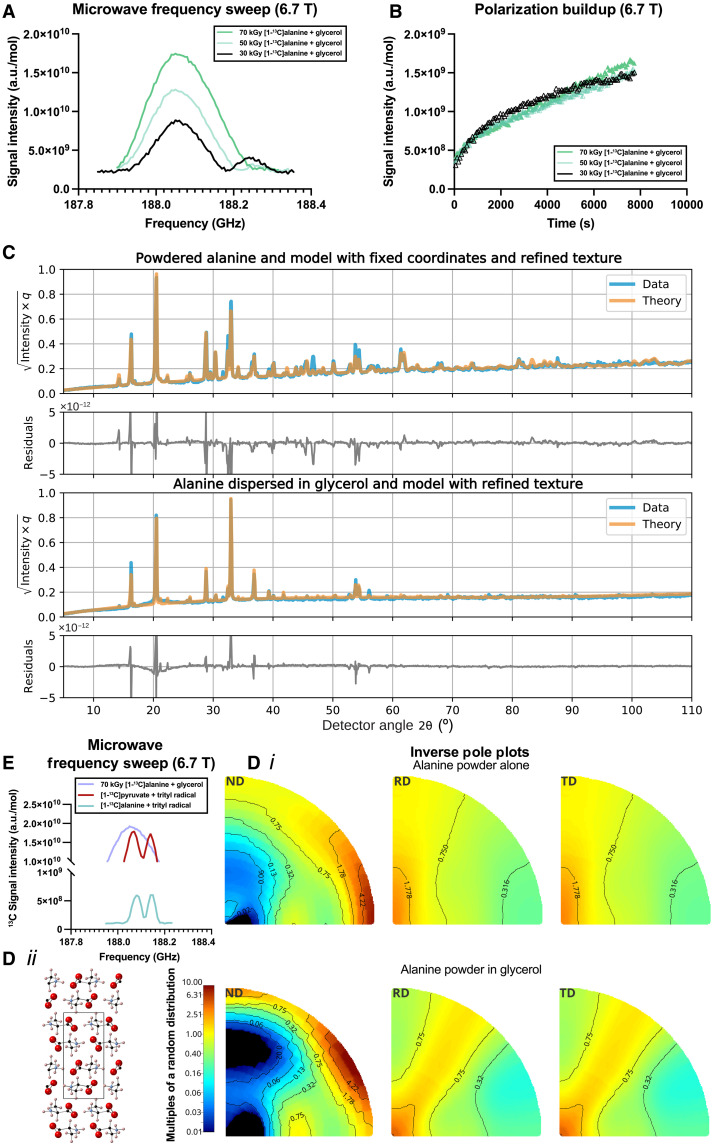
DNP at 6.7 T and partial order. (**A**) The frequency sweep profile of DNP with alanine/glycerol mixtures showed qualitative differences with increasing radiation dose, which we hypothesize is due to cooperativity. (**B**) Polarization buildup as a function of time showed profound biexponential behavior under these conditions, distinct from narrow linewidth radicals such as trityl, all arising from ^13^C NMR on [1-^13^C]alanine. (**C** and **D***i*) X-ray diffractometery measurements of both samples were able to demonstrate that polycrystalline alanine remained dispersed (not dissolved) in glycerol and was well described by (**D***ii*) existing crystal structures with slight refinement but had profound crystallographic texture, indicative of an ordered crystalline arrangement within the glycerol matrix. (**E**) The efficiency of DNP under these conditions is unexpected, comparable to narrow linewidth trityl radicals. Further experimental details are provided in the Supplementary Materials; as is conventional with texture analysis the inverse pole plot D*i* is presented along the normal, rolling and transverse directions, ND, RD, and TD.

Given the comparatively wide EPR linewidth of alanine radicals at 3.35 T, we elected to undertake DNP at 6.7 T. Under these conditions, the relatively small degree of *g* anisotropy would be expected to scale proportional to *B*_0_, whereas the strong hyperfine couplings are field independent (depending primarily on the Fermi contact mechanism). We therefore expected to a first approximation an improvement in DNP efficiency through an analytic model of the thermal mixing process of polarization growth based on the Provotorov equations (*62*), as the effective width of the EPR line shape *D* would be expected to decrease, and the resolvable resolution of the DNP frequency sweep serve would, we believe, be predicted to increase at higher field. We additionally explored radiation doses (30, 50, and 70 kGy) to reflect the concentrations of broad-linewidth exogenous chemical radical species typically used for DNP (i.e., 30, 50, and 70 mM). We did not observe the characteristic “bimodal” frequency sweep pattern associated with either the well-resolved solid effect or thermal mixing mechanisms, the latter of which is expected to describe DNP with nitroxide (*63*) or trityl (*64*) radicals and produces maxima approximately at the sum or difference of the nuclear and electron Larmor frequencies ( $\omega_{e}\pm \omega_{N}$ ). Instead, we clearly observed an asymmetric “hump” at higher frequency to the major lobe of the curve that decreased with increasing radiation dose (i.e., approximately at $\omega_{e}$ alone) as illustrated in Fig. 3A. At lower doses (30 kGy), we observe a bimodal microwave profile with a prominent second lobe that progressively diminishes as the radiation dose increases to 50 kGy and ultimately disappears entirely at 70 kGy. This systematic change suggests a transition in the underlying polarization transfer mechanism that correlates directly with radical concentration. A biexponential buildup profile was also observed (Fig. 3B) following irradiation at the maximum microwave frequency, with a fast component decreasing from 22.9 to 14.9 s and a slow component increasing from 0.92 to 6.88 hours as the radiation dose increased from 30 to 70 kGy (see table S2). This trend correlates with the disappearance of the bimodal frequency profile: Samples exhibiting pronounced bimodal behavior (30 kGy) show faster overall polarization kinetics, whereas those with unimodal profiles (70 kGy) exhibited slower kinetics. Although some degree of biexponential behavior is expected for dilute electronic sources owing to spin diffusion (*65*, *66*), the longer time constant would decrease with increasing radical concentration, which we did not observe. A summary of fitted time constant parameters is provided in Table 1.

**Table 1.** Summary of DNP buildup parameters from biexponential fits across different experimental conditions and radical sources. Buildup profiles follow the biexponential model: $y=P\left[A\left(1-e^{-t/T}\right)+\left(1-A\right)\left(1-e^{-t/\tau }\right)\right]$ , where *P* is the buildup constant, *A* is the fast component weighting, *T* is the fast component time constant, and τ is the slow component time constant. It was not possible to fit 3.35 T data to a biexponential curve stably, and, likewise, a biexponential fit to trityl data at 6.7 T returned monoexponential behavior. Arbitrary units are comparable within experiments but not between them.

Figure sample/condition		Dose (kGy)	Buildup amplitude *P* (a.u.)	Fast time *T* (s)	Slow time τ (hour)	Weighting *A*
2a	10 kGy (glycerol plug)	10	9.59 × 10^2^*^^	Monoexponential fit poor; τ_mono_ = 3171342310 s		
	100 kGy (glycerol plug)	100	6.28 × 10^3^*^^	(τ_mono_ = 19236 s)		
	100 kGy (glycerol mix)	100	2.37 × 10^4^*^^	(τ_mono_ = 2836 s)		
3b	30 kGy (glycerol mix)	30	1.52 × 10^6^	22.9	0.92	0.264
	50 kGy (glycerol mix)	50	2.28 × 10^6^	22.2	2.69	0.193
	70 kGy (glycerol mix)	70	5.11 × 10^6^	14.9	6.88	0.099
4c	^13^C-pyruvate + OX063	–	4.1 × 10^1^	(τ_mono_ = 1653 s)^†^		
	^13^C-alanine + OX063	–	2.3 × 10^1^	(τ_mono_ = 1898 s)^†^		
	70 kGy (glycerol mix)	70	1.7 × 10^1^	20	0.481	0.088

* These data are at 3.35 T.

† OX063 trityl radical shows purely monoexponential behavior (*A* = 0 in one fit; identical time constants in the other).

To understand this difference upon the addition of glycerol, we undertook x-ray diffraction (XRD). Data were obtained from NA polycrystalline alanine and polycrystalline alanine dispersed in glycerol; a spherical harmonic Rietveld crystallographic texture refinement (*67*) aiming to quantify any degree of order in the sample was undertaken with MAUD (Material Analysis Using Diffraction) (*68*). Using a neutron-diffraction crystal structure obtained on the SXD (Single Crystal Diffractometer) instrument (*69*) at the ISIS neutron source (*70*), we demonstrated strong partial ordering of the sample (Fig. 3C) that changed (and strengthened) upon addition of glycerol (Fig. 3D). As is common in texture analysis, we express orientation in units of multiples of a random distribution (mrd), where a sample without preferred orientation is one mrd homogeneously in all directions; alanine and glycerol mixtures had orientation preferences occurring at >10 mrd and are partially ordered. This was consistent with ab initio liquid-state molecular dynamic simulations performed on two semi-infinite glycerol/alanine domains using the LAMPS (*71*) with EMC (*72*) that predicted the immiscibility of the two substances (text S6.10).

This (Fig. 3A) microwave sweep profile is distinct from others previously reported, and the mechanistic processes by which DNP occurs appear as efficient as trityl radicals used in clinical research. We therefore undertook control DNP experiments comprising substrates polarized via exogenous chemical radical trityl species, namely, ^13^C-pyruvate with 30 mM AH111501 and ^13^C-alanine with 38 mM OX063 trityl (Fig. 3E). We observed that the greater the radical concentration, the longer the slow component of the buildup time and the shorter the fast component (text S6.5).

**Fig. 4.**
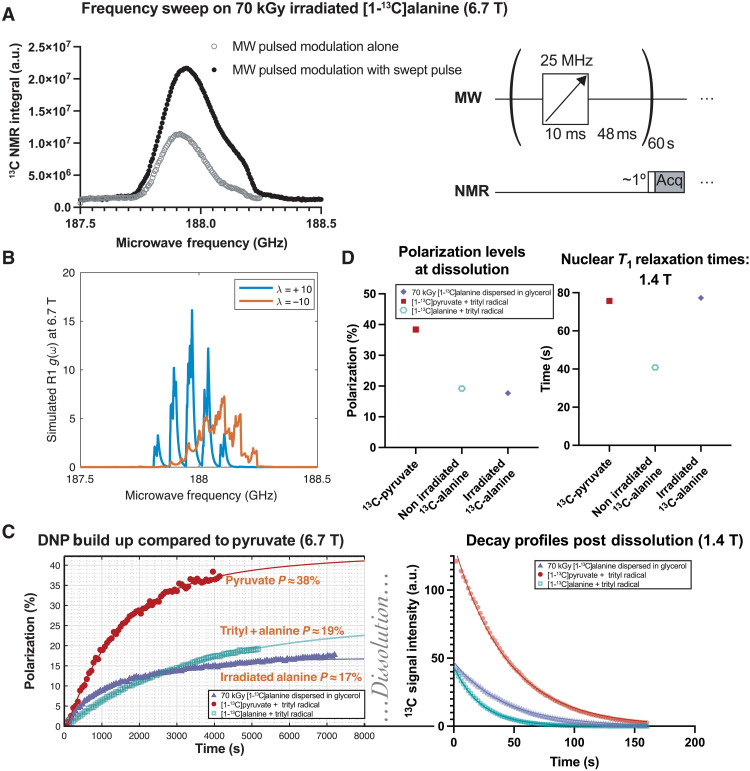
Polarization optimization and high-field comparisons with trityl. (**A**) At 6.7 T, a unimodal, always-positive frequency sweep curve was observed. Pulsed modulation with a swept-frequency microwave (MW) pulse as illustrated obtained a significant increase in nuclear polarization. (**B**) The molecular orientation of the alanine radical matters significantly for the spectral density function g(ω) as defined by Wenckebach (*76*) simulated here using from powder ^13^C alanine EPR data and assuming a partial ordering parameter λ. (**C**) The resultant limiting polarization obtained was high and comparable to the use of trityl radicals with alanine, with biexponential fit shown. (**D**) After dissolution, spontaneous quenching of endogenous radicals formed by electron irradiation resulted in a lengthened *T*_1_ (77 s) compared to that with trityl (40 s) at 1.4 T [fits shown in (C)]. Further experimental details are provided in the Supplementary Materials.

Given the wide EPR line shape of the SAR, we investigated the use of a frequency-swept microwave pulse sequence for DNP. This resulted in an increased polarization transfer and increased enhancement, within a factor of 2.5 of the “gold standard” pyruvate with trityl radical (Fig. 4A). In an attempt to understand the mechanism behind this observation, we simulated the fitted EPR line shape under DNP conditions by modeling our room temperature data as containing ^13^C-SAR1. We found that a polycrystalline distribution of R1 radicals have distinct spectral density functions g(ω) depending on preferred orientation, quantified by the order parameter λ where the angle α between the molecular *z* axis and *B*_0_ is distributed by $p\left(\alpha \right)=\text{exp}\left[-\lambda \left(3{\text{cos}}^{2}\alpha -1\right)/2\right]$ , with the orientation preference found by XRD corresponding to a combination of these parameters (Fig. 4B), whose sum could approximately trace out the enhancement curve obtained. A detailed quantum-mechanical simulation of this pulse sequence together with the known radical parameters was then undertaken in the advanced MR simulation environment Spinach (*73*). Despite including more than 50 ^13^C spins representing an alanine supercell with detailed measurements of its proton and carbon chemical shift tensors (*58*, *74*), simulating the entirety of the minute-long experiment at high temporal resolution (compared to the electron Larmor frequency) and using the Arcus-C supercomputing cluster (*75*), we were unable to reproduce this experimental result and instead predicted a bimodal curve qualitatively similar to that expected for thermal mixing (text S6.11). Previously published analytic models of DNP via thermal mixing and the cross effect (*62*, *76*, *77*) likewise produce a predicted bimodal curve with either predicted analytic (from our model) or experimental EPR data (text S6.12). A pseudopotential plane-wave simulation in Quantum ESPRESSO (*78*) with our structures predicts that alanine crystals are a direct-gap semiconductor with a large (5 eV) bandgap [text S6.13; experimentally validated in (*79*)]; were the effect of electron irradiation sufficient to dope spins into the conduction band, we would expect to see a well-resolved solid effect mechanism. This, again, did not match the data. We hypothesize that the density of unpaired electrons is insufficient to form a delocalized conduction band but rather creates localized midgap states. This intermediate regime, where radicals are neither fully isolated molecules [as often assumed in molecular DFT calculations used for alanine radicals; (*54*)] nor fully delocalized carriers [as in classical solid effect DNP systems such as paramagnetic salts; (*80*)], may explain why neither approach fully captures our experimental observations. Another possibility is the competition of multiple, competing radical species that interact and interconvert, providing a superposition of radical environments. Further work is required to assess exactly the degree remaining of molecular character in these complex, diffuse, partially ordered systems, which experience significant inter-radical coupling mediated by the alanine crystal lattice and glycerol matrix, necessitating models that bridge molecular and solid-state treatments that we were unable to provide.

Nevertheless, encouraged by the feasibility of polarizing [1-^13^C]alanine via irradiation-induced radicals, our focus turned to exploiting this for biomedical imaging. Empirically optimized DNP schemes compatible with our hardware produced an estimated solid state polarization of ~20% (Fig. 4C), similar to that obtained with a separate sample of [1-^13^C]alanine and OX063 trityl radical, both of which are comparable to the “gold standard” used clinically of pyruvate with the trityl radical AH111501, which polarized to ~40% (Fig. 4C). After dissolution, the immediate quenching in aqueous media of endogenous alanine radicals (*81*) resulted in an increase in the apparent liquid-state *T*_1_ at 1.4 T (Fig. 4D), as may be expected from the removal of paramagnetic relaxation centers (*36*).

### In vivo application as a metabolic tracer

**Fig. 5.**
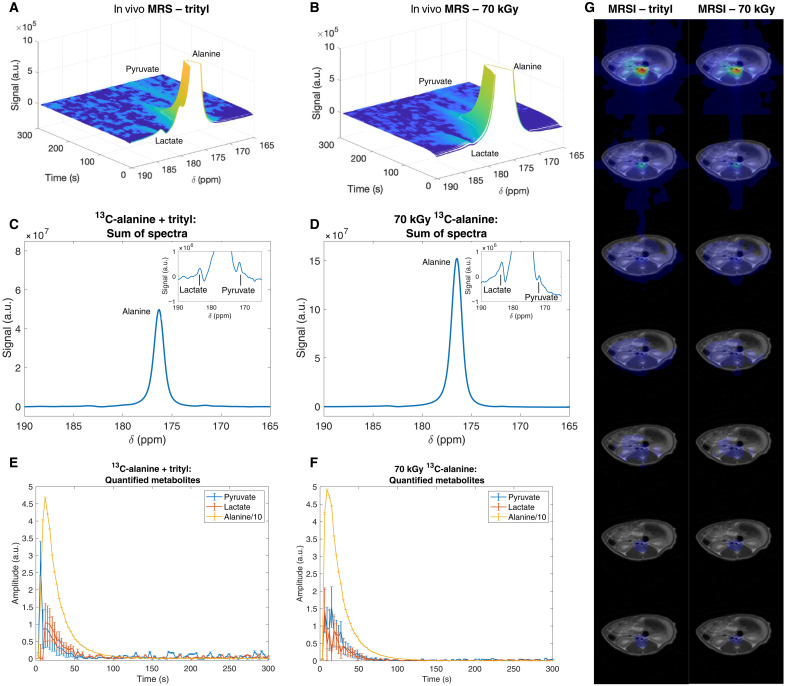
In vivo demonstration in the rat. (**A** and **B**) Stacked and (**C** and **D**) summed spectra from slice-selective spectroscopic acquisitions following separate tail-vein injections of [1-^13^C]alanine polarized via 38 mM OX063 trityl radical and hyperpolarized irradiated ^13^C-alanine. [(C) and (D)] Inset: A magnification of the spectra presented in the panels to highlight the intensities and positions of the downstream metabolites, whose chemical shift values match those reported in the literature for ^13^C-lactate and ^13^C-pyruvate. (**E** and **F**) After quantification by AMARES, metabolite dynamics were comparable between the two methods of preparation. (**G**) MRSI images obtained in a separate rat were likewise near identical, validating the use of the technique for in vivo imaging of redox potential, alanine images of which are shown concatenated over time. Further experimental details are provided in the Supplementary Materials.

To test the optimized irradiated alanine preparation and DNP protocol, two proofs of concept in vivo studies were conducted in rats. First, slice-selective MR spectroscopy acquisitions were performed following separate tail-vein injections of [1-^13^C]alanine polarized with trityl radicals as described previously (*82*) or endogenous radicals generated via 70-kGy e-beam irradiation. Temporal spectroscopy analysis demonstrated higher initial signal magnitude from the irradiated sample relative to the trityl-based preparation (Fig. 5, A and B). Secondary resonances were observed flanking the primary [1-^13^C]alanine signal in both preparations. Spectral summation was performed to enhance the signal-to-noise ratio (SNR) and facilitate metabolite identification (Fig. 5, C and D). The chemical shifts of these secondary resonances [183 and 171 parts per million (ppm)] corresponded precisely with the literature-reported values for [1-^13^C]lactate and [1-^13^C]pyruvate, respectively (*83*–*86*). Subsequent metabolite quantification via the AMARES algorithm revealed comparable metabolic dynamics between both preparation methods (Fig. 5, E and F), and spectral SNR was sufficient to quantify the downstream metabolites expected in vivo, although the comparatively long repetition time (TR) of 3 s precluded a detailed study of downstream kinetics while preserving magnetization. No additional labeled peaks were observed; the products of the dissolution were identical to that observed with trityl radical (see text S6.14). The dissolution was “clean,” meaning that the entire volume of the sample was dissolved by the 5 ml of D_2_O used as a dissolution buffer with no remaining partially dissolved sample remaining, a final concentration of ~100 mM dissolved alanine was obtained postdissolution, and, subsequently, 1 ml was manually injected over 5 s.

Second, comparative two-dimensional (2D) MR spectroscopic imaging (MRSI) was performed in a separate animal using identical hyperpolarized [1-^13^C]alanine preparations. MRSI data were acquired, together with *T*_2_-weighted proton images acquired in the axial plane for anatomical localization. Spatiotemporal analysis of the spectroscopy data demonstrated concordant biodistribution patterns between the trityl-based and irradiation-based preparations (Fig. 5G), with predominant accumulation in the renal parenchyma as alanine perfuses the kidneys, consistent with previous renal reports and enabling the technique to quantify redox potential (*82*) and confirming equivalent in vivo behavior between preparation methods.

## DISCUSSION

The clinical feasibility and utility of metabolic imaging with HP MRI and dDNP have been consistently demonstrated since its inception 22 years ago (*87*), with the transformative ability to spatially map metabolism of huge interest in many disparate diseases including cancer (*1*), heart disease (*88*), and diabetes (*89*). With more than 1000 human patients scanned and multicenter trials soon to commence (*90*), it is a physical technique that has opened new avenues for research and the diagnosis and management of disease. However, for its integration within routine clinical workflows to be fully realized, significant improvements in its cost, accessibility, and practicality are essential. Beyond the substantial capital expenditure for dDNP hardware (typically exceeding $2 million), institutions using dDNP face prohibitive ongoing operational costs. These include maintaining dedicated clean-room pharmacy facilities with specialized personnel for the sterile preparation of per-patient disposable fluid paths for dDNP, implementing expensive quality control measures, and adhering to stringent regulatory frameworks for pharmaceutical preparation. Each hyperpolarized examination currently requires the labor-intensive preparation of sterile fluid paths (*91*), their filling with sterile stable isotope–labeled metabolites together with dissolution and neutralization media [both manufactured according to sterile guidelines that vary regionally; (*90*)] and a trityl radical that provides the unpaired electron the technique requires. The synthesis of trityl radicals is not trivial, typically requiring ~13 separate organic synthetic steps, leading to a cost >$10,000/g (*34*). Moreover, the required subsequent filtration steps to remove the radical add further complexity to the design of the fluid path. The trityl radical used in human studies, AH111501, also known as EPA, is the methylated form of OX063 and is specifically engineered to precipitate out in acidic aqueous solution for filtration within the sterile fluid path. To satisfy regulatory considerations, it is currently a release criterion for all sites performing human hyperpolarized experiments that the radical concentration is below an agreed number, typically less than 3 mM in the dissolved liquid (*90*). These are all individual processes that substantially increase per-patient costs and logistical complexity and restrict the availability of a technique that has the ability to directly measure central metabolic reactions key to all life.

Furthermore, the limited shelf-life of the radical in prepared samples [~24 hours at room temperature for trityl; (*92*)] necessitates precise coordination between pharmacy operations and clinical scheduling (*31*) and prohibits the transport of dDNP samples nationally or internationally unless extraordinary steps are taken, such as transporting samples cryogenically under defined (and varying) temperatures and magnetic fields (*41*, *93*, *94*). In addition to restraining human experiments, these large costs further constrain dDNP availability in nonhuman biological research: Although of utility in studying species such as snakes (*83*) and veterinary canine patients (*95*), trityl radicals are sufficiently expensive as to be repurified from animal urine after use (*34*).

To address these fundamental constraints on widespread adoption, we explored ultrahigh–dose rate e-beam irradiation for generating inherently biologically sterile samples with radicals in situ: A sample could be prepared in a central (worldwide) facility, rapidly sterilized, and shipped at room temperature around the world prior to use with a polarizer in a hospital site. Furthermore, the ability to use powders or crystals of neutral salts, such as alanine, rather than glassing suspensions of trityl radicals and typically organic ketoacids, further markedly ameliorates the technical requirements of the technique, as a dissolution with sterile water is easier to obtain than with a carefully measured equimolar quantity of neutralizing (basic) sterile buffer. This approach also opens new classes of molecules to dDNP, as radical formation occurs statistically throughout the sample via well-understood particle-physics interactions, rather than requiring glassed mixtures of disparate radicals (themselves being limited by exclusion from any crystals formed). This allows for effective polarization of high concentrations of virtually any anhydrous solid-state molecule that would otherwise be challenging to study. All that would be required for dissolution is a neutral sterile dissolution buffer, and, unlike UV-irradiation generated radicals in acids, there is no need for precise control of temperature and magnetic field over many orders of magnitude (*41*) for transport—in this work, we prepared samples for DNP in one country (the UK) and studied them via dDNP in another (Denmark).

As anticipated from extensive studies on NA alanine as a dosimeter, e-beam irradiation of NA alanine generated radicals in a dose-dependent manner (*96*), and a similar trend was observed from the ^13^C-enriched samples, saturating at around 70 kGy, well above the dose requirement of 25 kGy for medical sterilization under ISO 11137-2 (*97*, *98*). What is unexpected, however, is that upon mixing with glycerol, the system appears to become partially ordered, and the efficiency of DNP increases substantially. This also generates a clinically useful probe as alanine and glycerol are not miscible in the volume ratios considered here and SARs remain present for at least 16 weeks, stored anhydrously in an inexpensive plastic container with silica desiccant gel at room temperature. Furthermore, *g* changes at low temperature with this system, indicating a fundamental change in the radical electronic environment. We observed no unexpected radiation chemistry byproducts (likely because they spontaneously recombine upon the addition of hot water in the dissolution process, wherein the mixture is melted), and both glycerol and alanine are endogenous biomolecules, which are safe in vivo. This permitted the quantification in the rat kidney of downstream alanine metabolites, directly analogously to the reported biomedical uses of [1-^13^C]alanine hyperpolarized with trityl radicals (*82*, *84*, *99*–*101*).

This polarization enhancement is difficult to quantitatively understand. We have found a unique DNP mechanism that is not explained by conventional models yet produces clinically relevant levels of polarization in an extensively studied biomolecule. The alteration of the frequency sweep profile of nuclear enhancement with radiation dose we hypothesize may be due to some degree of cooperativity (interactions) between electrons when the radical concentration becomes sufficiently high that the picture of an isolated, well-defined radical species as an independent molecule breaks down. The temperature-dependent electronic *g*-shift we observed in the EPR data is consistent with magnetic ordering in our system or, at the very least, some degree of electronic reconfiguration over molecular length scales (see fig. S6), leading to a superposition of effective radical species that may interconvert and interact with neighboring molecules (alanine has four in the unit cell), including the primary electronic radical disfavored at high temperature. Spontaneous spatial ordering in neutral alanine crystallites has previously been reported to occur in strong magnetic fields (*102*), arising out of magnetic anisotropy of the l-alanine unit cell and its comparatively large (in magnitude) $\chi_{m}$ (*103*–*105*), and zirconium nitrate–doped l-alanine single crystals form magnetic order and have been used as nonlinear optical devices (*79*). While traditional paramagnetic systems maintain independent spin behavior and molecular character even at low temperatures, our partially ordered alanine crystallites dispersed in glycerol could exhibit more complex magnetic phenomena as radical centers become close enough to develop short-range magnetic correlations at low temperatures and high radiation doses within individual crystallites. The glycerol matrix in this scenario could function analogously to domain boundaries in ferromagnetic materials. By physically separating the alanine crystallites, glycerol creates discontinuities in the exchange pathways, limiting the correlation length of any magnetic ordering but permitting thermodynamically driven spin diffusion through weak coupling provided by proton/carbon spins in the matrix. This is conceptually similar to the role of Bloch walls in ferromagnetic materials, though operating at the nano- or microscale between distinct crystallite domains rather than within a continuous bulk material. This model could potentially explain several of our observations.

First, the spatial confinement of magnetic correlations to individual crystallites would naturally lead to anisotropic magnetic responses dependent on crystallite orientation, consistent with our texture analysis showing preferential alignment. Each crystallite may develop its own local magnetic environment influenced by its specific orientation relative to the external field.

Second, this partial magnetic ordering could modify the effective field experienced by nuclear spins, altering the resonance conditions for polarization transfer for that microscopic environment. In conventional DNP models, polarization transfer is mediated by electron-nuclear dipolar interactions under well-defined frequency matching conditions (e.g., $\omega_{e}\pm \omega_{N}$ for the solid effect), and the angular orientation terms in the dipolar interaction Hamiltonian ( $3{\text{cos}}^{2}\theta -1$ ) are assumed to be spatially averaged in a glass. This regionality in orientation together with a transition from individual to collective behavior would explain why the best conventional theoretical models of DNP fail to predict our observations at higher radical concentrations, as they assume only electron-nuclear interactions and/or are based on thermodynamic arguments assuming some degree of spatial homogeneity and good thermal contact. They do not account for many-body effects that emerge when multiple electrons (arising from multiple radicals in close proximity) interact collectively.

Third, the glycerol “boundaries” could create a heterogeneous distribution of magnetic environments throughout the sample. Radicals near the glycerol interface might experience different effective fields than those in crystallite interiors, leading to a distribution of resonance conditions that broadens and shifts the optimal DNP frequency. This domain-like structure might also explain the effectiveness of frequency-swept microwave pulses in our system. By sweeping across a range of frequencies, we could be sequentially addressing different subpopulations of radicals with distinct magnetic environments, each contributing to the overall polarization enhancement. The constructive summation of these contributions could explain the superior performance compared to fixed-frequency irradiation, even though the frequency swept range is small compared to the overall EPR line shape. It also provides a mechanism for the biexponential growth of polarization, which could be ascribed as being that within each crystallite occurring quickly, and that between them occurring through the dilute matrix of NA carbon-13 spins in glycerol. These results are overall unexpected, and future work will help elucidate a mechanism behind them. They represent a new paradigm in DNP, where a partial spatial ordering of spin systems creates emergent behavior not predicted by isolated electron models of molecular physics.

The levels of nuclear enhancement we achieved (*P* ≈ 17% or ϵ ≈ 142,000 at 1.4 T) compare favorably to the ϵ ≈ 700- to 800-fold enhancements reported at 11.7 T for ionizing radiation radical–induced DNP previously by cobalt-60 γ-rays by Giannoulis *et al.* (*37*) and are comparable with the optimal trityl, narrow-linewidth radicals used clinically. For a nonirradiated alanine sample prepared with trityl radical, the polarization level at the point of dissolution was 19.2% and the nuclear *T*_1_ was 40.8 s at 1.4 T. In contrast to the work by Giannoulis *et al.* (*37*), we did not observe data consistent with a simple model by the solid-effect, and obtained qualitatively different frequency-sweep curves. This may be due to differences in the radiation transport physics of ultrahigh–dose rate 6-MeV electrons versus ~1-MeV γ-rays, which will deposit more charge into the crystallites, as well as dose rate effects altering the environment around the radical center, by, for example, introducing a degree of unresolved crystallographic strain (*106*–*108*). The alanine samples of Giannoulis *et al.* (*37*) were flame-sealed and irradiated in vacuo (our samples were in air) and were also polarized as dry powders. Last, for reference, a previous study where alanine was also hyperpolarized with the trityl radical yielded a liquid-state polarization of 12.6% at the point of dissolution and an in vitro *T*_1_ of 41.5 s at 3 T (*84*). Our irradiated sample produced comparable results; polarization levels of 17.7% at the point of dissolution were achieved, while the extracted nuclear *T*_1_ relaxation time was 77.3 s at 1.4 T. The nuclear *T*_1_ relaxation time was prolonged due to the fact that the irradiation-generated radicals quench upon dissolution. Future work could explore the direct deuteration of the ^13^C-enriched alanine to potentially enhance polarization levels as previously observed for other molecules hyperpolarized by dDNP (*109*). Crucially, the pH of the irradiated alanine samples (~7) also made them suitable for in vivo trial with dissolution in water; neutral salts are readily crystallized, and substances such as sodium pyruvate likely will be readily amenable to this technique.

Beyond the physical novelty of our approach, our in vivo experiments demonstrate significant translational potential. Electron-irradiated ^13^C-alanine not only matched but, in some aspects, also exceeded the performance of conventional preparations using exogenous radicals. It is worth considering how ^13^C-alanine could compare to ^13^C-pyruvate, the most commonly used metabolic probe with dDNP and undergoing clinical trials. Although pyruvate polarizes very well with dDNP and is rapidly metabolized to lactate, alanine, and bicarbonate, the pyruvate/lactate ratio is not reflective of intracellular redox state following HP pyruvate infusion (*110*). Upon switching the injectable substrate from ^13^C-pyruvate to ^13^C-alanine, Hu *et al.* recorded more than a 10-fold increase in the ^13^C-lactate/^13^C-pyruvate ratio (*84*). The difference was suggested to be due to the uptake of ^13^C-pyruvate being limited by perfusion and downstream flux through monocarboxylate transporters during the acquisition window. An incomplete uptake of ^13^C-pyruvate would mean contributions from an extracellular ^13^C-pyruvate pool in the measured ^13^C-pyruvate signal. Furthermore, any extracellular ^13^C-pyruvate present in plasma can be converted into ^13^C-lactate by red blood cells that contain lactate dehydrogenase, leading to an “extracellular” ^13^C-lactate pool (*99*). Signals arising from intracellular and extracellular pools cannot be easily distinguished in HP MR, leading to the proposed use of ^13^C-alanine for determining the ^13^C-lactate/^13^C-pyruvate ratio. For ^13^C-alanine to be converted into ^13^C-pyruvate and, subsequently, ^13^C-lactate, it must first be exposed to the intracellular enzyme alanine transaminase. Thus, the ^13^C-pyruvate signal detected following the injection of ^13^C-alanine would be intracellular in origin. Moreover, after the metabolism of alanine is observed and quantified, the cellular redox potential can be easily determined (*99*) from the known relation $K_{\text{eq}}=\frac{\left[\text{Pyruvate}\right]\;\left[\text{NADH}\right]\left[H^{+}\right]}{\left[\text{Lactate}\right]\left[{\text{NAD}}^{+}\right]}$ where $K_{\text{eq}}=1.11\times 10^{-11}$ M. If a measure or estimate of intracellular pH is obtained, for example, from ^31^P-MRS (*111*) or through the use of hyperpolarized pyruvate (*112*), this would provide a direct mechanism by which the nicotinamide adenine dinucleotide (NAD)/NADH ratio could be noninvasively determined, as has been reported previously for trityl-hyperpolarized alanine (*99*).

Our electron irradiation approach uniquely combines this biochemical advantage with the practical benefits of in situ radical generation and spontaneous quenching, providing a powerful new tool for quantifying intracellular redox potential. It is also the first to explicitly consider partially ordered solids as substrates for biomedical dDNP rather than amorphous glasses that are commonly used with the technique [and often listed as a requirement for its success; (*113*)] Although this technique could be extended to other substrates, ^13^C-alanine represents an ideal initial candidate due to its metabolic significance, detailed quantum-mechanical study, and demonstrated biochemical and clinical relevance. This approach may reduce the technological, regulatory, and economic barriers that have constrained HP MRI primarily to specialized research centers, potentially expanding access to this powerful diagnostic capability.

### Limitations

Several limitations should be acknowledged in this work. Notably, our mechanistic understanding of the DNP process in this system remains incomplete and in need of extension via theoretical modeling. The unusual frequency sweep profiles of this highly efficient polarization process hint at cooperativity and physics beyond conventional DNP models, but a comprehensive theoretical framework that quantitatively predicts that these behaviors is lacking. Additional spectroscopy studies with variable temperature, field strength, and radical concentration would help elucidate the underlying mechanisms.

In addition, while we demonstrated stable radical formation in alanine, the applicability of this approach to other metabolically relevant molecules requires further investigation. Different molecular structures may respond differently to electron irradiation, potentially generating distinct radical species with varying stability and DNP efficiency. The specific arrangement of alanine in a crystalline lattice likely contributes to the observed radical stability and its partial ordering in glycerol (analogous to a nemantic liquid crystal), and molecules with different crystal structures, or those that do not readily crystallize, may present challenges. Furthermore, the comparatively high radiation doses considered may embrittle plastics used in the construction of fluid paths, but this phenomenon is well characterized, and many medical plastics are routinely sterilized with comparable doses (*114*, *115*). We chose to investigate alanine because it is exceptionally well studied from a radiation physics and EPR point of view and is a reported biomolecular probe investigated for probing central metabolism. Other, perhaps more commonly used, biomolecules may well be amenable to this technique, where significant further studies across all aspects of this highly interdisciplinary science remain to be elucidated. We can propose three fundamentally distinct approaches to explore this in future work: (i) by directly irradiating other molecules, (ii) by irradiating alanine powder containing microcrystallites (such as that here) and dispersing alanine radicals in the solid state with other molecules of interest, or (iii) using cocrystallized (e.g., NA) alanine and an isotopically labeled molecule of interest, under the assumption that radical species formed act as dilute spin impurities in the resulting system. We have yet to explore any of these other exciting avenues for the technique.

Last, the highest nuclear polarization levels achieved with our approach (~20%) remain lower than those reported for optimized pyruvic acid preparations with trityl radicals (>50%). While sufficient for in vivo imaging, higher polarization would improve SNR and potentially allow detection of lower-concentration metabolites. Further optimization of irradiation parameters, alanine crystallite size, and DNP conditions might narrow this gap. Likewise, owing in part to this limitation, our in vivo experiments were limited to proof-of-concept demonstrations in two healthy animals. The performance of irradiated alanine in disease models, particularly its sensitivity to pathological alterations in redox potential, requires validation in relevant preclinical models before clinical translation. The applicability of this technique may well be higher in other diseases and organs where alanine metabolism is believed to be more avid, for example, in the liver where the lactate/pyruvate ratio is closer to 10 following the infusion of alanine (rising to 20 after an ethanol challenge) (*99*).

Beyond scientific limitations, there is much that remains to be proven for the clinical adoption of this technique. At present, most European sites conducting human hyperpolarized experiments use e-beam irradiation to sterilize (25 to 45 kGy) empty “sterile fluid paths” made of polyethyletherketone (PEEK) and polyphenylsulfone (PPSU) plastics, which are filled under sterile conditions by a pharmacy team with pyruvic acid/trityl radical, snap frozen in liquid nitrogen, purged with helium under pressure, laser welded, and transported to the polarizer for ultimate human use (*31*). We envisage that filling a fluid path with reagents and irradiating the solid isotopically labeled compound to a higher level and the fluid path overall to a level for sterilization would be a feasible method to generate transportable, sealed single-use devices in a centralized “worldwide” factory with an electron accelerator. However, we have not explicitly demonstrated this, nor have we shown that PEEK or PPSU walled tubes could withstand the high radiation doses required. We likewise have not undertaken the detailed preclinical toxicity analyses to show that no byproducts or adjuncts would be formed by high-energy electron irradiation of PEEK or PPSU plastics containing solid isotopically labeled crystallite alanine, all of which is future work and necessitate completion in a validated, accredited laboratory prior to human use.

## MATERIALS AND METHODS

### Sample preparation

The samples used in this study can be categorized into three main groups: alanine containing endogenous irradiation-generated radicals, alanine mixed with the exogenous trityl radical OX063 at a concentration of 38 mM, and pyruvate with 30 mM AH1111501. These exogenous radicals differ only by an exterior methyl R-group in their chemical structure and have almost identical electronic properties; these concentrations of both trityl radicals were chosen to reflect reported optimal conditions for DNP for both molecules.

To prepare the former, dry [1-^13^C]l-alanine powder (Isotec and Cambridge Isotope Laboratories, Inc.) was irradiated using an ultrahigh–dose rate 6-MeV electron linac operating in pulsed mode, delivering 24 Gy of dose to the sample in each of the 4-μs pulses, with a pulse repetition rate of 25 Hz and an approximate beam current of 100 mA. Each pulse delivered ~480 nC of electric charge. The dose of irradiation was varied between 10 and 100 kGy depending on the desired radical concentration and was linear with the total number of pulses delivered. The dosimetry and beam delivery was controlled using a beam monitor, which has been described in detail elsewhere (*48*, *49*) and calibrated against radiochromic film (EBT-XD, Ashland Inc., Covington, KY, USA). The total time for irradiation was deliberately lengthened to avoid linac heating; ~2 g was irradiated in 160 s. The irradiated powder was then used as the main component for two different sample types: an irradiated dry powder or an irradiated powder/anhydrous glycerol mix with a 1:1 (w/w) ratio.

The alanine preparation containing OX063 trityl radical (molecular weight = 1427 g/mol; GE Healthcare) comprised 1.276 g of [1-^13^C]l-alanine mixed in 8 ml of water and 2 ml of 11.65 M HCl. This mixture was freeze dried, and ~1.5 g of alanine hydrochloride yielded. The alanine hydrochloride was then dissolved in 2.25 ml of dimethyl sulfoxide with the assistance of heating. OX063 trityl (180 mg) was added to achieve ~38 mM radical concentration. Fifty microliters of this mixture was pipetted into a sample cup.

For the pyruvate sample, [1-^13^C]pyruvic acid was mixed with the appropriate mass of AH111501 trityl (GE Healthcare; CID 11607875) to achieve a 30 mM radical concentration, of which 18 μl was pipetted into a sample cup. Dissolutions were performed with either D_2_O (irradiated alanine) or a D_2_O solution containing a predetermined amount of NaOH to neutralize any excess acid present (trityl + alanine HCl), yielding a pH ~7 solution in both cases.

### Dynamic nuclear polarization

Samples were polarized at 3.35 or 6.7 T using HyperSense (Oxford Instruments) or SpinAligner (Polarize), respectively. Further details are provided in text S6.2.

### In vitro experiments

The polarization levels and nuclear *T*_1_ relaxation times of samples were recorded using a benchtop nuclear magnetic resonance (NMR) spectrometer at 1.4 T (SpinSolve 60 ULTRA Carbon, Magritek). The accompanying software also enabled the level of polarization to be estimated at the point of dissolution.

### In vivo experiments

Two healthy male Sprague-Dawley rats were injected with one shot of each type of hyperpolarized alanine sample via a tail vein catheter. The time between each injection was ~2 hours. The respiratory rate and body temperature of each rat were monitored using an MRI-compatible small-animal monitoring system (Small Animal Instruments Inc., USA) while the rats were under anesthesia with 2.5 to 3% sevoflurane in medical air (1 liter/min). Both rats were scanned using a dual-tuned ^13^C/^1^H volume rat coil at 3 T (GE MR750, GE Healthcare). One rat was scanned using a slice-selective spectroscopic acquisition, and the other was imaged using a 2D MRSI sequence, echo planar spectroscopic imaging, using fidall (*116*).

Prior to these scans, a urea phantom was used to calibrate the applied flip angles, and proton images were acquired for anatomical reference. For the slice-selective spectroscopic acquisition, 80-mm slice thickness was used to ensure that the whole abdomen of the rat was imaged. One milliliter of hyperpolarized sample was injected over ~5 s, and the scan started upon the start of the injection. A 30° flip angle was applied with a TR of 3 s for a total of 5 min (100 time points). For the MRSI, 1 ml of hyperpolarized substrate was injected over ~5 s, and the scan started upon the start of the injection. A 16-by-16 matrix was chosen, and a 15° flip angle was applied with a TR of 64 ms for a total of 2 min (8 time points and ~16 s per time point). The pH of the injected solutions was measured to ensure physiological compatibility, with values ranging from 6.7 to 7.4, well within the acceptable range for intravenous administration. All animal experiments were conducted following appropriate independent ethical review and under appropriate licensing regimes in Denmark. This study was conducted under license number 2019-15-0201-00387, approved by the Animal Experiments Inspectorate, Ministry of Food, Agriculture and Fisheries of Denmark.

### Electron paramagnetic resonance

An X-band continuous wave (CW) spectrometer (EMXmicro, Bruker BioSpin; super high sensitivity rectangular cavity probe head) was used to record EPR spectra at room temperature. For recording spectra at 5 K and measurement of *T*_1*e*_ and *T*_2_, an X-band pulsed spectrometer (E680, Bruker BioSpin) was used. The chromium(III) impurity in magnesium oxide powder, a common standard in EPR (*117*, *118*), was used as a frequency reference. Further details are provided in text S6.7.

Samples that comprised dry powder were directly loaded into 4-mm-thin wall quartz EPR tubes, and their mass was recorded. For repeated measurements, samples were rotated about their own axis by ~25° to 35°. Liquid-state samples were first loaded in 2-mm-diameter capillary tubes, which in turn were loaded into the EPR tubes.

Spin counts were determined from EPR spectra using an in-house–developed MATLAB script. The script incorporated baseline correction, normalized for differences in resonator *Q*-factor, and integrated the recorded spectra.

^13^C spectra were fitted via a constrained Bayesian optimization routine (*60*). The linewidths and shapes of spectra were also compared to models built with the “pepper” function in EasySpin, a validated quantum mechanical spectral simulation library designed for EPR (*119*).

### X-ray diffraction

XRD was conducted using copper *K*_α_ x-rays, of wavelengths 1.5405982 and 1.544497 Å, in an Empyrean (Malvern Panalytical) diffractometer. XRD samples comprised dry alanine powder or an alanine powder/glycerol mix (1:1, w/w). The Cu *K*_β_ wavelength was removed from the diffraction signal with a Ni filter placed in front of the PIXcel1D detector. A set of programmable divergence slits were used for the incident optic, set to illuminate a fixed surface area of 1 cm^2^ across the angular range of the scan. During the scans, the samples were rotated about the azimuthal axis at a frequency of 0.5 Hz to fully characterize the crystallite distribution.

XRD data were compared to a previously reported crystal structure of alanine, obtained via neutron scattering (*70*), and a Rietveld refinement was undertaken in MAUD using a spherical harmonic texture basis (*68*). Further details are provided in text S6.9.

## Supplementary Materials

### The PDF file includes:

Supplementary Text Figs. S1 to S14 Tables S1 and S2 Legends for data S1 and S2 References
