# Supplementary material for: Stable electron-irradiated [1-13C]alanine radicals for metabolic imaging with dynamic nuclear polarization
Source: Sci Adv. 2025 Nov 21;11(47):eadz4334. doi: 10.1126/sciadv.adz4334 (PMC12637281; doi:10.1126/sciadv.adz4334)
Supplement: Supplementary file 1 — Supplementary Text Figs. S1 to S14 Tables S1 and S2 Legends for data S1 and S2 References [file sciadv.adz4334_sm.pdf]

Science Advances
AAAS

Supplementary Materials for

Stable electron-irradiated [1-^{13}C]alanine radicals for metabolic imaging with dynamic nuclear polarization

Catriona H. E. Rooney *et al.*

Corresponding author: Jack J. Miller, jack.miller@physics.org

Sci. Adv. **11**, eadz4334 (2025)
DOI: 10.1126/sciadv.adz4334

The PDF file includes:

Supplementary Text
Figs. S1 to S14
Tables S1 and S2
Legends for data S1 and S2
References

Other Supplementary Material for this manuscript includes the following:

Data S1 and S2

6 Supplemental Experimental Methods

6.1 Irradiation

Full technical details of the linac are published elsewhere and much of its construction details are freely available at `https://users.ox.ac.uk/~atdgroup/technicalnotes/`.

Renderings of the sample holder used to hold the sample during the irradiation are shown Fig. S1**A**. To produce the samples, a block of aluminium with two sample spaces was loaded with dry alanine powder. The powder was secured in place by taping two ordinary glass microscope slides either side of the aluminium block. The glass-aluminium-glass sandwich was held in an upright position by microscope slide spring clips within another aluminium component that itself was connected to a high precision linear slider. The integration of the slider and two stoppers into the design facilitated the centring of each sample space within the beam path. To increase the homogeneity of the irradiation exposure across the total volume of each sample space, the glass-aluminium-glass sandwich was flipped 180° after half of the total desired irradiation dose had been delivered. Irradiation doses over the range 10–100 kGy were applied to produce samples with a range of different endogenous radical concentrations.

A radiation transport simulation was undertaken in TOPAS, (*120,121*) an open-source radiation transport code optimised for medical physics applications based on Geant4, written by CERN. (*122*) As shown in Fig. S1**B, C**, this was used to optimise dose distribution across the sample with regards to the 'pile up' dose deposited either in the glass coverslips or aluminium target; the preferred deposition function is that of a 'top hat' within the sample but it was found that appropriate spacing and modulation of design produced a reasonable distribution function (Fig. S1**D**). A significant amount of charge transfer is expected to take place within the electron bombardment process. In order to obtain a more homogeneous dose distribution within the sample, we rotated the sample by 180 ° in the middle of the irradiation process; after irradiation, the sample was mixed by hand, further homogenising it.

6.2 DNP

DNP was performed either using an Oxford Instruments HyperSense polariser at 3.35 T or a customised SpinAligner 6.7 T polariser. In both cases either microwave sweep profiles were obtained by iterating over microwave irradiation frequency, or polarisation build-up curves in which a low flip angle pulse was repeatedly played and polarisation monitored. The carbon Larmor frequency at both field strengths were 35.96048861 MHz and 71.816528 MHz respectively. Amplitudes obtained and reported as signal during

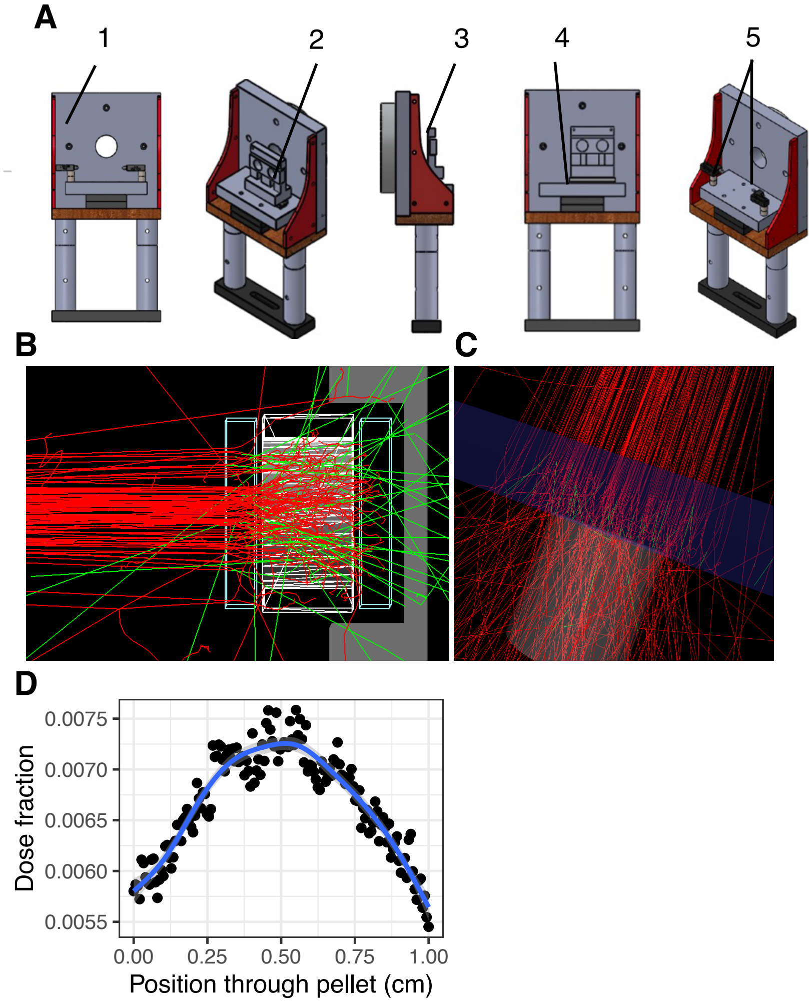

Figure S1: Details of the overall process of e-beam irradiation. **A:** A sample holder for the irradiation of alanine samples using a 6 MeV high-dose rate electron linear accelerator was designed. Renderings of the sample holder comprising (1) an aluminium endplate with a 5 cm diameter aperture for the electron beam, (2) a rectangular block of aluminium — with two cylindrical holes each acting as a sample space — interposed between two glass microscope slides and secured upright with microscope slide spring clips to (3) an L-shaped aluminium block attached onto (4) a high precision linear slider. (5) Two stoppers were positioned either side of the slider to facilitate the positioning of each sample. **B, C:** Simulated Monte-Carlo radiation transport characteristics of the designed target demonstrating appropriate dose deposition inside the compressed alanine pellet (red denotes leptons; green photons); together with **D:** predicted dose deposition as a function of depth, normalised such that the total dose is unity.

microwave sweeps or build-up curves are integrated over the well-defined NMR peak present in the 13C NMR signal as is commonly performed with DNP.

Reported time-constants for the DNP build-up profiles were fitted to a biexponential curve given by the equation

$$y = P\left(A\left(1 - e^{-\frac{\text{time}}{T}}\right) + (1 - A)\left(1 - e^{-\frac{\text{time}}{\tau}}\right)\right) \tag{S1}$$

where P is the build-up constant, A and $1 - A$ are the relative weightings of the fast and slow components of the polarisation build-up, and T and τ are the fast and slow components of the build-up times.

For the HyperSense, microwave frequency sweeps were acquired for each sample between 93.75 to 94.195 GHz in 10 MHz steps with a two minute build-up per frequency step. The microwave power was held constant at 100 mW for all microwave frequency sweeps and build-ups. The build-up of polarisation was subsequently monitored at the peak frequency of the microwave frequency sweep every three minutes using a low flip-angle readout. A 3.35 T frequency sweep is shown in Figure S2 together with the corresponding sweep for an alanine + OX063 sample. A small potential enhancement lobe may be seen at 93.970 GHz. Black dashed lines denote equivalent features in the 6.7 T microwave sweep curves, scaled exactly for field. Given the considerably less sophisticated microwave and RF architecture of the HyperSense compared to the SpinAligner, the possibility that this lobe at 93.970 GHz is artefactual remains, although it was not observed in the control sample.

Based on previous optimisation work and the use of a narrow line width radical, the SpinAligner was used without microwave modulation for the non-irradiated samples. For the irradiated samples, microwave modulation was applied over a range of 25 MHz about the given centre frequency, sinusoidally varied with the frequency of microwave irradiation occurring at a rate of 1 kHz. This applies a weak modulation and attempts to invert a greater proportion of electron spins; it also is recommended by the microwave source manufacturer in order to promote greater thermal stability of their system.

For the SpinAligner, microwave frequency sweep profiles were acquired using 32 averages, an RF transmit pulse width of 2 μs, and a TR of 30 s for irradiated samples or a TR of 10 s for all other sample types. The build-up profiles were acquired using 2 μs transmit RF pulses with a TR of 60 s at 20 mW microwave power and no averaging for all sample types. The transmission power of the RF pulses was 32 dBm, although the exact B_1 generated inside the sample cavity is poorly characterised.

The NMR spectra recorded during microwave frequency sweeps and polarisation build-ups were quantified by peak integration in MATLAB (ver. 2023a; The Mathworks, Inc). Polarisation estimates were obtained in the liquid state both hyperpolarised and at thermal equilibrium, and then back-calculated using the mea-

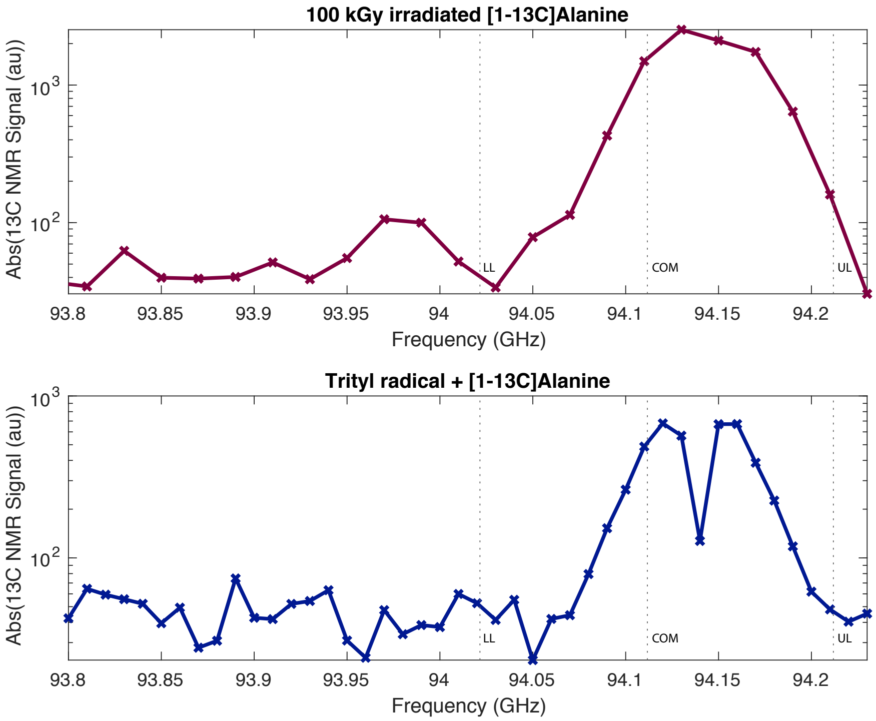

Figure S2: The frequency sweep obtained at 3.35 T for both alanine and trityl radical and 100 kGy irradiated alanine. The parameters noted "LL" (lower limit), "UL" (upper limit) and "COM" (centre of mass) are presented for illustrative comparisons with higher field DNP data, and have been defined phenomenologically as the lower, centre and upper region of features in the 6.7 T curve scaled for the exact difference, i.e. the corresponding down-scaled values of [187.77, 187.95, 188.15] GHz at 3.358 T.

sured T_1 and known time of dissolution to estimate solid-state polarisation; for samples with low nuclear polarisation in the solid state (where relative error post dissolution is likely higher or sufficiently high to be inaccurate) a direct linear scaling of ADC values obtained at constant gain with a known polarisation reference were used.

6.3 Power characterisation at 6.7 T

To address potential concerns regarding the optimisation of microwave irradiation conditions, we performed extensive calibration experiments investigating the effects of microwave power, and, for our source in the SpinAligner, frequency modulation and pulse timing parameters on the DNP enhancement profiles. These experiments were essential given the high-field (6.7 T, 188 GHz) operation of our system, where microwave field inhomogeneity within the sample cavity becomes increasingly problematic (*32*). This is because the SpinAligner's microwave cavity (diameter 28 mm, height 30 mm) is highly overmoded at 188 GHz, precluding meaningful quality factor measurements that may come with unequal power deposition into the sample.

Additionally, owing both to the closed-cycle cryogenic nature of the machine and additional hardware constraints, continuous-wave irradiation was not feasible due to thermal instabilities in both the microwave source and the sample environment. This necessitated the pulsed modulation approach, which while effective for our current studies, should not be confused with (and, indeed, precludes) the investigation of coherent polarisation transfer mechanisms such as TOP-DNP (*123*) or NOVEL sequences (*124, 125*) that might further optimise the enhancement – our modulations are orders of magnitude slower than the electron Larmor frequency.

Systematic power-dependent measurements were performed on 70 kGy electron-irradiated ^{13}C-alanine samples using our "standard conditions" that were used in the majority of this work (25 MHz amplitude modulation, 100 Hz frequency modulation, both detailed subsequently). Figure S3**A** shows frequency sweep profiles acquired at 10 mW and 20 mW output power, demonstrating that the characteristic asymmetric enhancement profile is preserved across this power range. The build-up kinetics (Figure S3**B**) show increased absolute signal intensity at higher power, consistent with more efficient microwave saturation of the electron spin transitions, but the fundamental biexponential character and relative time constants remain unchanged.

The SpinAligner system employs pulsed microwave irradiation with frequency modulation to accommodate the broad EPR linewidth of the stable alanine radicals while avoiding continuous-wave heating limitations of the 188 GHz source. The user-specified parameters are detailed below:

- **AM (Amplitude Modulation)**: The frequency sweep bandwidth in MHz. This determines the range

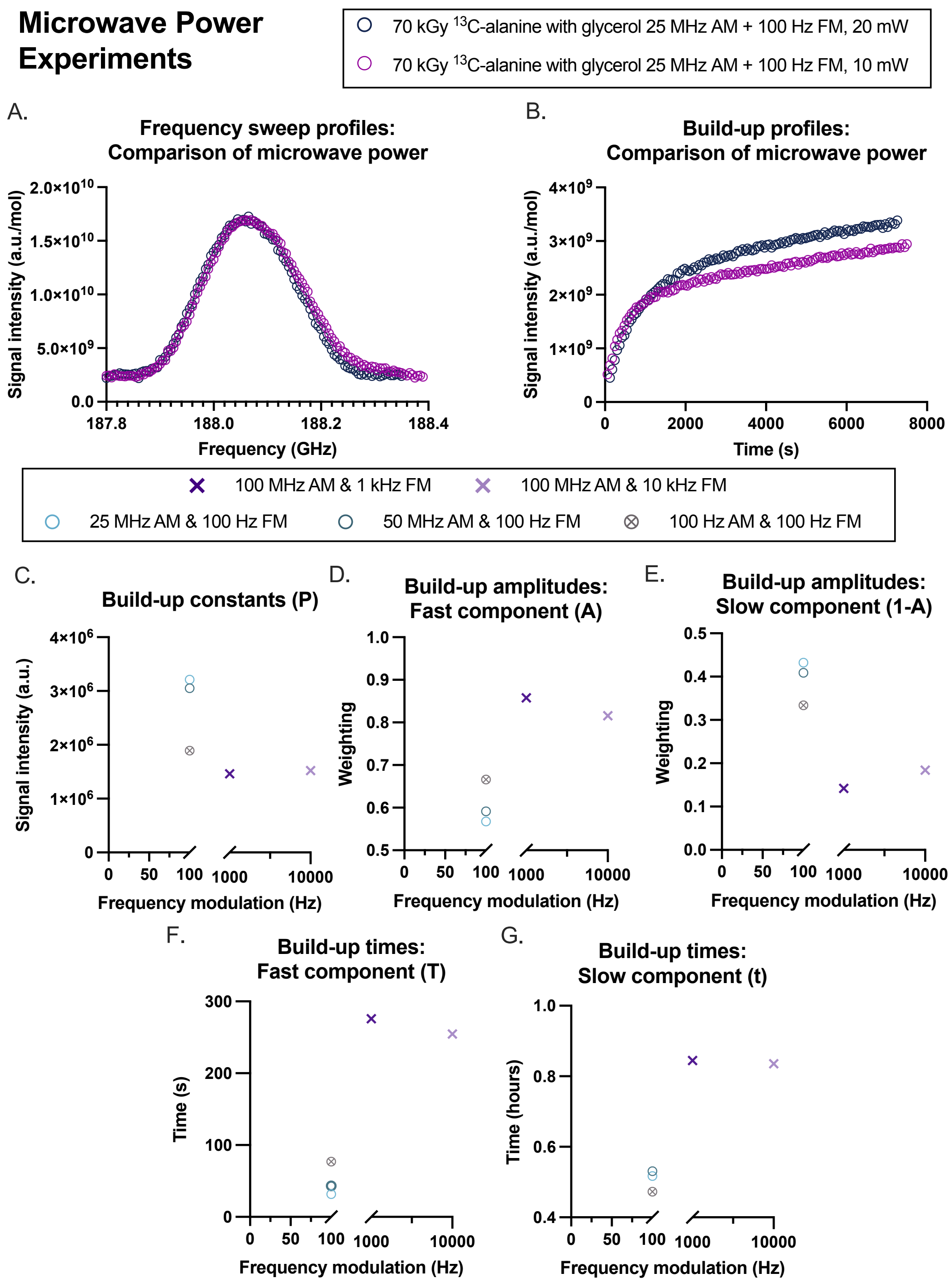

Figure S3: (**A**) Frequency sweep profiles comparing 10 mW and 20 mW microwave power showing preserved asymmetric enhancement, and marginally enhanced build-up. (**B**) Build-up profiles demonstrating increased absolute signal at higher power with largely unchanged kinetics. (**C–G**) Systematic investigation of frequency modulation parameters showing optimal conditions near 100 Hz modulation rate with preserved biexponential build-up characteristics across all conditions tested.

of microwave frequencies scanned during each pulse (e.g., 25 MHz spans ±12.5 MHz around the center frequency).

- **FM (Frequency Modulation)**: The base modulation rate in Hz. This parameter, combined with hardware duty cycle constraints, determines the pulse repetition timing.

The relationship between these user parameters and actual pulse timing is governed by the microwave synthesiser hardware implementation. For combined PM+FM operation, the pulse timing follows:

$$t_{\text{pulse on}} = t_{\text{off,displayed}} + \frac{10^6\ \mu s/s}{f_{\text{FM}}} - 40\ \mu s \tag{S2}$$

$$t_{\text{pulse off}} = t_{\text{on,displayed}} + 40\ \mu s \tag{S3}$$

where f_{FM} is the FM parameter in Hz, and the displayed on/off times are hardware-inverted values read from the synthesiser. The term $10^6/f_{\text{FM}}$ represents the base period in microseconds corresponding to the FM rate.

For our standard conditions (AM = 25 MHz, FM = 100 Hz), these equations yield:

$$t_{\text{pulse on}} = \frac{10^6}{100} - 40 = 9960\ \mu s \approx 9963\ \mu s\ \text{(measured)} \tag{S4}$$

$$t_{\text{pulse off}} = 48139\ \mu s\ \text{(measured)} \tag{S5}$$

This corresponds to a duty cycle of approximately 17% and a total cycle time of 58 ms. The discrepancy between the theoretical base period (10 ms for 100 Hz) and actual cycle time reflects hardware-imposed timing constraints and thermal management requirements.

During each "on" period, the microwave frequency is swept linearly across the AM bandwidth (e.g. 25 MHz) in discrete steps. The number of frequency steps and dwell time per step are determined by the synthesiser's internal timing, typically resulting in 25–50 discrete frequency points per sweep. This approach aims to ensure that the broad EPR linewidth (∼15 mT FWHM, corresponding to ∼420 MHz at 6.7 T) is broadly saturated while maintaining sufficient power at each frequency point to drive polarisation transfer.

We systematically investigated the effects of both amplitude modulation bandwidth (25–100 MHz) and frequency modulation rate (100 Hz to 10 kHz) on the enhancement characteristics. Figure S3**C–G** summarises the parametric dependence of the biexponential build-up characteristics on modulation frequency. Although very preliminary values and potentially specific to our hardware, the build-up constant P (panel C) shows optimal values near 100 Hz modulation, with diminishing returns at higher frequencies. This behavior likely

Table S1: Spin-count data for EPR stability analysis of three replicate samples each of irradiated alanine dispersed in glycerol, measured over time. This is plotted graphically in Fig. 2**B**.

Week	30 kGy			50 kGy			70 kGy		
0	3340	2910	3550	5590	4100	3370	6210	5420	4970
2	5070	2110	2800	5590	4740	4000	5070	4070	5460
4	2120	4030	4580	4990	6190	3740	5900	5390	4740
16	2780	2130	1890	4300	2360	3100	5100	3760	5040

reflects the interplay between the EPR linewidth (~15 mT FWHM), the frequency sweep range (25 MHz), and the electron spin relaxation times under our experimental conditions.

Importantly, the relative weightings of fast and slow build-up components (panels D and E) and their respective time constants (panels F and G) show some systematic but modest variations with modulation parameters. The fast component time constant T remains in the range 300–400 s across all conditions tested, while the slow component t varies between 0.5–2 hours (the upper end of the build-up experiment tested). We believe this rules out, for example, a finite microwave bandwidth leading to differential enhancement, and we further note that the pyruvic acid frequency sweep curve under these conditions is a well-resolved bimodal curve of equal intensity within this region.

6.4 Stability analysis

Samples of irradiated alanine and glycerol were created on or the day after irradiation, and stored in borosilicate glass haematocrit tubes, themselves stored in a falcon tube. These were stored in separate home-made desiccators consisting of monolayer of silica desiccant gel beads (Sigma Aldrich, UK) in a plastic ‘tupperware’ self-sealing container. Three repeated EPR measurements were obtained by inserting the haematocrit tubes inside an EPR tube, and were rotated by hand within the machine between acquisitions. EPR spectra were obtained at weeks 0, 2, 4, and 16 after irradiation. A simple linear regression approach was used for 30, 50 and 70 kGy irradiated samples dispersed in alanine; there was no significant change with time (p = 0.14, 0.085, 0.18, respectively) and the goodness of fit was poor (R^2 = 0.20, 0.27, and 0.17 respectively). We interpret this as providing evidence of stability as there was no significant change of EPR signal intensity with time.

The raw data behind the stability plot is given below, in Table S1

6.5 Polarisation characterisation

As summarised in Tab. S2, we found that an approximately inverse relationship between build-up time constants and irradiation dose, i.e. the greater the radical concentration, the longer the slow component of the build-up time and the shorter the fast component.

This is potentially because of the competing effects of increased polarisation transfer efficiency and enhanced nuclear relaxation at higher radical concentrations. The shortened fast component likely reflects more efficient initial polarisation transfer due to increased electron-nuclear spin coupling, while the extended slow component may indicate limitations in long-range spin diffusion as paramagnetic centres become more densely packed. At higher radical concentrations, the shortened inter-radical distances could impede the cooperative network of nuclear spins necessary for efficient polarisation distribution throughout the sample, creating isolated 'islands' of polarisation around each radical centre. This spatial heterogeneity in the polarisation landscape would manifest as different characteristic time constants in the bi-exponential build-up curve.

At very high radical concentrations, it may be the case that the electronic environment in the crystal is similar to that of a doped semiconductor with a complex band-structure, at which point existing theories of DNP are likely not complete.

Table S2: Where T is the fast component of the build-up time and t is the slow component of the build-up time.

Radiation dose (kGy)	Build-up Amp: P (a.u.)	T (s)	t (hours)
30	1520000	22.9	0.92
50	2280000	22.2	2.69
70	5110000	14.9	6.88

6.6 Imaging experiment details

The EPSI sequence used is described in detail elsewhere (*116*) and was intended to both show the spatial localisation of the hyperpolarised contrast agent and additionally be sensitive (i.e. able to resolve) any labelled impurity peaks that may be present. The acquisition matrix size was 16×16 (spatial) $\times$ 256 (spectral); in an 80×80 mm^2 field of view; slice thickness: 30 mm; spectral bandwidth: 5000 Hz; for a spectral resolution of 19.5 Hz/point prior to reconstruction. Data were reconstructed with zerofilling by a factor of two in all dimensions. The TR was 16.0 seconds; TE: 2.642 ms; with a 15° flip angle. Eight images were acquired.

6.7 EPR Supplementary Methods

Quantitative EPR spectra were recorded on an X-band continuous wave (CW) EPR spectrometer (EMXmicro, Bruker Biospin GMBH) using an ER 4122SHEQ resonator at room temperature; low temperature measurements, including electronic relaxation constants T_{1e} and measured T_{2e} (known as T_m) measurements were performed at 5 K on an X-band pulsed E680 spectrometer. Magnesium Oxide (MgO) powder, a common standard reference in EPR, (*118*) was used to account for any field offset in the experimental data, arising on physical grounds, which was calculated at $\sim$ 0.6 mT. Furthermore, it is known that the signal amplitude is proportional to $\chi_m Q \sqrt{\text{Microwave power}}$ in EPR and these parameters were controlled appropriately. (*117*)

Samples that comprised dry powder were directly loaded into 4 mm thin wall quartz EPR tubes and their mass recorded. For repeated measurements, samples were rotated about their own axis by approximately 25 to 35 ° to ensure adequate powder averaging. Liquid state samples were first loaded in 2 mm diameter capillary tubes which in turn were loaded into the EPR tubes.

All solid state samples used the standard set of parameters listed below where only frequency changed between samples. The frequency has been normalised to for all data sets presented. Scans other than at 5 K were performed at room temperature. In brief, spectrometer parameters were: microwave attenuation = 36 dB; microwave power = 0.05024 mW; scans = 1; centre field = 333.8 mT; sweep width = 33 mT; sweep direction = up; sweep time = 40.04 s; time constant = 20.48 ms; conversion time = 14.46 ms; modulation amplitude = 1.2 G; modulation Frequency = 100 kHz; sweep rate = 0.816 mT/s. Echo Detected field sweeps for gamma irradiated alanine were recorded at 5 K using a standard Hahn echo pulse sequence ($\frac{\pi}{2} - \tau - \pi - \tau -$ detect) with $\pi/2$ pulse length of 16 ns, π pulse length of 32 ns and τ = 300 ns, a shot repetition rate of 1.024 s was used corresponding to a sweep rate of 0.2 mT/s with 1 shot per point and one average used. For ease of comparison to CW data this field swept data is presented in the derivative form in the main text. T_{1e} was measured by fitting to a functional form $y = c - ae^{-\frac{x}{b}}$, where b is the T_1 time, using the "`fit`" function in Matlab, performing a least-squares fit. The field sweep was for the sample containing OX063 was measured using free induction decay detection with a 1000 ns pulse followed by a detection period of 600 ns with a shot repetition rate of 0.2 s with 2 shots per point and a sweep width of 3 mT corresponding a sweep rate of 0.06 mT/s. Considering a g-value of 2.00319 it was found that this system had a field offset of 0.624 mT and spectra have been corrected accordingly.

For longitudinal studies observing the stability of radical species, EPR spin counts were recorded on the same day as their irradiation and then two weeks, four weeks, and 16 weeks post irradiation. Dry powder samples were prepared directly in the EPR tubes and rotated about their own axis as above; the mass of empty

and filled tubes were recorded and monitored over time and data are corrected for the mass of samples present. Solutions containing glycerol were prepared as stock solutions which were used to fill haematocrit tubes which themselves were inserted into the EPR tubes. Best practices for undertaking quantitative EPR spectroscopy were followed, such as the use of an appropriate resonator, and reproducible packing metrics with impurity controlled materials. (*126, 127*) EPR data was analysed in Matlab. Spin counts were determined by double numerical integration of the EPR spectra. First, the raw spectra were baseline-corrected using a first-order correction, followed by integration to obtain absorption spectra. Q-value normalisation was performed to that using a reference Q-value of 8000 (obtained with the MgO sample) to account for variations in cavity loading. A polynomial fit (order 15) was then applied to specific field regions (317.5–333 mT and 337–350 mT) for secondary baseline correction. The final double integration value was calculated from the fully corrected absorption spectrum, with verification performed by comparing the numerical derivative of the absorption spectrum against the original intensity data. Full width at half maximum (FWHM) values were also calculated for each spectrum to characterise line broadening effects related to radical concentration and interactions.

For conversion between spin counts and radical concentrations, a calibration curve was established using TEMPO samples of known concentration, allowing quantitative determination of radical concentration in alanine samples.

The T_{1e} for gamma irradiated alanine at 5 K recorded at the center of the observed signal was measured using an inversion recovery experiment with the sequence $\pi - t - \frac{\pi}{2} - \tau - \pi - \tau -$ detect, with $\pi/2$ pulse length of 16 ns, π pulse length of 32 ns and $\tau = 300$ ns. The measured T_1 was greater than the allowed maximum shot repetition rate of the spectrometer and thus the command `tsleep` was used to generate the inter pulse delay `t` which gave an effective shot repetition rate of 60 s. The measured value of T_{1e} for the SAR at 5 K in glycerol was 7.59 s, and $T_m \approx 332\ ns$. For comparison, a "literature prep" (non-irradiated [1-13C]L-alanine, 18.94M NaOH, DMSO, 15 mM OX063 and 0.3 mM Dotarem gadolinium as Gd(III)) had $T_{1e} = 39\ ms$ (using a standard inversion recovery with a shot repetition rate of 0.25 s), $Tm = 378\ ns$. A "literature prep without gadolinium", i.e. just OX063, using a shot repetition rate of 9 s and `tsleep` had $T_{1e} = 1.41\ s$ and $T_m = 370\ ns$. These fits are shown below, in Fig. S4. Raw data is provided as further SI to this manuscript.

As shown in Fig. S5, no significant difference in dose-response curves was observed with [1-^{13}C]alanine compared to natural abundance alanine. This is largely expected because the predominant method of energy loss at 6 MeV is via Compton scattering, with a cross section on the order of millibarns. In contrast, electron-nuclear interactions such as electro-disintegration or nuclear excitation have microbarn to nanobarn cross sections at these energies, and have been extensively studied (*128–130*) as a probe of differences

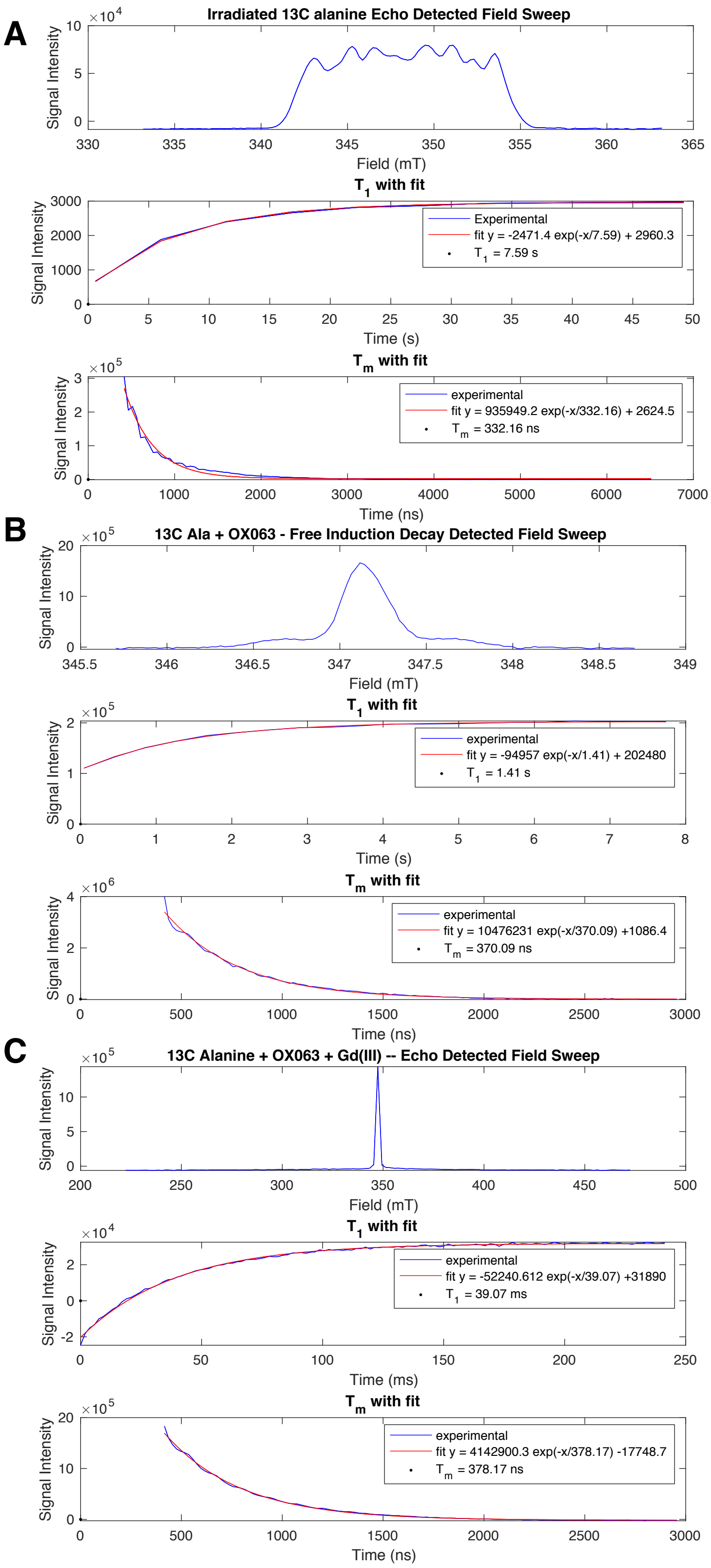

Figure S4: Electronic T_{1e} and T_m measurement data and fits, obtained at 5 K, for **A**: irradiated 100 kGy alanine; **B**: a "literature prep" trityl radical without gadolinium; and **C**: a "literature prep with gadolinium" sample.

in the radii of ^{12}C and ^{13}C nuclei (a difference of approximately 0.023±0.01 fm). (*131*) Furthermore, the mass-energy absorption coefficients for ^{12}C and ^{13}C differ by only ~0.08% due to the marginal difference in electron density per unit mass. (*132*) Therefore, any isotopic effect on dose deposition would likely be within experimental uncertainty of our measurements.

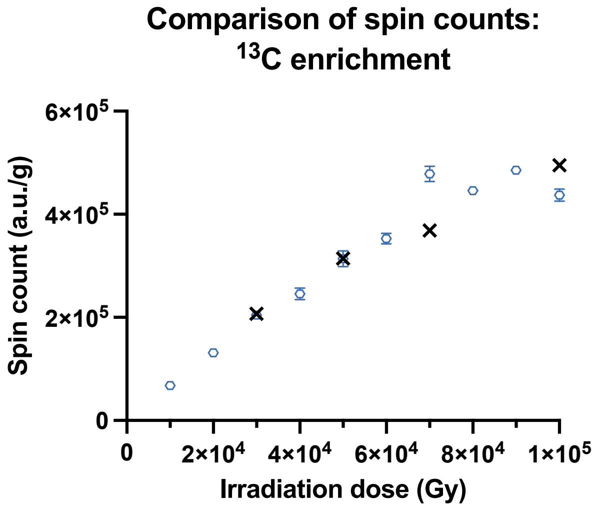

Figure S5: Spin counts recorded from ^{13}C-alanine (black crosses, n=3) were consistent with those recorded from NA alanine (blue hexagons, n=4) at irradiation doses 30, 50, 70, and 100 kGy.

6.8 Alanine SAR EPR Fitting and EPR Simulation Details

To try to fit the EPR data obtained of irradiated powder ^{13}C alanine, a detailed simulation was undertaken in EasySpin using appropriate previously published hyperfine constants and g-tensors for the radical R1 only, which is responsible for the majority of the signal recorded via EPR. This was motivated by a desire to have a single radical species for further large-scale spin-density simulations (although we note that a semi-empirical approach was additionally undertaken, using purely the experimental EPR spectrum scaled for field). The initial fitting model included a spin system with an $S = 1/2$ electron and a g-tensor consistent with the R1 alanine radical ($g = [2.0041, 2.0034, 2.0024]$). The model incorporated hyperfine couplings for five protons (four β-protons and one α-proton) with starting values derived from NA alanine, plus the ^{13}C nucleus . These are stated in full in the attached analysis code in Data S2. We employed the `pepper` function provided by EasySpin (*119*) with a hybrid method for orientational averaging, using an adaptive grid approach with an initial grid size of `[10 2]` and a minimum grid size of 2. This was progressively refined throughout the optimisation process. Isotropic line broadening from unresolved interactions was modelled

with a peak-to-peak linewidth (`lwpp`) parameter of 0.45 mT.

The experimental spectra were first-derivative spectra collected at an X-band microwave frequency of approximately 9.4 GHz (with the exact value extracted from experimental parameters) with a field range covering 330-350 mT. Spectra were baseline-corrected prior to fitting by subtracting the mean of the first 128 points.

Fitting was performed using a two-stage optimisation approach. First, we employed Matlab's `surrogateopt` function with 10,000 maximum function evaluations, exploring a bounded parameter space centred around initial estimates. The objective function combined both spectral and integral matching metrics to ensure proper reproduction of both line shapes and relative intensities. Specifically, we minimised:

$$\min_{\theta} \|\mathcal{I}_{\text{fit}}(\theta) - \mathcal{I}_{\text{exp}}\|_2 + \|\mathcal{S}_{\text{fit}}(\theta) - \mathcal{S}_{\text{exp}}\|_2 \tag{S6}$$

where $\mathcal{I}$ represents cumulative integrals of spectra and $\mathcal{S}$ represents the normalised spectra themselves.

The final fit was further refined using Bayesian optimization (`bayesopt`) with additional iterations (150) to ensure convergence to the global minimum. The fitting achieved satisfactory spectral reproduction with the dominant R1 species, though slight deviations suggest potential minor contributions from R2 and R3 species or other structural heterogeneities in the ^{13}C-labelled samples. Full experimental metadata, simulation code, and raw experimental data are provided as supplemental data S2.

We should note that the alanine radical systems formed following irradiation by ionising radiation have been studied in depth by EPR, and many reported parameters have been obtained for both the initial high-energy primary radical, and three subsequent radical species. A reasonable fit to our data can be obtained by previously reported parameters, (*59*) shown in Fig. S6, and these are enclosed (with EPR relaxometry data) as Data S2. These fits do not include any degree of crystallographic ordering, as rotating the tube did not affect signal intensity. At low temperature, it is likely that radical species interconvert and in particular may lose direct molecular character, with some degree of between-molecule interactions possible within the molecular sites that are not equivalent in the unit cell. The low temperature data can be modelled to a moderate level using parameters derived from those published for the primary radical, (*61*) summed with smaller contributions from the known SAR species R1, R2, R3. (*55, 59*) See code in Data S2 for specific values.

With regards to partial ordering which is invoked in the analysis of the XRD and DNP datasets but not explicitly observed in the EPR data, we should further stress the degree to which sample preparation may affect this parameter. Both DNP and XRD samples are mildly compressed into their containers, either manually when entering the sample chamber (XRD) or by 3 bar helium gas as part of an atmospheric purge

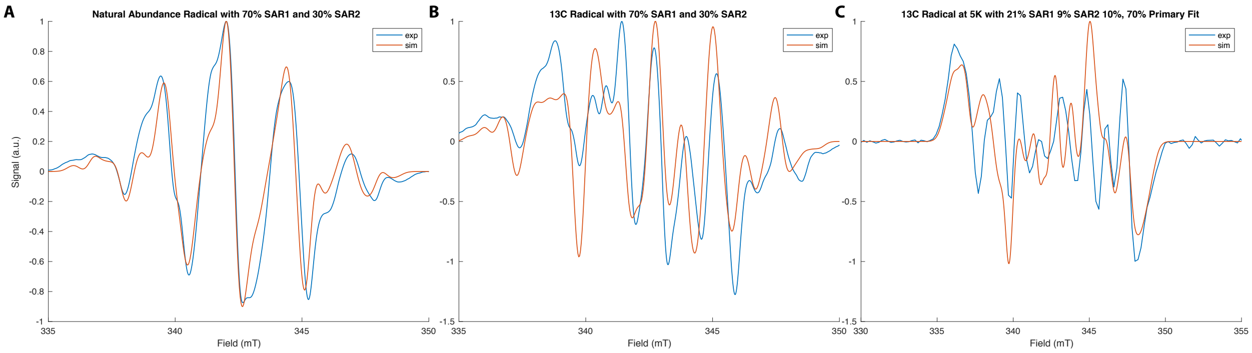

Figure S6: A demonstration of simulated literature values for reported alanine radicals. At room temperature, a moderately good fit to (**A**) natural abundance data can be obtained from a 70%/30% mixture of the radicals SAR1 and SAR2. A similar fit can be obtained for (**B**) the 13-C labelled alanine powder. At low temperature (**C**), a freely fitted mixture of R1, R2 and the primary radical can be found to approximate the shape of the experimental data. These data have been field corrected and frequency corrected to 9.6 GHz

cycle (DNP). In contrast, EPR tubes are narrow diameter and it was not readily possible to pack powder samples densely within them, which again might mean that EPR undertaken in these spectrometers is not representative of that within the DNP cryostat. Furthermore, reflecting the presence of different interaction strengths at different field and temperature combinations, the exact microscopic picture may well depend upon parameters such as the rate at which the sample was lowered and frozen in the magnetic field in the DNP polariser. Furthermore, the ordering we report in both the crystal and XRD instrument frames may well be aligned differently to that in our DNP cryostat, itself with B aligned perpendicular to the laboratory floor (unlike EPR spectrometers, where it is parallel). Although the crystallite size distribution from XRD data is consistent with microcrystallites having a narrow size distribution of mean length 580.8 ± 37.6 nm, and therefore the effect of gravity is expected to be minimal, such considerations cannot be ruled out as influencing the distribution of environments contained within the sample.

6.9 X-ray diffraction texture analysis

High-quality diffraction data was obtained with a signal-to-noise ratio of approximately 10^{12} with visually clear differences between the two samples, irrespective of the higher (expected) polynomial background due to excess liquid glycerol in the case of the sample with glycerol (Fig. S7). Quantitative Rietveld refinement was performed using MAUD (Materials Analysis Using Diffraction, version 2.9993), a validated Java-based

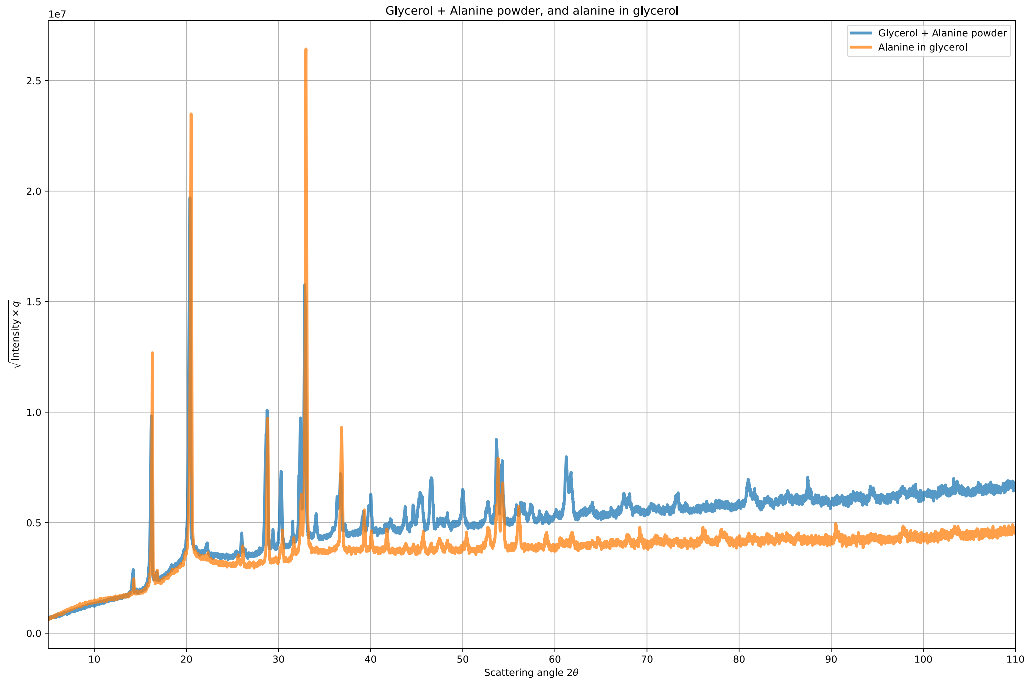

Figure S7: A comparison of (1) the algebraic sum of alanine power and separately acquired glycerol XRD data (blue), and (2) the separately acquired spectrum from an alanine powder + glycerol mixture (orange). A clear change in crystallographic texture is evident from the variation in the relative intensity of the Bragg diffraction peaks. The presence of these peaks indicates that the alanine crystallites have not dissolved in glycerol.

crystallographic software. Initially, the known orthorhombic crystal structure of L-alanine was employed as a starting model. The following parameters were systematically refined, in order:

1. Background (polynomial parameters)

2. Sample displacement and zero-offset (-0.081 ° ± 0.0009 °)

3. Unit cell parameters

4. Overall temperature factor (B-iso = 3.00±0.15 Å^2)

5. Instrumental profile parameters (Caglioti function for peak broadening)

6. Crystallographic texture

For both samples, the refinement yielded satisfactory crystallographic R-factors, with the glycerol-dispersed sample showing $R = 10.2\%$ and weighted $R_w = 14.6\%$, compared to $R = 12.8\%$ and weighted $R = 18.6\%$ for the pure powder. These values indicate good agreement between the experimental data and the structural model after accounting for texture.

Texture analysis was carried out using the E-WIMV algorithm implemented in MAUD with a spherical harmonic approach. This method involves reconstructing the orientation distribution function (ODF) from the diffraction data to quantify the degree of preferred crystallite orientation. The refinement utilised (1) an exponential harmonic texture model; (2) spherical harmonics up to order 24; (3) a regular ODF sampling grid (5 ° resolution); (4) multiple sample orientations as, during the scans, the samples were rotated about the azimuthal axis at a frequency of 0.5 Hz to fully characterise the crystallite distribution. The reconstructed ODF maps show clear texture and are illustrated in Fig. S8. This likely originates from the asymmetric nature of the orthorhombic unit cell.

The texture is represented in multiples of random distribution (mrd), where mrd = 1 indicates random orientation, mrd $>$ 1 indicates preferred orientation, and mrd $<$ 1 indicates orientation depletion; typically mrd > 3 or $< \frac{1}{3}$ are taken as evidence of a partially ordered or ordered solid. Our analysis revealed significant texture in the glycerol-dispersed sample with maximum mrd values exceeding 10 and minimum values below 0.01 in the inverse pole plot, and more than 1600 in the ODF, indicating a highly ordered crystalline arrangement within the glycerol matrix. This contrasts with the more uniformly distributed orientations in the pure powder sample.

The pole figures were generated for the principal crystallographic directions (normal direction [ND], rolling direction [RD], and transverse direction [TD]) to visualise the orientation distribution. The texture

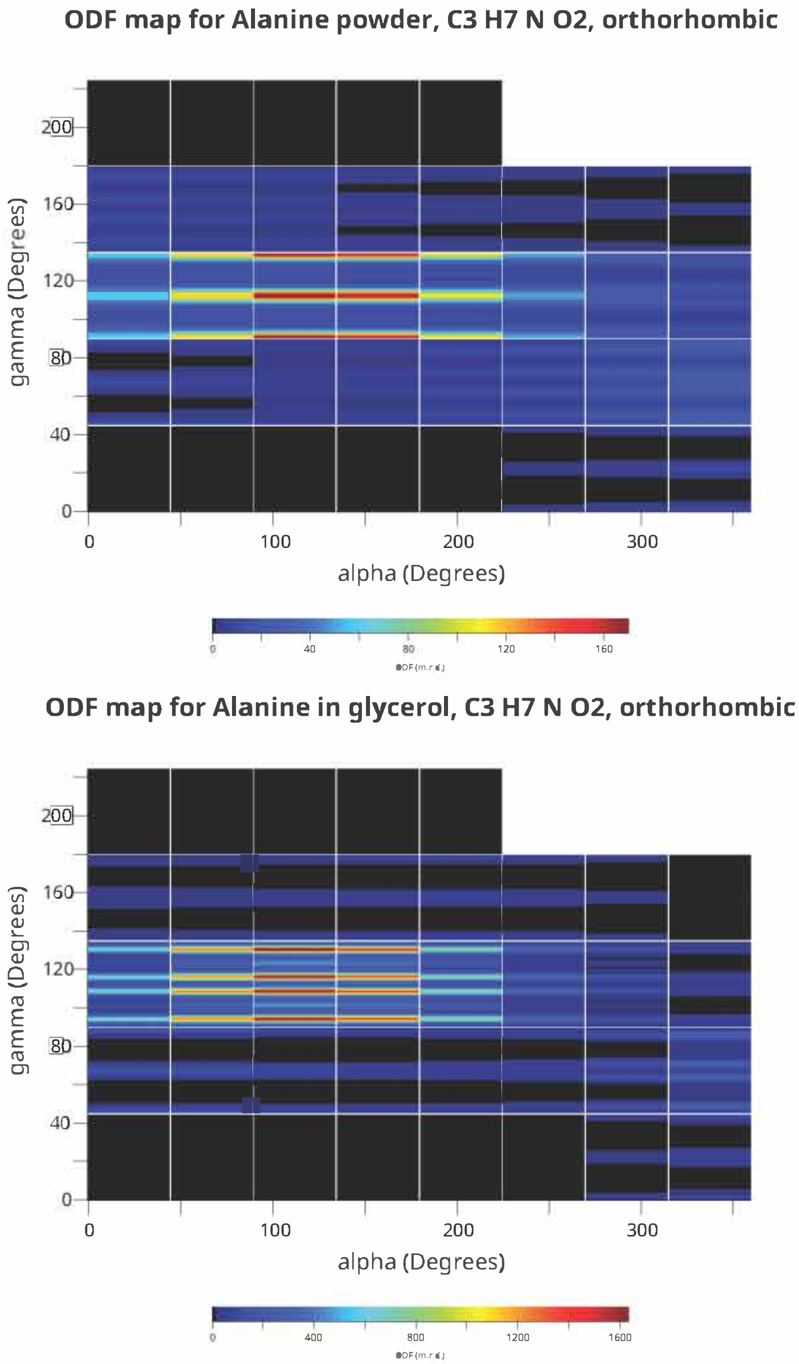

Figure S8: Orientation distribution functions for alanine and alanine/glycerol mixtures show a clear orientation preference that is more than a factor of ten times greater than for alanine alone.

analysis revealed that alanine crystals in glycerol adopt specific preferential orientations, likely due to interactions between the crystal faces and the glycerol matrix during sample preparation. The refined texture model was incorporated into the final Rietveld refinement, which significantly improved the fit to the experimental data, confirming that the observed intensity variations were indeed due to preferred orientation rather than structural factors. These data are provided as supplemental data S1.

6.10 Molecular Dynamics Simulations

Molecular dynamics (MD) simulations were performed to investigate the interactions between alanine and glycerol molecules, with particular focus on hydrogen bonding characteristics. Rather than starting from a crystalline alanine structure, we deliberately employed a randomly positioned initial configuration for several methodologically sound reasons: (1) to establish an upper bound on the mixing thermodynamics between alanine and glycerol, (2) to avoid kinetic trapping issues associated with the prohibitively slow crystal dissolution process (which would exceed our accessible simulation timescale of tens of ns), (3) to directly probe the inherent molecular interactions without the confounding variable of crystal lattice energy, and (4) to model potential amorphous states of alanine that may be relevant in rapid cooling scenarios such as arise when a sample is placed into the DNP hyperpolariser. This approach allowed more efficient phase space sampling while providing a reference point for potential future crystalline-based simulations.

The simulation system was constructed using the Enhanced Monte Carlo (EMC) package (v4.1.5), (*72*) a pre-processing module for LAMMPS. (*71*) A mixed system containing both alanine and glycerol molecules was generated with a total of 1000 molecules at a temperature of 300 K and pressure of 1 atm. We elected to have a smaller number of molecules with periodic boundary conditions and let the system equilibrate for a longer period of time. The initial densities were set to 1.424 g/cm^3 for alanine and 1.26 g/cm^3 for glycerol. The PCFF (Polymer Consistent Force Field) was employed for all simulations, as implemented in the EMC and LAMMPS packages. (*133, 134*) PCFF is a second-generation force field developed for organic molecules, polymers, and biomolecular systems, and has been extensively previously validated for simulating hydrogen bonding interactions in similar molecular systems, including alanine crystals. (*135–137*) The force field parameters were automatically generated by the EMC setup procedure. The integration timestep was set to 1 fs. Long-range electrostatic interactions were handled using the particle-particle particle-mesh (PPPM) method with a precision of 0.001. A cutoff of 1 nm was applied for both van der Waals and Coulombic interactions.

The simulation protocol consisted of the following steps:

- System initialization: The mixed alanine-glycerol system was first constructed using EMC with random molecular placements based on the specified densities in two phases
- Energy minimization: The system was energy-minimised to eliminate bad contacts using LAMMPS.
- Equilibration: The system was equilibrated in two phases:
  - Initial equilibration using a Langevin thermostat at 300 K with a damping parameter of 100 fs, combined with a distance-limited NVE integration (limit of 0.1 Å) for 1000 timesteps.
  - Production equilibration in the NPT ensemble at 300 K and 1 atm pressure with temperature and pressure damping parameters of 100 fs and 1000 fs, respectively.
- Production run: Following equilibration, a production run of 30 ns (30,000,000 timesteps) was performed in the NPT ensemble. The system state was recorded every 1000 timesteps (1 ps) for analysis. This took approximately 1 day.

Post-processing was performed in VMD, (*138*) and density and the radial distribution function computed at the end of the equilibration process (c.f. Fig. S9). It was found that liquid glycerol did not penetrate substantially into the alanine domain on the timescale of the simulation, suggesting limited miscibility between the two substances under these conditions. The overall radial distribution function $g(r)$ exhibited features consistent with liquid-phase behaviour, including a prominent first coordination shell peak at approximately 2 Å followed by diminishing oscillations that approached uniformity at larger distances. This $g(r)$ profile confirms the absence of long-range crystalline order and indicates a liquid-like molecular arrangement with short-range spatial correlations characteristic of hydrogen-bonded systems. While our simulation approach cannot definitively determine the global thermodynamic minimum of the system (which might involve a crystalline alanine phase separate from glycerol), it effectively establishes the relative stability of the mixed state and characterises the molecular interactions at the interface between alanine and glycerol domains. The persistence of distinct domains throughout the 30 ns simulation provides valuable information about the limited mutual solubility of these compounds under the studied conditions.

6.11 Spin-dynamics simulations

In an attempt to semi-empirically predict the asymmetric frequency sweep curve, a computational study on a model of the radical-damaged singular alanine crystal was undertaken. The spatial distribution of stable alanine radicals (SARs) was modelled using the known crystallographic data from the L-alanine structure

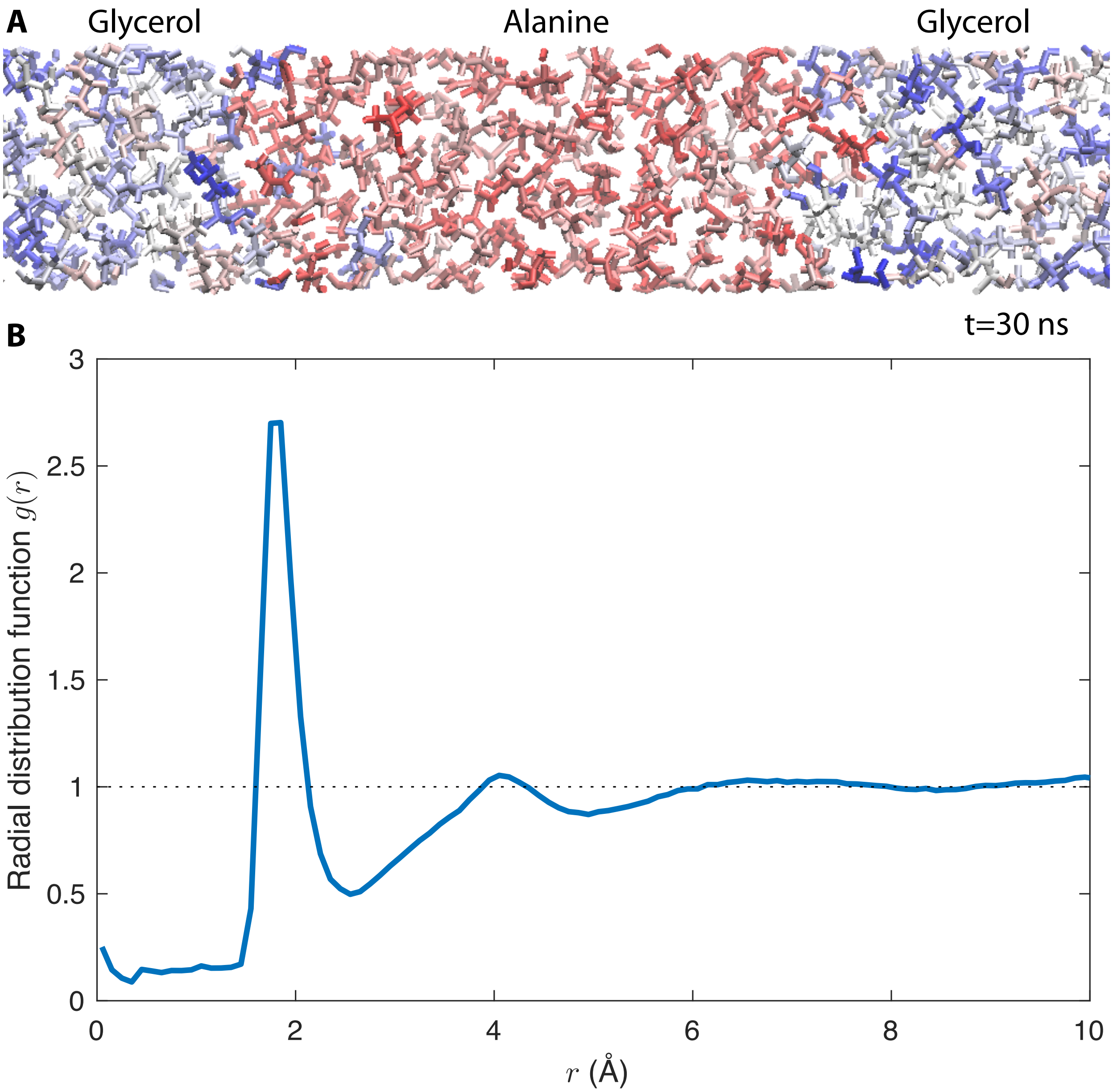

Figure S9: Molecular dynamic simulations of glycerol and alanine at 300 K. **a**: The initial two-phase configuration remained approximately stable throughout the 30 ns simulation, with limited penetration into molecular domains, indicating poor miscibility between the compounds; **b** the radial distribution function $g(r)$ for the system exhibits liquid-like characteristics with a prominent first coordination shell peak at approximately 2 Å, followed by diminishing oscillations approaching uniformity at larger distances, confirming the absence of long-range crystalline order.

provided by neutron diffraction, assuming these are unchanged by irradiation. Since electron irradiation creates radicals at random positions within the crystal lattice, we employed bootstrap sampling (10,000 iterations) of possible radical positions to determine the statistical distribution of inter-radical distances, written using ASE in Python. (*139*)

DNP simulations were performed using Spinach 2.4.5157 on the Arcus-C supercomputer with a magnetic field of 6.7 T. The basic single-electron, single-^{13}C system utilised the experimentally determined g-tensor of the stable alanine R1 radical with principal values $g = [2.0041, 2.0034, 2.0024]$ and eigenvectors from Sagstuen et al. (*55*) Relaxation was modelled using the Weizmann formalism with electron $T_1 = 10$ s, $T_2 = 0.3\ \mu$s, and nuclear $T_1 = 100$ s, $T_2 = 3$ ms at 1.4 K – the conditions under which DNP was performed. Modelled hyperfine couplings were also provided.

For systems with multiple radical centres, simulations were extended to include two electron spins with slightly different g-tensors (differing by 10^{-5} in diagonal elements) and multiple ^{13}C spins with coordinates derived from the crystal structure. We used a spherical tensor Liouvillian formalism. The swept microwave irradiation provided by our hardware was implemented as a frequency-modulated pulse sequence with the following parameters:

$$\text{pulse-on duration} = 9963\ \mu\text{s} \tag{S7}$$

$$\text{pulse-off duration} = 48139\ \mu\text{s} \tag{S8}$$

$$\text{frequency sweep bandwidth} = 25\ \text{MHz} \tag{S9}$$

$$\text{modulation rate} = 100\ \text{Hz} \tag{S10}$$

The frequency sweep was divided into 25 discrete slices spanning from -12.5 MHz to $+12.5$ MHz relative to the centre frequency. For microwave sweep simulations, we examined 10 points between 187.5 GHz and 188.5 GHz. The build-up simulations were run to a maximum time of 60 seconds, corresponding to acquired experimental data.

The powder averaging was performed using the `icos-2ang-12pts` grid for the basic simulations and `rep-2ang-100pts-sph` for more detailed models. The polarisation transfer was monitored using detection operators corresponding to the ^{13}C L_z Liouvillian terms. For extended simulations with multiple ^{13}C nuclei, we incorporated 56 carbon positions from a supercell of the alanine unit cell (which contains four molecules), with the radical species positioned at sites 21 and 5 in the lattice. The electron-electron dipolar coupling was calculated from their spatial separation within the crystal structure. Periodic boundary conditions were included. The simulation ran on 200 nodes, each containing 48 core Cascade Lake processors (Intel Xeon Platinum 8268 CPU @ 2.90GHz) with 392 GB of ram, and took approximately 4 days to complete.

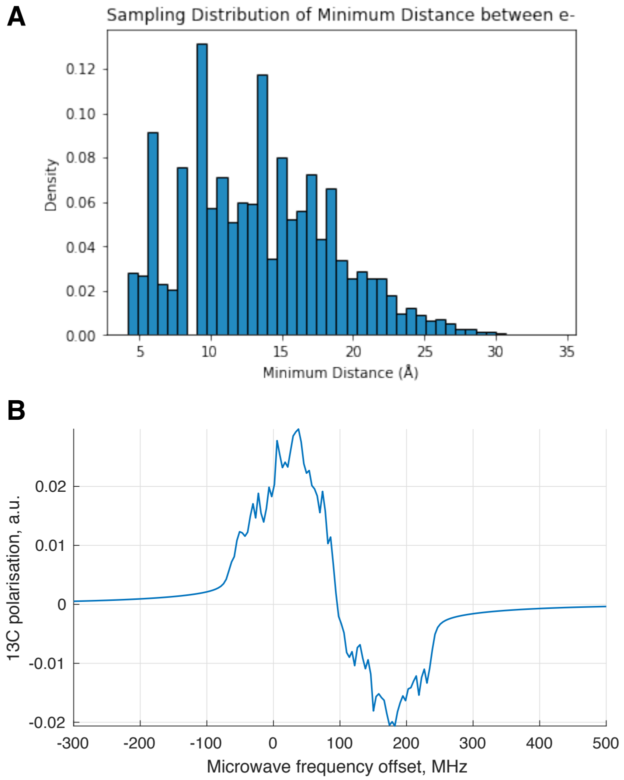

Figure S10: **A**: Simulated minimum electronic-radical distances based on a 70 kGy dose equivalent electron concentration utilising a stochastic damage algorithm in ASE. **B**: Predicted microwave sweep curve from Spinach using atomic coordinates from (**A**) with periodic boundary conditions and mimicking the experimental conditions under which DNP occurred.

6.12 Analytic models

Thermal mixing is a key mechanism in Dynamic Nuclear Polarisation (DNP), widely taken to be dominant in samples used in clinical practice, and theoretically challenging. To quantitatively assess the DNP polarisation behaviour, we implemented a numerical simulation based on Wenckebach's framework in the low temperature limit (*76*).

In this model, the electron spin system is characterised by two parameters: the inverse electron Zeeman temperature $\alpha = \hbar/k_B T_Z$ and the inverse electron non-Zeeman temperature $\beta_{NZ} = \hbar/k_B T_{NZ}$. This assumption splits the electronic states into those that are polarised thermally (Zeeman) and those that will ultimately transfer energy to the nuclei in the irradiation phase of DNP (non-Zeeman). This results in an electron spin polarisation as a function of resonance frequency:

$$P_S(\omega) = \tanh\left[\frac{1}{2}\left(\omega_0\alpha + (\omega - \omega_0)\beta_{NZ}\right)\right] \tag{S11}$$

The evolution of the electron Zeeman and non-Zeeman temperatures is governed by a pair of coupled differential equations:

$$F_1(\omega_m, \alpha, \beta_{NZ}) = -2W(\omega_m)P_s(\omega_m, \alpha, \beta_{NZ}) - \frac{1}{T_1}\left[\int g(\omega)P_s(\omega, \alpha, \beta_{NZ})\mathrm{d}\omega - P_L\right] \tag{S12}$$

$$F_2(\omega_m, \alpha, \beta_{NZ}) = -2W(\omega_m)(\omega_m - \omega_0)P_S(\omega_m, \alpha, \beta_{NZ}) - \frac{1}{T_1}\int g(\omega)(\omega_m - \omega_0)P_s(\omega, \alpha, \beta_{NZ})\,\mathrm{d}\omega \tag{S13}$$

where $g(\omega)$ is the normalised ESR spectral density with centre of mass ω_0, ω_m is the applied microwave frequency, $W(\omega_m) = \frac{1}{2}\pi\omega_{1S}^2 g(\omega_m)$ represents the microwave power, $\omega_{1S} = \gamma_S|B_1|$ is the electron Rabi frequency, T_{1S} is the electron spin-lattice relaxation time, and $P_L = \tanh(\frac{1}{2}\omega_0\beta_L)$ is the thermal equilibrium polarisation with $\beta_L = \hbar/k_B T_L$ being the inverse lattice temperature. High and low temperature limits are defined by the $\omega_0\beta \approx 1$ boundary at roughly around 1 K.

We numerically solved this scheme in Mathematica by use of the explicit analytic calculation of its Jacobian. The function $g(\omega)$ was simulated by EasySpin under appropriate experimental conditions and a numerical interpolation function applied to extend its domain to $[0, \infty]$ (defining it as zero outside of the calculated region).

These equations were evaluated using adaptive Gaussian quadrature (`NIntegrate`) with specified domain constraints. To ensure numerical stability, the working precision was set to 20 digits, with recursion limits extended to handle the complex structure of the integrands. This process took approximately one day on a dual Xeon gold workstation with 768 GB ram, and the simulation results are shown in Fig. S11**A**, with

experimental integrated EPR lineshapes shown in Fig. S11**B**. They produce a bimodal curve qualitatively similar to that in Spinach, and do not reflect experimental results.

As a semi-empirical test with the observed (and not modellable) EPR lineshape obtained, a further simulation was conducted using a semi-empirically scaled EPR lineshape based on that obtained of the alanine/glycerol mixture at 5 K. This simulation was performed in Matlab (providing an independent reimplementation of the model above) and scaled the centre linearly and width (by approximately a factor of two) of the EPR line to be that appropriate at 6.7 T under the assumption that these qualitative features would describe the system, as it is not possible for us to directly measure the EPR spectrum under DNP conditions of 1.4 K and 6.7 T. Both the high temperature and low temperature approximations as defined by Wenckebach have been solved separately and compared to experimental data. Even at 30 kGy irradiation, where a small amount of bimodal behaviour is observed, this model (which accurately reproduces the behaviour of e.g. trityl radicals, TEMPO, Totapol, and other samples) does not fit the data obtained empirically, shown in Fig. S12. We are confident that the bandwidth of the (commercially supplied) microwave excitation source exceeds that shown, and is not the limiting factor in these experiments.

6.13 QuantumESPRESSO

First-principles electronic structure calculations were performed using the plane-wave pseudopotential approach as implemented in the Quantum ESPRESSO (QE) package (version 7.2) (*78, 140*). The Perdew-Burke-Ernzerhof (PBE) generalised gradient approximation (*141*) was used for the exchange-correlation functional. Projector augmented-wave (PAW) pseudopotentials were employed for carbon and oxygen atoms, while ultrasoft pseudopotentials were used for hydrogen and nitrogen atoms, all obtained from the Standard Solid-State Pseudopotential (SSSP) library (efficiency version) (*142*).

The crystal structure of L-alanine was based on the available neutron diffraction data, with a unit cell containing 52 atoms (four alanine molecules). The orthorhombic unit cell parameters were a = 5.7880 Å, b = 6.0360 Å, and c = 12.3420 Å, with space group $P2_12_12_1$ (No. 19).

Calculations were performed with the following parameters: kinetic energy cutoff for wavefunctions of 60 Ry; kinetic energy cutoff for charge density of 480 Ry; 100 Kohn-Sham bands computed to ensure proper description of the conduction bands; electronic convergence threshold of 1.0×10^{-6} Ry; mixing parameter (beta) of 0.6.

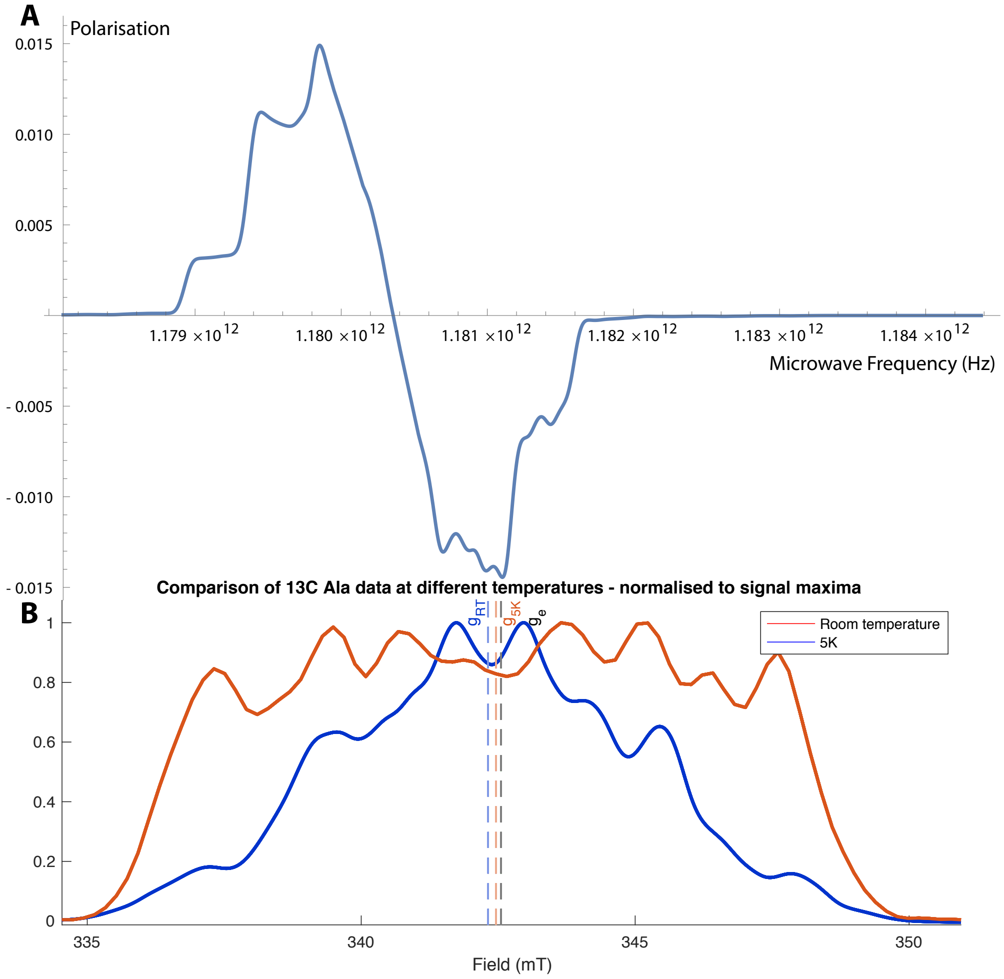

Figure S11: **A**: An analytic model of thermal mixing with the theoretical EPR lineshape given produces a bimodal build up curve. **B**: Integrated experimental low-temperature EPR Spectra obtained for the glycerol/alanine mixture compared to at room temperature (c.f. Fig. 2) indicated a shift in g.

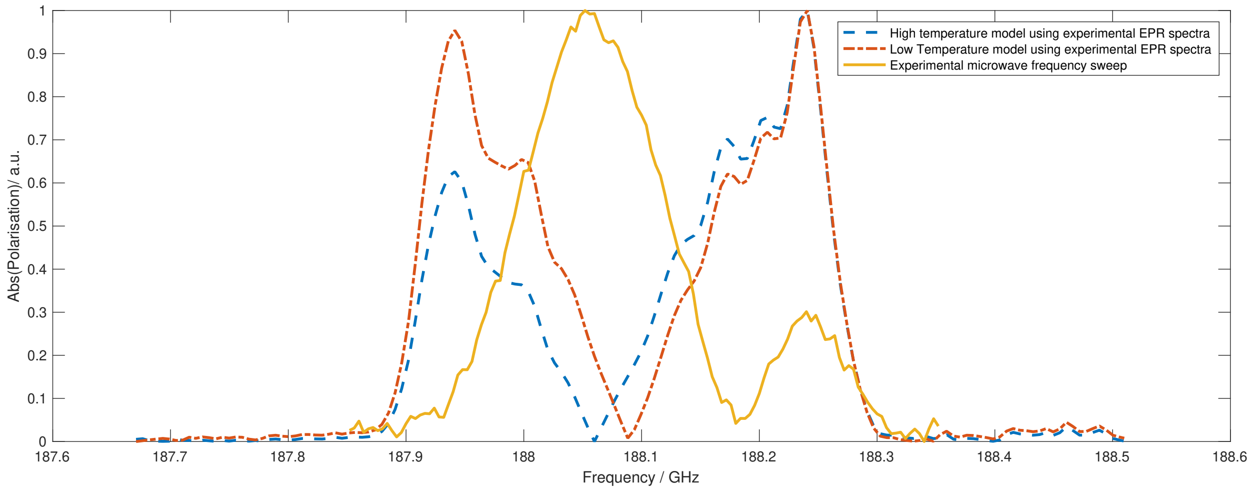

Figure S12: A semi-empirical model of the absolute value of the predicted enhancement curve of DNP as a function of frequency. The EPR spectrum used in this prediction was that obtained at 5 K and lower field, scaled approximately for the system at hand. Whilst some degree of asymmetry is predicted by the Wenckebach model, it is not observed. An empirical shift of 90 MHz aligns the curves, but does not resolve the predicted asymmetry.

Brillouin Zone Sampling

For the band structure calculations, a path along high-symmetry points in the first Brillouin zone was sampled. A total of 392 k-points were used along the path, which included the following segments: $\Gamma \rightarrow$ X; X $\rightarrow$ S; S $\rightarrow$ Y; Y $\rightarrow \Gamma$; $\Gamma \rightarrow$ Z; Z $\rightarrow$ U; U $\rightarrow$ R; R $\rightarrow$ T.

The k-point mesh was generated in crystal coordinates, with a dense sampling to ensure smooth band dispersion.

Computational Resources

The calculations were performed on a parallel computing architecture using 40 MPI processes distributed across a single node and using nearly 1 TB of ram. The electronic structure calculation for the system consumed approximately 24 hours of CPU time and 31 hours of wall time, highlighting the computational intensity of modelling this organic crystal system.

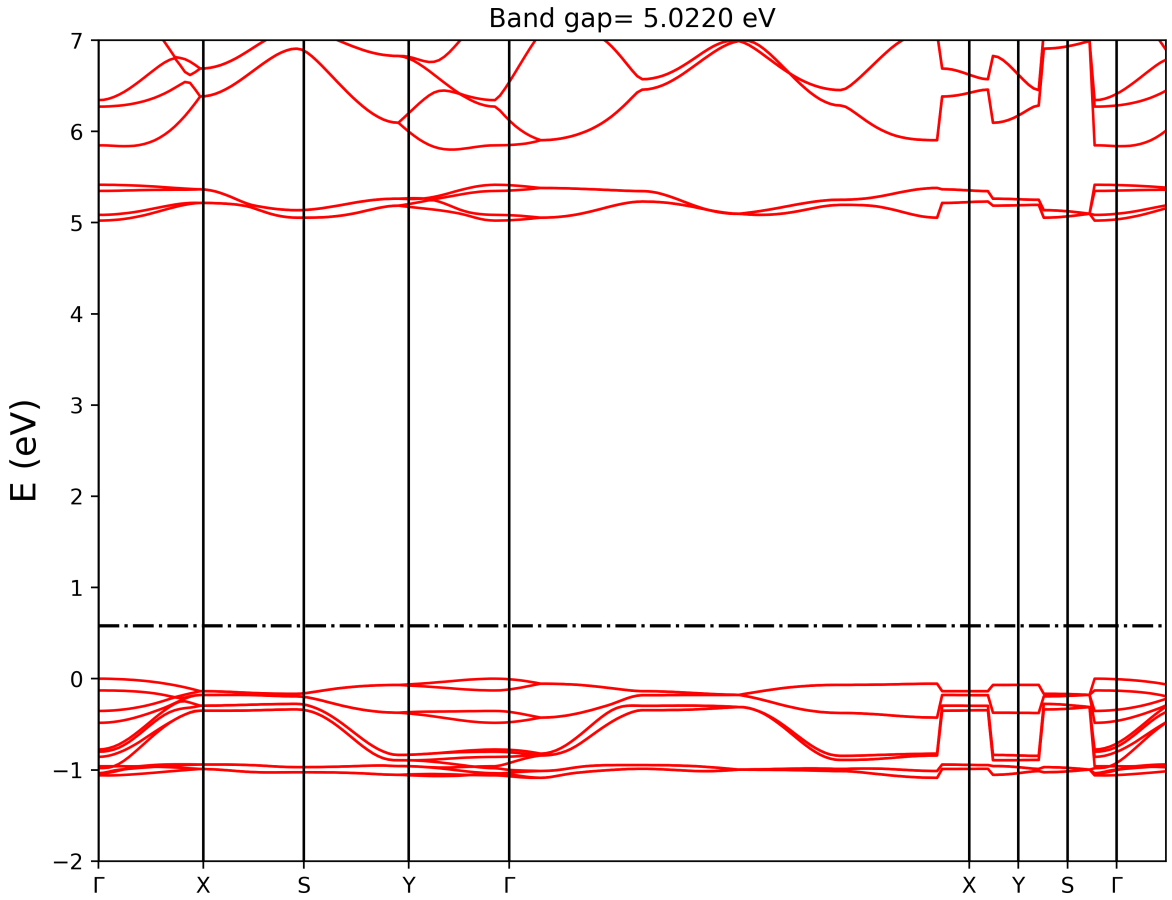

Figure S13: The estimated bandstructure of alanine crystals predicts that it is a relatively large bandgap insulator.

Analysis of Electronic Structure

The band structure calculations revealed a fundamental electronic band gap of approximately 5.02 eV (calculated as the difference between the highest occupied state at 0.58 eV and the lowest unoccupied state at 5.60 eV). The energy eigenvalues were extracted from the full band structure calculation and used to generate the band dispersion plots shown in Fig. S13. This compares favourably to one reported experimental values of 5.4 eV, (*79*) especially as DFT studies using PBE functionals tend to underestimate band gaps by 0.5-1.0 eV.

The total energy of the system was −668.849 Ry, with contributions from: one-electron term (−362.629 Ry); Hartree term (237.420 Ry); exchange-correlation term (−157.484 Ry); Ewald term (−236.071 Ry); one-center PAW contribution (−150.084 Ry).

The relatively large bandgap supports the stability of radical environments, and rules out one (admittedly outlandish) potential mechanism for DNP: that of the well-resolved solid effect.

6.14 Products of dissolution

There were no ^{13}C-labelled impurity peaks present in the dissolution products, and the produced hyperpolarised spectra were largely indistinguishable from that using trityl radicals. This is shown in detail below, in Fig. S14.

7 Data captions

Caption for Data S1. Raw and analysed crystallographic structures for Alanine are provided as Data S1, including structural CIF files and textural fits and refinements used in this work.

Caption for Data S2. Raw EPR data of irradiated and natural abundance alanine used for modelling in this work, together with MATLAB files that reproduce either the modelled spectrum or T_{1e} or T_m fits.

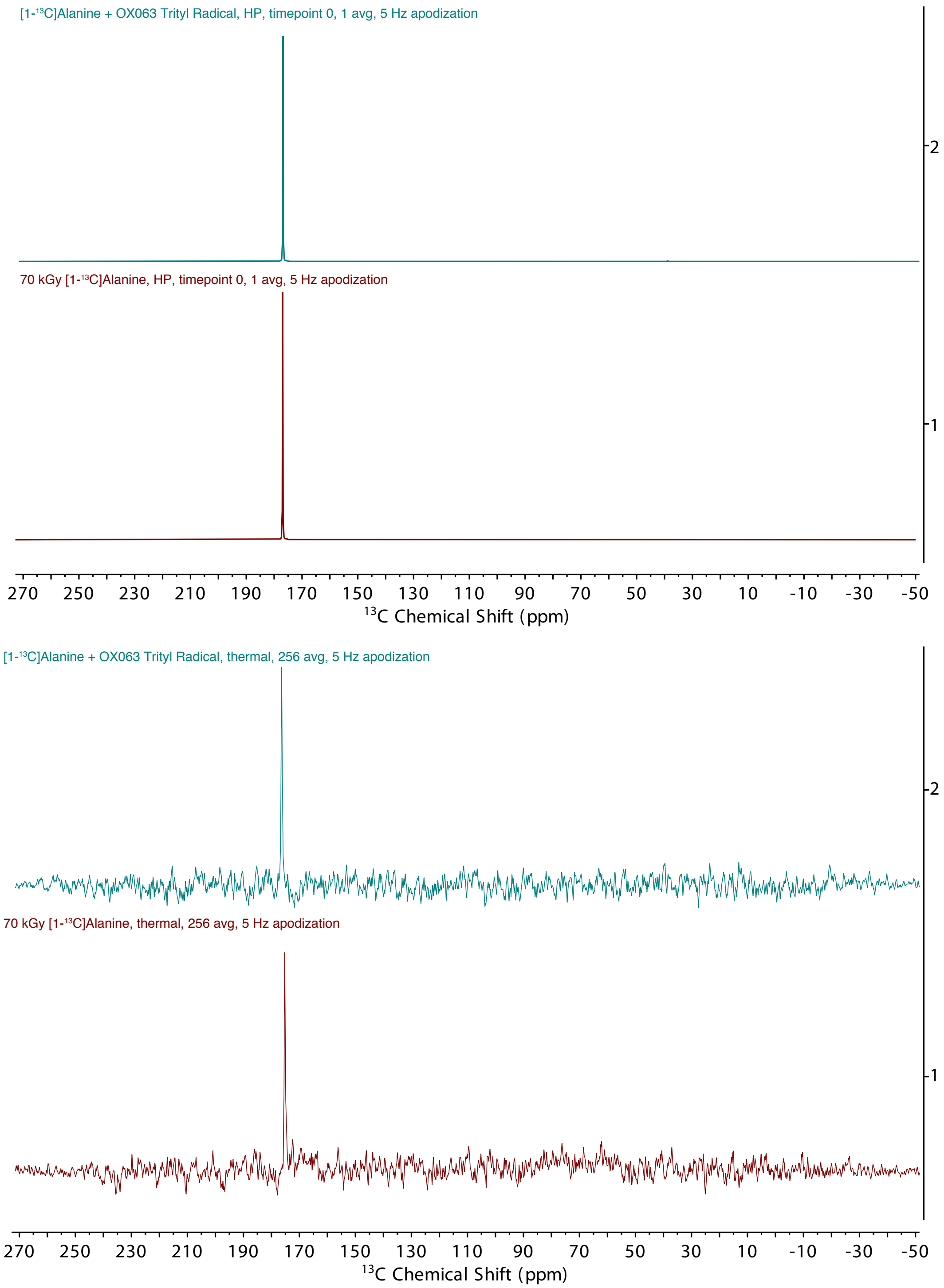

Figure S14: Hyperpolarised alanine produced either with trityl radical or with e-beam irradiation, both when initially dissolved (1 average, WALTS-16 proton decoupling) and corresponding thermal-equilibrium acquisition (256 averages, fully relaxed, WALTZ-16 proton decoupling). No impurity peaks were observed in either spectra. Data obtained at 1.4 T on a Magritek Spinsolve Ultra. The enhancement factor is approximately 142,000.

REFERENCES AND NOTES

1. S. J. Nelson, J. Kurhanewicz, D. B. Vigneron, P. E. Z. Larson, A. L. Harzstark, M. Ferrone, M. van Criekinge, J. W. Chang, R. Bok, I. Park, G. Reed, L. Carvajal, E. J. Small, P. Munster, V. K. Weinberg, J. H. Ardenkjaer-Larsen, A. P. Chen, R. E. Hurd, L.-I. Odegardstuen, F. J. Robb, J. Tropp, J. A. Murray, Metabolic imaging of patients with prostate cancer using hyperpolarized [1-^{13}C]pyruvate. *Sci. Transl. Med.* **5**, 198ra108 (2013).

2. R. Aggarwal, D. B. Vigneron, J. Kurhanewicz, Hyperpolarized 1-[^{13}C]-pyruvate magnetic resonance imaging detects an early metabolic response to androgen ablation therapy in prostate cancer. *Eur. Urol.* **72**, 1028–1029 (2017).

3. M. J. Albers, R. Bok, A. P. Chen, C. H. Cunningham, M. L. Zierhut, V. Y. Zhang, S. J. Kohler, J. Tropp, R. E. Hurd, Y.-F. Yen, S. J. Nelson, D. B. Vigneron, J. Kurhanewicz, Hyperpolarized ^{13}C lactate, pyruvate, and alanine: Noninvasive biomarkers for prostate cancer detection and grading. *Cancer Res.* **68**, 8607–8615 (2008).

4. N. Bahrami, C. L. Swisher, C. Von Morze, D. B. Vigneron, P. E. Z. Larson, Kinetic and perfusion modeling of hyperpolarized ^{13}C pyruvate and urea in cancer with arbitrary RF flip angles. *Quant. Imaging Med. Surg.* **4**, 24–32 (2014).

5. A. P. Chen, M. J. Albers, C. H. Cunningham, S. J. Kohler, Y.-F. Yen, R. E. Hurd, J. Tropp, R. Bok, J. M. Pauly, S. J. Nelson, J. Kurhanewicz, D. B. Vigneron, Hyperpolarized C-13 spectroscopic imaging of the TRAMP mouse at 3T—Initial experience. *Magn. Reson. Med.* **58**, 1099–1106 (2007).

6. R. Faghihi, B. Zeinali-Rafsanjani, M.-A. Mosleh-Shirazi, M. Saeedi-Moghadam, M. Lotfi, R. Jalli, V. Iravani, Magnetic resonance spectroscopy and its clinical applications: A review. *J. Med. Imaging Radiat. Sci.* **48**, 233–253 (2017).

7. K. L. Granlund, S.-S. Tee, H. A. Vargas, S. K. Lyashchenko, E. Reznik, S. Fine, V. Laudone, J. A. Eastham, K. A. Touijer, V. E. Reuter, M. Gonen, R. E. Sosa, D. Nicholson, Y. W. Guo, A. P. Chen, J. Tropp, F. Robb, H. Hricak, K. R. Keshari, Hyperpolarized MRI of human prostate cancer reveals increased lactate with tumor grade driven by monocarboxylate transporter 1. *Cell Metab.* **31**, 105–114.e3 (2020).

8. J. Kurhanewicz, D. B. Vigneron, J. H. Ardenkjaer-Larsen, J. A. Bankson, K. Brindle, C. H. Cunningham, F. A. Gallagher, K. R. Keshari, A. Kjaer, C. Laustsen, D. A. Mankoff, M. E. Merritt, S. J. Nelson, J. M. Pauly, P. Lee, S. Ronen, D. J. Tyler, S. S. Rajan, D. M. Spielman, L. Wald, X. Zhang, C. R. Malloy, R. Rizi, Hyperpolarized ^{13}C MRI: Path to clinical translation in oncology. *Neoplasia* **21**, 1–16 (2019).

9. P. E. Z. Larson, R. Bok, A. B. Kerr, M. Lustig, S. Hu, A. P. Chen, S. J. Nelson, J. M. Pauly, J. Kurhanewicz, D. B. Vigneron, Investigation of tumor hyperpolarized [1-^{13}C]-pyruvate dynamics using time-resolved multiband RF excitation echo-planar MRSI. *Magn. Reson. Med.* **63**, 582–591 (2010).

10. P. E. Z. Larson, A. B. Kerr, C. L. Swisher, J. M. Pauly, D. B. Vigneron, A rapid method for direct detection of metabolic conversion and magnetization exchange with application to hyperpolarized substrates. *J. Magn. Reson.* **225**, 71–80 (2012).

11. P. E. Larson, H.-Y. Chen, J. W. Gordon, N. Korn, J. Maidens, M. Arcak, S. Tang, M. Criekinge, L. Carvajal, D. Mammoli, R. Bok, R. Aggarwal, M. Ferrone, J. B. Slater, S. J. Nelson, J. Kurhanewicz, D. B. Vigneron, Investigation of analysis methods for hyperpolarized ^{13}C-pyruvate metabolic MRI in prostate cancer patients. *NMR Biomed.* **31**, e3997 (2018).

12. F. H. A. van Heijster, S. Heskamp, V. Breukels, A. Veltien, G. M. Franssen, K. C. F. J. Jansen, O. C. Boerman, J. A. Schalken, T. W. J. Scheenen, A. Heerschap, Pyruvate-lactate exchange and glucose uptake in human prostate cancer cell models. A study in xenografts and suspensions by hyperpolarized [1-^{13}C]pyruvate MRS and [^{18}F]FDG-PET. *NMR Biomed.* **33**, e4362 (2020).

13. C. Von Morze, G. Reed, P. Shin, P. E. D. Larson, S. Hu, R. Bok, D. B. Vigneron, Multi-band frequency encoding method for metabolic imaging with hyperpolarized [1-^{13}C]pyruvate. *J. Magn. Reson.* **211**, 109–113 (2011).

14. C. von Morze, S. Sukumar, G. D. Reed, P. E. Z. Larson, R. A. Bok, J. Kurhanewicz, D. B. Vigneron, Frequency-specific SSFP for hyperpolarized ^{13}C metabolic imaging at 14.1 T. *Magn. Reson. Imaging* **31**, 163–170 (2013).

15. E. P. Hackett, M. C. Pinho, C. E. Harrison, G. D. Reed, J. Liticker, J. Raza, R. G. Hall, C. R. Malloy, S. Barshikar, C. J. Madden, J. M. Park, Imaging acute metabolic changes in patients with mild traumatic brain injury using hyperpolarized [1-^{13}C]pyruvate. *iScience* **23**, 101885 (2020).

16. K. L. H. Carpenter, I. Jalloh, C. N. Gallagher, P. Grice, D. J. Howe, A. Mason, I. Timofeev, A. Helmy, M. P. Murphy, D. K. Menon, P. J. Kirkpatrick, T. A. Carpenter, G. R. Sutherland, J. D. Pickard, P. J. Hutchinson, ^{13}C-labelled microdialysis studies of cerebral metabolism in TBI patients. *Eur. J. Pharm. Sci.* **57**, 87–97 (2014).

17. S. J. DeVience, X. Lu, J. Proctor, P. Rangghran, E. R. Melhem, R. Gullapalli, G. M. Fiskum, D. Mayer, Metabolic imaging of energy metabolism in traumatic brain injury using hyperpolarized [1-^{13}C]pyruvate. *Sci. Rep.* **7**, 1907 (2017).

18. C. Guglielmetti, A. Chou, K. Krukowski, C. Najac, X. Feng, L. K. Riparip, S. Rosi, M. M. Chaumeil, In vivo metabolic imaging of traumatic brain injury. *Sci. Rep.* **7**, 17525 (2017).

19. K. Golman, J. S. Petersson, P. Magnusson, E. Johansson, P. Åkeson, C.-M. Chai, G. Hansson, S. Månsson, Cardiac metabolism measured noninvasively by hyperpolarized ^{13}C MRI. *Magn. Reson. Med.* **59**, 1005–1013 (2008).

20. A. J. M. Lewis, J. J. Miller, O. J. Rider, R. P. Choudhury, S. Neubauer, C. A. Carr, D. J. Tyler, C hyperpolarized magnetic resonance imaging of cardiac inflammation and repair. *Heart* **103** (Suppl. 5), A151 (2017).

21. M. A. Schroeder, K. Clarke, S. Neubauer, D. J. Tyler, Hyperpolarized magnetic resonance: A novel technique for the in vivo assessment of cardiovascular disease. *Circulation* **124**, 1580–1594 (2011).

22. C. Ø. Mariager, E. S. S. Hansen, S. K. Bech, H. Eiskjær, P. F. Nielsen, S. Ringgaard, H.-H. Kimose, C. Laustsen, Development of a human heart-sized perfusion system for metabolic imaging studies using hyperpolarized [1-^{13}C]pyruvate MRI. *Magn. Reson. Med.* **85**, 3510–3521 (2021).

23. O. J. Rider, D. J. Tyler, Clinical implications of cardiac hyperpolarized magnetic resonance imaging. *J. Cardiovasc. Magn. Reson.* **15**, 93 (2013).

24. J. J. Miller, J. Lau, D. Tyler, “Hyperpolarized MR in cardiology: Probing the Heart of Life,” in *Advances in Magnetic Resonance Technology and Applications* (Elsevier, 2021), vol. 3, pp. 217–256.

25. M. A. Schroeder, P. Swietach, H. J. Atherton, F. A. Gallagher, P. Lee, G. K. Radda, K. Clarke, D. J. Tyler, Measuring intracellular pH in the heart using hyperpolarized carbon dioxide and bicarbonate: A ^{13}C and ^{31}P magnetic resonance spectroscopy study. *Cardiovasc. Res.* **86**, 82–91 (2010).

26. M. A. Schroeder, A. Z. Lau, A. P. Chen, Y. Gu, J. Nagendran, J. Barry, X. Hu, J. R. B. Dyck, D. J. Tyler, K. Clarke, K. A. Connelly, G. A. Wright, C. H. Cunningham, Hyperpolarized ^{13}C magnetic resonance reveals early- and late-onset changes to in vivo pyruvate metabolism in the failing heart. *Eur. J. Heart Fail* **15**, 130–140 (2013).

27. A. Apps, A. Apps, J. Y. C. Lau, J. J. J. J. Miller, A. Tyler, L. A. J. Young, A. J. M. Lewis, G. Barnes, C. Trumper, S. Neubauer, O. J. Rider, D. J. Tyler, Proof-of-principle demonstration of direct metabolic imaging following myocardial infarction using hyperpolarized ^{13}C CMR. *JACC Cardiovasc. Imaging* **14**, 1285–1288 (2021).

28. S. H. Joergensen, E. S. S. Hansen, N. Bøgh, L. B. Bertelsen, P. B. Staehr, R. F. Schulte, C. Malloy, H. Wiggers, C. Laustsen, Detection of increased pyruvate dehydrogenase flux in the human heart during adenosine stress test using hyperpolarized [1-^{13}C]pyruvate cardiovascular magnetic resonance imaging. *J. Cardiovasc. Magn. Reson.* **24**, 34 (2022).

29. M. Fuetterer, J. Busch, J. Traechtler, P. Wespi, S. M. Peereboom, M. Sauer, M. Lipiski, T. Fleischmann, N. Cesarovic, C. T. Stoeck, S. Kozerke, Quantitative myocardial first-pass cardiovascular magnetic resonance perfusion imaging using hyperpolarized [1-^{13}C] pyruvate. *J. Cardiovasc. Magn. Reson.* **20**, 73 (2018).

30. J. Schwitter, From lab to life: Cardiac biomarker measurement in the intact heart by means of hyperpolarized ^{13}C-carbon CMR. *JACC Cardiovasc. Imaging* **11**, 1607–1610 (2018).

31. P. E. Z. Larson, J. M. L. Bernard, J. A. Bankson, N. Bøgh, R. A. Bok, A. P. Chen, C. H. Cunningham, J. W. Gordon, J.-B. Hövener, C. Laustsen, D. Mayer, M. A. McLean, F. Schilling, J. B. Slater, J.-L. Vanderheyden, C. von Morze, D. B. Vigneron, D. Xu, HP 13C MRI Consensus Group, Current methods for hyperpolarized [1-^{13}C]pyruvate MRI human studies. *Magn. Reson. Med.* **91**, 2204–2228 (2024).

32. J. H. Ardenkjær-Larsen, S. Bowen, J. R. Petersen, O. Rybalko, M. S. Vinding, M. Ullisch, N. C. Nielsen, Cryogen-free dissolution dynamic nuclear polarization polarizer operating at 3.35 T, 6.70 T, and 10.1 T. *Magn. Reson. Med.* **81**, 2184–2194 (2019).

33. A. Capozzi, T. Cheng, G. Boero, C. Roussel, A. Comment, Thermal annihilation of photo-induced radicals following dynamic nuclear polarization to produce transportable frozen hyperpolarized ^{13}C-substrates. *Nat. Commun.* **8**, 15757 (2017).

34. M. Serda, Y.-K. Wu, E. D. Barth, H. J. Halpern, V. H. Rawal, EPR imaging spin probe trityl radical OX063: A method for its isolation from animal effluent, redox chemistry of its quinone methide oxidation product, and in vivo application in a mouse. *Chem. Res. Toxicol.* **29**, 2153–2156 (2016).

35. A. C. Pinon, A. Capozzi, J. H. Ardenkjær-Larsen, Hyperpolarized water through dissolution dynamic nuclear polarization with UV-generated radicals. *Commun. Chem.* **3**, 57 (2020).

36. T. R. Eichhorn, Y. Takado, N. Salameh, A. Capozzi, T. Cheng, J. N. Hyacinthe, M. Mishkovsky, C. Roussel, A. Comment, Hyperpolarization without persistent radicals for in vivo real-time metabolic imaging. *Proc. Natl. Acad. Sci. U.S.A.* **110**, 18064–18069 (2013).

37. A. Giannoulis, K. Butbul, R. Carmieli, J. Kim, E. T. Montrazi, K. Singh, L. Frydman, Cryogenic and dissolution DNP NMR on γ-irradiated organic molecules. *J. Am. Chem. Soc.* **146**, 20758–20769 (2024).

38. A. Apps, "The Development and Application of Novel Magnetic Resonance Spectroscopic and Imaging Techniques to Assess Cardiac Energetics and Substrate Handling in the Human Heart," thesis, University of Oxford (2021).

39. J. A. Urbahn, J. H. Ardenkjær-Larsen, A. M. Leach, E. J. Telfeyan, D. K. Dietrich, K. David, W. D. Brandon, P. Miller, E. W. Stautner, Fluid path system for dissolution and transport of a hyperpolarized material (2014); https://patents.google.com/patent/US8731640B2/en.

40. G. P. Jacobs, Irradiation of pharmaceuticals: A literature review. *Radiat. Phys. Chem.* **190**, 109795 (2022).

41. A. Capozzi, M. Karlsson, Y. Zhao, J. Kilund, Esben Sovso Szocska Hansen, L. B. Bertelsen, C. Laustsen, Jan Henrik Ardenkjaer-Larsen, M. H. Lerche, In-vivo real-time ^{13}C-MRSI without polarizer on site: Across cities transportable hyperpolarization using UV-induced labile radicals. arXiv:2503.18537 [physics.chem-ph] (2025).

42. N. Maltar-Strmečki, B. Rakvin, Thermal stability of radiation-induced free radicals in γ-irradiated L-alanine single crystals. *Applied Radiation and Isotopes* **63**, 375–380 (2005).

43. O. F. Sleptchonok, V. Nagy, M. F. Desrosiers, Advancements in accuracy of the alanine dosimetry system. Part 1. The effects of environmental humidity. *Radiat. Phys. Chem.* **57**, 115–133 (2000).

44. F. C. Chow, M. I. Dysart, D. W. Hamar, L. D. Lewis, R. H. Udall, Alanine: A toxicity study. *Toxicol. Appl. Pharmacol.* **37**, 491–497 (1976).

45. W. W. Tourtellotte, J. L. Reinglass, T. A. Newkirk, Cerebral dehydration action of glycerol; I. Historical aspects with emphasis on the toxicity and intravenous administration. *Clin. Pharmacol. Ther.* **13**, 159–171 (1972).

46. H. Jóhannesson, S. Macholl, J. H. Ardenkjaer-Larsen, Dynamic nuclear polarization of [1-^{13}C]pyruvic acid at 4.6 tesla. *J. Magn. Reson.* **197**, 167–175 (2009).

47. M.-C. Vozenin, J. Bourhis, M. Durante, Towards clinical translation of FLASH radiotherapy. *Nat. Rev. Clin. Oncol.* **19**, 791–803 (2022).

48. A. Berne, K. Petersson, I. D. C. Tullis, R. G. Newman, B. Vojnovic, Monitoring electron energies during FLASH irradiations. *Phys. Med. Biol.* **66**, 045015 (2021).

49. B. Vojnovic, I. D. C. Tullis, R. G. Newman, K. Petersson, Monitoring beam charge during FLASH irradiations. *Front. Phys.* **11**, 1185237 (2023).

50. M. Z. Heydari, E. Malinen, E. O. Hole, E. Sagstuen, Alanine radicals. 2. The composite polycrystalline alanine EPR spectrum studied by ENDOR, thermal annealing, and spectrum simulations. *J. Phys. Chem. A* **106**, 8971–8977 (2002).

51. M. Janbazi, Y. T. Azar, F. Ziaie, EPR parameters of L-α-alanine radicals in aqueous solution: A first-principles study. *Mol. Phys.* **116**, 1795–1803 (2018).

52. E. O. Jåstad, T. Torheim, K. M. Villeneuve, K. Kvaal, E. O. Hole, E. Sagstuen, E. Malinen, C. M. Futsaether, In quest of the alanine R3 radical: Multivariate EPR spectral analyses of X-irradiated alanine in the solid state. *J. Phys. Chem. A* **121**, 7139–7147 (2017).

53. E. Malinen, M. Z. Heydari, E. Sagstuen, E. O. Hole, Alanine radicals, Part 3: Properties of the components contributing to the EPR spectrum of X-irradiated alanine dosimeters. *Radiat. Res.* **159**, 23–32 (2003).

54. E. Pauwels, H. D. Cooman, M. Waroquier, E. O. Hole, E. Sagstuen, Solved? The reductive radiation chemistry of alanine. *Phys. Chem. Chem. Phys.* **16**, 2475–2482 (2014).

55. E. Sagstuen, E. O. Hole, S. R. Haugedal, W. H. Nelson, Alanine radicals: Structure determination by EPR and ENDOR of single crystals X-irradiated at 295 K. *J. Phys. Chem. A* **101**, 9763–9772 (1997).

56. K. Matsuki, I. Miyagawa, ENDOR study of an irradiated crystal of *L*-alanine: Structure and the environment of the unstable $CH_3\dot{C}HCO_2^-$ radical. *J. Chem. Phys.* **76**, 3945–3952 (1982).

57. I. Miyagawa, W. Gordy, Electron spin resonance of an irradiated single crystal of alanine: Second-order effects in free radical resonances. *J. Chem. Phys.* **32**, 255–263 (1960).

58. S.-i. Kuroda, I. Miyagawa, ENDOR study of an irradiated crystal of *L*-alanine: Environment of the stable $CH_3\ \dot{C}HCO_2^-$ radical. *J. Chem. Phys.* **76**, 3933–3944 (1982).

59. P. Lahorte, F. de Proft, G. Vanhaelewyn, B. Masschaele, P. Cauwels, F. Callens, P. Geerlings, W. Mondelaers, Density functional calculations of hyperfine coupling constants in alanine-derived radicals. *J. Phys. Chem. A* **103**, 6650–6657 (1999).

60. R. Martinez-Cantin, BayesOpt: A Bayesian optimization library for nonlinear optimization, experimental design and bandits. *J. Mach. Learn. Res.* **15**, 3735–3739 (2014).

61. J. Sinclair, M. W. Hanna, ESR study of L-alanine-1-^{13}C irradiated at low temperatures. *J. Chem. Phys.* **50**, 2125–2129 (1969).

62. A. Capozzi, S. Patel, W. T. Wenckebach, M. Karlsson, M. H. Lerche, J. H. Ardenkjær-Larsen, Gadolinium effect at high-magnetic-field DNP: 70% ^{13}C polarization of [U-^{13}C] glucose using trityl. *J. Phys. Chem. Lett.* **10**, 3420–3425 (2019).

63. L. M. Epasto, T. Maimbourg, A. Rosso, D. Kurzbach, Unified understanding of the breakdown of thermal mixing dynamic nuclear polarization: The role of temperature and radical concentration. *J. Magn. Reson.* **362**, 107670 (2024).

64. W. T. Wenckebach, Electron spin–spin interactions in DNP: Thermal mixing vs. the cross effect. *Appl. Magn. Reson.* **52**, 731–748 (2021).

65. Y. Hovav, A. Feintuch, S. Vega, Dynamic nuclear polarization assisted spin diffusion for the solid effect case. *J. Chem. Phys.* **134**, 074509 (2011).

66. B. A. Rodin, V. Thalakottoor, M. Baudin, N. Birilirakis, G. Bodenhausen, A. V. Yurkovskaya, D. Abergel, Quantitative analysis of cross-talk in partly deuterated samples of nuclear spins hyperpolarized by dynamic nuclear polarization (DNP) in the thermal mixing regime. *Phys. Chem. Chem. Phys.* **25**, 15040–15051 (2023).

67. L. B. McCusker, R. B. Von Dreele, D. E. Cox, D. Louër, P. Scardi, Rietveld refinement guidelines. *J. Appl. Crystallogr.* **32**, 36–50 (1999).

68. L. Lutterotti, H. Wenk, S. Matthies, MAUD (Material Analysis Using Diffraction): A User Friendly Java Program for Rietveld Texture Analysis and More, in *Proceeding of the Twelfth*

International Conference on Textures of Materials (ICOTOM-12) (NRC Research Press, vol. 2, 1999), pp. 1599–1604; https://iris.unitn.it/handle/11572/57067.

69. D. A. Keen, M. J. Gutmann, C. C. Wilson, SXD–the single-crystal diffractometer at the ISIS Spallation Neutron Source. *J. Appl. Crystallogr.* **39**, 714–722 (2006).

70. C. C. Wilson, D. Myles, M. Ghosh, L. N. Johnson, W. Wang, Neutron diffraction investigations of L- and D-alanine at different temperatures: The search for structural evidence for parity violation. *New J. Chem.* **29**, 1318 (2005).

71. A. P. Thompson, H. M. Aktulga, R. Berger, D. S. Bolintineanu, W. M. Brown, P. S. Crozier, P. J. in 't Veld, A. Kohlmeyer, S. G. Moore, T. D. Nguyen, R. Shan, M. J. Stevens, J. Tranchida, C. Trott, S. J. Plimpton, LAMMPS—A flexible simulation tool for particle-based materials modeling at the atomic, meso, and continuum scales. *Comput. Phys. Commun.* **271**, 108171 (2022).

72. P. J. In 'T Veld, G. C. Rutledge, Temperature-dependent elasticity of a semicrystalline interphase composed of freely rotating chains. *Macromolecules* **36**, 7358–7365 (2003).

73. H. J. Hogben, M. Krzystyniak, G. T. Charnock, P. J. Hore, I. Kuprov, *Spinach*–A software library for simulation of spin dynamics in large spin systems. *J. Magn. Reson.* **208**, 179–194 (2011).

74. H. Kruse, S. Grimme, A geometrical correction for the inter- and intra-molecular basis set superposition error in Hartree-Fock and density functional theory calculations for large systems. *J. Chem. Phys.* **136**, 154101 (2012).

75. A. Richards, Technical Note on ARC facility and service deployment for publication reference, University of Oxford Advanced Research Computing (2015); https://doi.org/10.5281/zenodo.22558.

76. W. T. Wenckebach, Dynamic nuclear polarization via thermal mixing: Beyond the high temperature approximation. *J. Magn. Reson.* **277**, 68–78 (2017).

77. W. T. Wenckebach, Dynamic nuclear polarization via the cross effect and thermal mixing: A. The role of triple spin flips. *J. Magn. Reson.* **299**, 124–134 (2019).

78. P. Giannozzi, S. Baroni, N. Bonini, M. Calandra, R. Car, C. Cavazzoni, D. Ceresoli, G. L. Chiarotti, M. Cococcioni, I. Dabo, A. D. Corso, S. de Gironcoli, S. Fabris, G. Fratesi, R. Gebauer, U. Gerstmann, C. Gougoussis, A. Kokalj, M. Lazzeri, L. Martin-Samos, N. Marzari, F. Mauri, R. Mazzarello, S. Paolini, A. Pasquarello, L. Paulatto, C. Sbraccia, S. Scandolo, G. Sclauzero, A. P. Seitsonen, A. Smogunov, P. Umari, R. M. Wentzcovitch, QUANTUM ESPRESSO: A modular and open-source software project for quantumsimulations of materials. *J. Phys. Condens. Matter* **21**, 395502 (2009).

79. N. Suresh, M. Selvapandiyan, Influence of zirconium nitrate doping on the properties of L-alanine crystal for nonlinear optical applications. *J. Mater. Sci. Mater. Electron.* **31**, 16737–16745 (2020).

80. A. Abragam, M. Goldman, Principles of dynamic nuclear polarisation. *Rep. Prog. Phys.* **41**, 395 (1978).

81. G. L. Hug, I. Carmichael, R. W. Fessenden, Direct EPR observation of the aminomethyl radical during the radiolysis of glycine. *J. Chem. Soc., Perkin Trans.* **5**, 907–908 (2000).

82. P. M. Nielsen, C. Ø. Mariager, M. Mølmer, N. Sparding, F. Genovese, M. A. Karsdal, R. Nørregaard, L. B. Bertelsen, C. Laustsen, Hyperpolarized [1–^{13}C] alanine production: A novel imaging biomarker of renal fibrosis. *Magn. Reson. Med.* **84**, 2063–2073 (2020).

83. K. Hansen, E. S. S. Hansen, N. R. V. Jespersen, H. E. Bøtker, M. Pedersen, T. Wang, C. Laustsen, Hyperpolarized ^{13}C MRI reveals large changes in pyruvate metabolism during digestion in snakes. *Magn. Reson. Med.* **88**, 890–900 (2022).

84. S. Hu, M. Zhu, H. A. I. Yoshihara, D. M. Wilson, K. R. Keshari, P. Shin, G. Reed, C. von Morze, R. Bok, P. E. Z. Larson, J. Kurhanewicz, D. B. Vigneron, In vivo measurement of normal rat intracellular pyruvate and lactate levels after injection of hyperpolarized [1–^{13}C] alanine. *Magn. Reson. Imaging* **29**, 1035–1040 (2011).

85. C. N. Wiens, L. J. Friesen-Waldner, T. P. Wade, K. J. Sinclair, C. A. McKenzie, Chemical shift encoded imaging of hyperpolarized ^{13}C pyruvate. *Magn. Reson. Med.* **74**, 1682–1689 (2015).

86. A. Dölle, Metabolism of D- and L- [^{13}C]alanine in rat liver detected by ^{1}H and ^{13}C NMR spectroscopy in vivo and in vitro. *NMR Biomed.* **13**, 72–81 (2000).

87. J. H. Ardenkjaer-Larsen, B. Fridlund, A. Gram, G. Hansson, L. Hansson, M. H. Lerche, R. Servin, M. Thaning, K. Golman, Increase in signal-to-noise ratio of > 10,000 times in liquid-state NMR. *Proc. Natl. Acad. Sci. U.S.A.* **100**, 10158–10163 (2003).

88. A. Apps, J. Lau, M. Peterzan, S. Neubauer, D. Tyler, O. Rider, Hyperpolarised magnetic resonance for in vivo real-time metabolic imaging. *Heart* **104**, 1484–1491 (2018).

89. O. J. Rider, A. Apps, J. J. J. J. Miller, J. Y. C. Lau, A. J. M. Lewis, M. A. Peterzan, M. S. Dodd, A. Z. Lau, C. Trumper, F. A. Gallagher, J. T. Grist, K. M. Brindle, S. Neubauer, D. J. Tyler, Noninvasive in vivo assessment of cardiac metabolism in the healthy and diabetic human heart using hyperpolarized ^{13}C MRI. *Circ. Res.* **126**, 725–736 (2020).

90. P. Larson, S. Sahin, E. Milshteyn, X. Liu, J. Gordon, korench1, X. Zhu, J. Maidens, acatbennett, hychen88, N. V. Christensen, J. Bernard, H. Shang, LarsonLab/Hyperpolarized-Mri-Toolbox: Updated READMEs, Visualization Tools and bSSFP Fitting (2024); doi:10.5281/zenodo.10915690.

91. J. H. Ardenkjaer-Larsen, A. M. Leach, N. Clarke, J. Urbahn, D. Anderson, T. W. Skloss, Dynamic nuclear polarization polarizer for sterile use intent. *NMR Biomed.* **24**, 927–932 (2011).

92. J. H. Ardenkjær-Larsen, I. Laursen, I. Leunbach, G. Ehnholm, L. G. Wistrand, J. S. Petersson, K. Golman, EPR and DNP properties of certain novel single electron contrast agents intended for oximetric imaging. *J. Magn. Reson.* **133**, 1–12 (1998).

93. X. Ji, A. Bornet, B. Vuichoud, J. Milani, D. Gajan, A. J. Rossini, L. Emsley, G. Bodenhausen, S. Jannin, Transportable hyperpolarized metabolites. *Nat. Commun.* **8**, 13975 (2017).

94. A. S. Kiryutin, B. A. Rodin, A. V. Yurkovskaya, K. L. Ivanov, D. Kurzbach, S. Jannin, D. Guarin, D. Abergel, G. Bodenhausen, Transport of hyperpolarized samples in dissolution-DNP experiments. *Phys. Chem. Chem. Phys.* **21**, 13696–13705 (2019).

95. H. Gutte, A. E. Hansen, M. M. E. Larsen, S. Rahbek, S. T. Henriksen, H. H. Johannesen, J. Ardenkjaer-Larsen, A. T. Kristensen, L. Højgaard, A. Kjær, Simultaneous hyperpolarized ^{13}C-pyruvate MRI and ^{18}F-FDG PET (HyperPET) in 10 dogs with cancer. *J. Nucl. Med.* **56**, 1786–1792 (2015).

96. W. W. Bradshaw, D. G. Cadena, G. W. Crawford, H. A. W. Spetzler, The use of alanine as a solid dosimeter. *Radiat. Res.* **17**, 11–21 (1962).

97. DIN Deutsches Institut für Normung e.V., DIN-Normenausschuss Gesundheitstechnologien, DIN EN ISO 11137-2: 2023-08: Sterilization of Health Care Products-Radiation. Pt. 2: Establishing the Sterilization Dose (ISO 11137-2: 2013 + Amd 1: 2022); German Version EN ISO 11137-2: 2015 + A1: 2023 (2025); https://inis.iaea.org/records/6c4pf-h4d55.

98. E. Hoxey, "Medical Device White Paper Series on Sterilization—Regulatory Requirements and Supporting Standards" (BSI National Standards Body, 2020); https://sfsap.org/wp-content/uploads/2020/07/Sterilization_Revised1.pdf.

99. J. M. Park, C. Khemtong, S.-C. Liu, R. E. Hurd, D. M. Spielman, In vivo assessment of intracellular redox state in rat liver using hyperpolarized [1–^{13}C]alanine. *Magn. Reson. Med.* **77**, 1741–1748 (2017).

100. A. Radaelli, R. Gruetter, H. A. I. Yoshihara, In vivo detection of D-amino acid oxidase with hyperpolarized D-[1–^{13}C]alanine. *NMR Biomed.* **33**, e4303 (2020).

101. P. Viswanath, G. Batsios, J. Mukherjee, A. M. Gillespie, P. E. Z. Larson, H. A. Luchman, J. J. Phillips, J. F. Costello, R. O. Pieper, S. M. Ronen, Non-invasive assessment of telomere maintenance mechanisms in brain tumors. *Nat. Commun.* **12**, 92 (2021).

102. K. Matsumoto, F. Kimura, S. Tsukui, T. Kimura, X-ray diffraction of a magnetically oriented microcrystal suspension of L-alanine. *Cryst. Growth Des.* **11**, 945–948 (2011).

103. W. Wang, X. Sheng, H. Jin, J. Wu, B. Yin, J. Li, Z. Zhao, H. Yang, F. Lou, Z. Zhuang, G. Yu, L. Shi, Z. Chen, Susceptibility behaviour and specific heat anomaly in single crystals of alanine and valine. *J. Biol. Phys.* **22**, 65–71 (1996).

104. W. Wang, W. Min, F. Bai, L. Sun, F. Yi, Z. Wang, C. Yan, Y. Ni, Z. Zhao, Temperature-dependent magnetic susceptibilities study on parity-violating phase transition of D- and L-alanine crystals. *Tetrahedron Asymmetr.* **13**, 2427–2432 (2002).

105. W.-Q. Wang, X.-C. Shen, Y. Gong, Cryogenic magnetic transition of D- and L-alanine: Magnetic field dependence of specific heat and DC magnetic susceptibility. *Acta Phys. Chim. Sin.* **26**, 2597–2603 (2010).

106. A. Boulle, A. Chartier, J. P. Crocombette, T. Jourdan, S. Pellegrino, A. Debelle, Strain and damage build-up in irradiated crystals: Coupling x-ray diffraction with numerical simulations. *Nucl. Instrum. Methods Phys. Res., B* **458**, 143–150 (2019).

107. C. J. M. Monagle, C. A. Fuller, E. Hupf, L. A. Malaspina, S. Grabowsky, D. Chernyshov, A. Expand, Lattice response to the radiation damage of molecular crystals: Radiation-induced versus thermal expansivity. *Acta Crystallogr. B Struct. Sci. Cryst. Eng. Mater* **80**, 13–18 (2024).

108. B. D. Wirth, How does radiation damage materials? *Science* **318**, 923–924 (2007).

109. C. H. E. Rooney, A. Gamliel, D. Shaul, D. J. Tyler, J. T. Grist, R. Katz-Brull, Directly bound deuterons increase X-nuclei hyperpolarization using dynamic nuclear polarization. *ChemPhysChem* **24**, e202300144 (2023).

110. C. C. Zanella, A. Capozzi, H. A. I. Yoshihara, A. Radaelli, A. L. C. Mackowiak, L. P. Arn, R. Gruetter, J. A. M. Bastiaansen, Radical-free hyperpolarized MRI using endogenously occurring pyruvate analogues and UV-induced nonpersistent radicals. *NMR Biomed.* **34**, e4584 (2021).

111. N. W. Lutz, Y. L. Fur, J. Chiche, J. Pouysse, P. J. Cozzone, Quantitative in vivo characterization of intracellular and extracellular pH profiles in heterogeneous tumors: A novel method enabling multiparametric pH analysis. *Cancer Res.* **73**, 4616–4628 (2013).

112. A. Z. Lau, A. P. Chen, N. R. Ghugre, V. Ramanan, W. W. Lam, K. A. Connelly, G. A. Wright, C. H. Cunningham, Rapid multislice imaging of hyperpolarized ^{13}C pyruvate and bicarbonate in the heart. *Magn. Reson. Med.* **64**, 1323–1331 (2010).

113. A. S. Lilly Thankamony, J. J. Wittmann, M. Kaushik, B. Corzilius, Dynamic nuclear polarization for sensitivity enhancement in modern solid-state NMR. *Prog. Nucl. Magn. Reson. Spectrosc.* **102-103**, 120–195 (2017).

114. IAEA, "Radiation Effects on Polymer Materials Commonly Used in Medical Devices" (CRP Report of the 1st RCM for CRP F23035, 2021); https://nucleus.iaea.org/sites/diif/PublishingImages/Pages/IAEAPublications/IAEA%201st%20RCM%20F23035%20Report_Final.pdf.

115. I. I. White, G. Von, R. Tandon, L. M. Serna, M. C. Celina, R. Bernstein, "An Overview of Basic Radiation Effects on Polymers and Glasses" (Tech. Rep. Sandia National Laboratory, 2013); https://www.osti.gov/servlets/purl/1671997.

116. H.-Y. Chen, P. E. Z. Larson, J. W. Gordon, R. A. Bok, M. Ferrone, M. van Criekinge, L. Carvajal, P. Cao, J. M. Pauly, A. B. Kerr, I. Park, J. B. Slater, S. J. Nelson, P. N. Munster, R. Aggarwal, J. Kurhanewicz, D. B. Vigneron, Technique development of 3D dynamic CS-EPSI for hyperpolarized ^{13}C pyruvate MR molecular imaging of human prostate cancer. *Magn. Reson. Med.* **80**, 2062–2072 (2018).

117. O. Burghaus, M. Rohrer, T. Gotzinger, M. Plato, K. Mobius, A novel high-field/high-frequency EPR and ENDOR spectrometer operating at 3 mm wavelength. *Meas. Sci. Technol.* **3**, 765 (1992).

118. J. Henning, J. Den Boef, High resolution ESR spectrum of cubic Cr^{3+} in MgO. *Phys. Lett. A* **59**, 241–242 (1976).

119. S. Stoll, A. Schweiger, EasySpin, a comprehensive software package for spectral simulation and analysis in EPR. *J. Magn. Reson.* **178**, 42–55 (2006).

120. B. Faddegon, J. Ramos-Méndez, J. Schuemann, A. McNamara, J. Shin, J. Perl, H. Paganetti, The TOPAS Tool for Particle Simulation, a Monte Carlo simulation tool for physics, biology and clinical research. *Phys. Medica* **72**, 114–121 (2020).

121. J. Perl, J. Shin, J. Schümann, B. Faddegon, H. Paganetti, TOPAS: An innovative proton Monte Carlo platform for research and clinical applications. *Med. Phys.* **39**, 6818–6837 (2012).

122. S. Agostinelli, J. Allison, K. Amako, J. Apostolakis, H. Araujo, P. Arce, M. Asai, D. Axen, S. Banerjee, G. Barrand, F. Behner, L. Bellagamba, J. Boudreau, L. Broglia, A. Brunengo, H. Burkhardt, S. Chauvie, J. Chuma, R. Chytracek, G. Cooperman, G. Cosmo, P. Degtyarenko, A. Dell'Acqua, G. Depaola, D. Dietrich, R. Enami, A. Feliciello, C. Ferguson, H. Fesefeldt, G. Folger, F. Foppiano, A. Forti, S. Garelli, S. Giani, R. Giannitrapani, D. Gibin, J. J. Gómez Cadenas, I. González, G. Gracia Abril, G. Greeniaus, W. Greiner, V. Grichine, A. Grossheim, S. Guatelli, P. Gumplinger, R. Hamatsu, K. Hashimoto, H. Hasui, A. Heikkinen, A. Howard, V. Ivanchenko, A. Johnson, F. W. Jones, J. Kallenbach, N. Kanaya, M. Kawabata, Y. Kawabata, M. Kawaguti, S. Kelner, P. Kent, A. Kimura, T. Kodama, R. Kokoulin, M. Kossov, H. Kurashige, E. Lamanna, T. Lampén, V. Lara, V. Lefebure, F. Lei, M. Liendl, W. Lockman, F. Longo, S. Magni, M. Maire, E. Medernach, K. Minamimoto, P. Mora de Freitas, Y. Morita, K. Murakami, M. Nagamatu, R. Nartallo, P. Nieminen, T. Nishimura, K. Ohtsubo, M. Okamura, S. O'Neale, Y. Oohata, K. Paech, J. Perl, A. Pfeiffer, M. G. Pia, F. Ranjard, A. Rybin, S. Sadilov, E. di Salvo, G. Santin, T. Sasaki, N. Savvas, Y. Sawada, S. Scherer, S. Sei, V. Sirotenko, D. Smith, N. Starkov, H. Stoecker, J. Sulkimo, M. Takahata, S. Tanaka, E. Tcherniaev, E. Safai Tehrani, M. Tropeano, P. Truscott, H. Uno, L. Urban, P. Urban, M. Verderi, A. Walkden, W. Wander, H. Weber, J. P. Wellisch, T. Wenaus, D. C. Williams, D. Wright, T. Yamada, H. Yoshida, D. Zschiesche, GEANT4—A simulation toolkit. *Nucl. Instrum.Methods Phys. Res. A* **506**, 250–303 (2003).

123. K. O. Tan, C. Yang, R. T. Weber, G. Mathies, R. G. Griffin, Time-optimized pulsed dynamic nuclear polarization. *Sci. Adv.* **5**, eaav6909 (2019).

124. T. V. Can, J. J. Walish, T. M. Swager, R. G. Griffin, Time domain DNP with the NOVEL sequence. *J. Chem. Phys.* **143**, 054201 (2015).

125. A. Javed, M. Y. Ghazi, V. SubbaRao Redrouthu, A. Equbal, Magic-NOVEL: Suppressing electron–electron coupling effects in pulsed DNP. *J. Chem. Phys.* **162**, 014202 (2025).

126. G. R. Eaton, S. S. Eaton, D. P. Barr, R. T. Weber, *Quantitative EPR* (Springer Science & Business Media, 2010); https://books.google.com/books?hl=en&lr=&&id=sayWdlbWGfwC&oi=fnd&pg=PA1&dq=best+practices+quantitative+EPR&ots=h7wrEVOshI&sig=B3x8LKkLkziEq7H2umcCOF6EhcY.

127. M. Mazur, A dozen useful tips on how to minimise the influence of sources of error in quantitative electron paramagnetic resonance (EPR) spectroscopy—A review. *Anal. Chim. Acta* **561**, 1–15 (2006).

128. P. Barreau, M. Bernheim, J. Duclos, J. M. Finn, Z. Meziani, J. Morgenstern, J. Mougey, D. Royer, B. Saghai, D. Tarnowski, S. Turck-Chieze, M. Brussel, G. P. Capitani, E. de Sanctis, S. Frullani, F. Garibaldi, D. B. Isabelle, E. Jans, I. Sick, P. D. Zimmerman, Deep-inelastic electron scattering from carbon. *Nucl. Phys. A* **402**, 515–540 (1983).

129. I.-T. Cheon, Electron scattering from ^{13}C. *Phys. Lett. B* **124**, 451–457 (1983).

130. M. Deady, C. F. Williamson, P. D. Zimmerman, R. Altemus, R. R. Whitney, Deep inelastic separated response functions from ^{40}Ca and ^{48}Ca. *Phys. Rev. C* **33**, 1897–1904 (1986).

131. J. Heisenberg, J. McCarthy, I. Sick, Elastic electron scattering from ^{13}C. *Nucl. Phys. A* **157**, 435–448 (1970).

132. M. Berger, J. Coursey, M. Zucker, ESTAR, PSTAR, and ASTAR: Computer Programs for Calculating Stopping-Power and Range Tables for Electrons, Protons, and Helium Ions (Version 1.21) (1999).

133. H. Sun, COMPASS: An ab initio force-field optimized for condensed-phase applicationsoverview with details on alkane and benzene compounds. *J. Phys. Chem. B* **102**, 7338–7364 (1998).

134. H. Sun, P. Ren, J. R. Fried, The COMPASS force field: Parameterization and validation for phosphazenes. *Comput. Theor. Polym. Sci.* **8**, 229–246 (1998).

135. S. Meng, W. Li, X. Yin, J. Xie, A comprehensive theoretical study of the hydrogen bonding interactions and microscopic solvation structures of a pyridyl-urea-based hydrogelator in aqueous solution. *Comput. Theor. Chem.* **1006**, 76–84 (2013).

136. J. Sun, H. Zhang, K. Guo, S. Yuan, Self-assembly of dipeptide sodium salts derived from alanine: A molecular dynamics study. *RSC Adv.* **5**, 102182–102190 (2015).

137. X. Yang, G. Qian, X. Duan, X. Zhou, Impurity effect of L-valine on L-alanine crystal growth. *Cryst. Growth Des.* **13**, 1295–1300 (2013).

138. W. Humphrey, A. Dalke, K. Schulten, VMD: Visual Molecular Dynamics. *Journal of molecular graphics* **14**, 33–38 (1996).

139. A. Hjorth Larsen, J. J. Mortensen, J. Blomqvist, I. E. Castelli, R. Christensen, M. Dułak, J. Friis, M. N. Groves, B. Hammer, C. Hargus, E. D. Hermes, P. C. Jennings, P. B. Jensen, J. Kermode, J. R. Kitchin, E. L. Kolsbjerg, J. Kubal, K. Kaasbjerg, S. Lysgaard, J. Bergmann Maronsson, T. Maxson, T. Olsen, L. Pastewka, A. Peterson, C. Rostgaard, J. Schiøtz, O. Schütt, M. Strange, K. S. Thygesen, T. Vegge, L. Vilhelmsen, M. Walter, Z. Zeng, K. W. Jacobsen, The atomic simulation environment—A Python library for working with atoms. *J. Phys. Condens. Matter* **29**, 273002 (2017).

140. P. Giannozzi, O. Andreussi, T. Brumme, O. Bunau, M. B. Nardelli, M. Calandra, R. Car, C. Cavazzoni, D. Ceresoli, M. Cococcioni, N. Colonna, I. Carnimeo, A. D. Corso, S. de Gironcoli, P. Delugas, R. A. Di Stasio Jr., A. Ferretti, A. Floris, G. Fratesi, G. Fugallo, R. Gebauer, U. Gerstmann, F. Giustino, T. Gorni, J. Jia, M. Kawamura, H.-Y. Ko, A. Kokalj, E. Küçükbenli, M. Lazzeri, M. Marsili, N. Marzari, F. Mauri, N. L. Nguyen, H.-V. Nguyen, A. Otero-de-la-Roza, L. Paulatto, S. Poncé, D. Rocca, R. Sabatini, B. Santra, M. Schlipf, A. P. Seitsonen, A. Smogunov, I. Timrov, T. Thonhauser, P. Umari, N. Vast, X. Wu, S. Baroni, Advanced capabilities for materials modelling with Quantum ESPRESSO. *J. Phys. Condens. Matter* **29**, 465901 (2017).

141. J. P. Perdew, K. Burke, M. Ernzerhof, Generalized gradient approximation made simple. *Phys. Rev. Lett.* **77**, 3865–3868 (1996).

142. G. Prandini, A. Marrazzo, I. E. Castelli, N. Mounet, N. Marzari, Precision and efficiency in solid-state pseudopotential calculations. *npj Comput. Mater.* **4**, 72 (2018).
