# Supplementary material for: Stable electron-irradiated [1-13C]alanine radicals for metabolic imaging with dynamic nuclear polarization
Source: Sci Adv. 2025 Nov 21;11(47):eadz4334. doi: 10.1126/sciadv.adz4334 (PMC12637281; doi:10.1126/sciadv.adz4334)
Supplement: Supplementary file 2 — Data S1 and S2 [file sciadv.adz4334_data_s1_and_s2.zip › adz4334_data_s1.zip!/AlanineXRD-ScienceAdvances/glycerol_alanine_powder_and_alanine_in_glycerol.pdf]

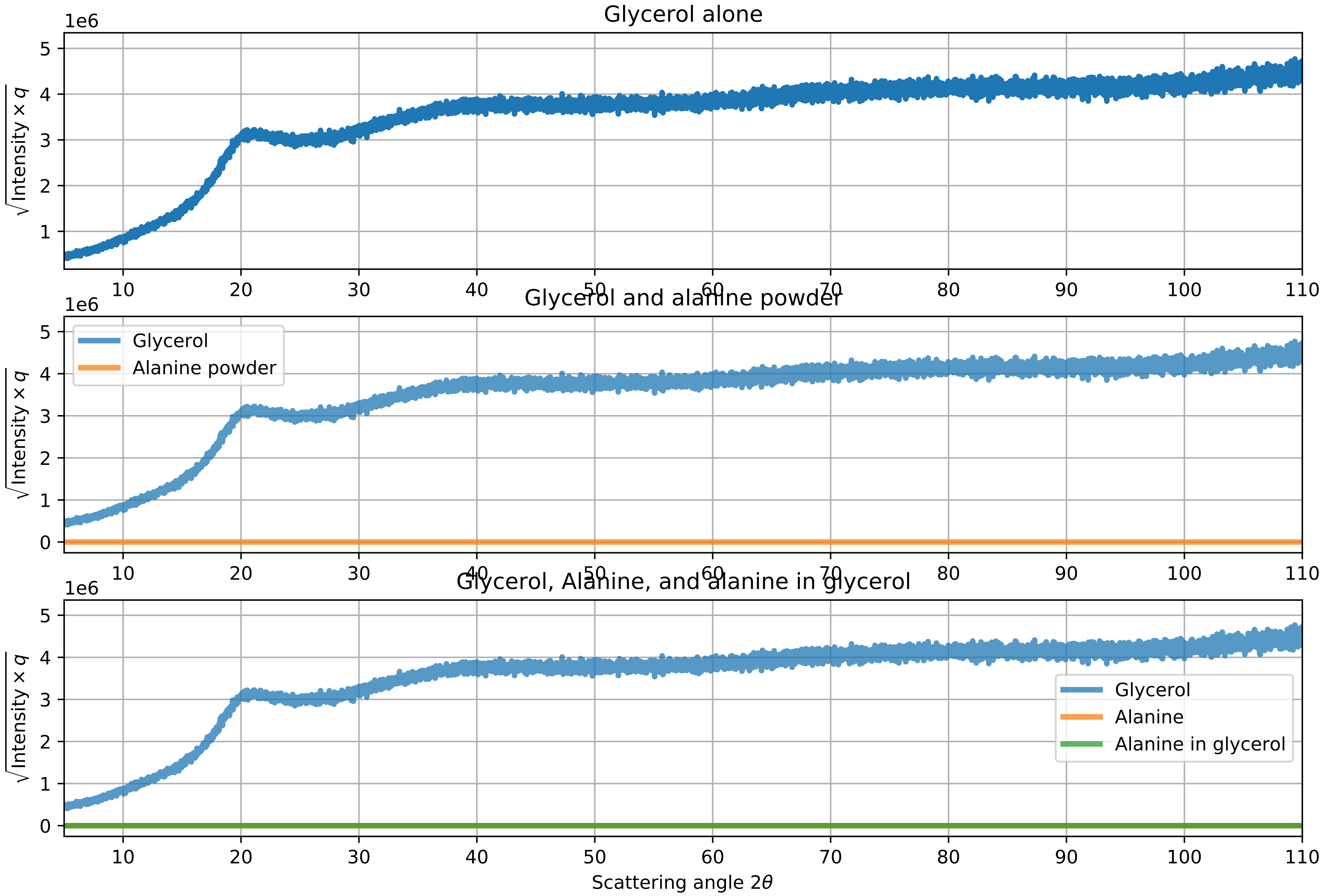

Glycerol alone
1e6
5
4
3
2
1
√Intensity × q
10
20
30
40
50
60
70
80
90
100
110
Glycerol and alanine powder
Glycerol
Alanine powder
0
Glycerol, Alanine, and alanine in glycerol
Glycerol
Alanine
Alanine in glycerol
Scattering angle 2θ
