# Supplementary material for: Stable electron-irradiated [1-13C]alanine radicals for metabolic imaging with dynamic nuclear polarization
Source: Sci Adv. 2025 Nov 21;11(47):eadz4334. doi: 10.1126/sciadv.adz4334 (PMC12637281; doi:10.1126/sciadv.adz4334)
Supplement: Supplementary file 2 — Data S1 and S2 [file sciadv.adz4334_data_s1_and_s2.zip › adz4334_data_s1.zip!/AlanineXRD-ScienceAdvances/HarmonicTextureAnalysis.pdf]

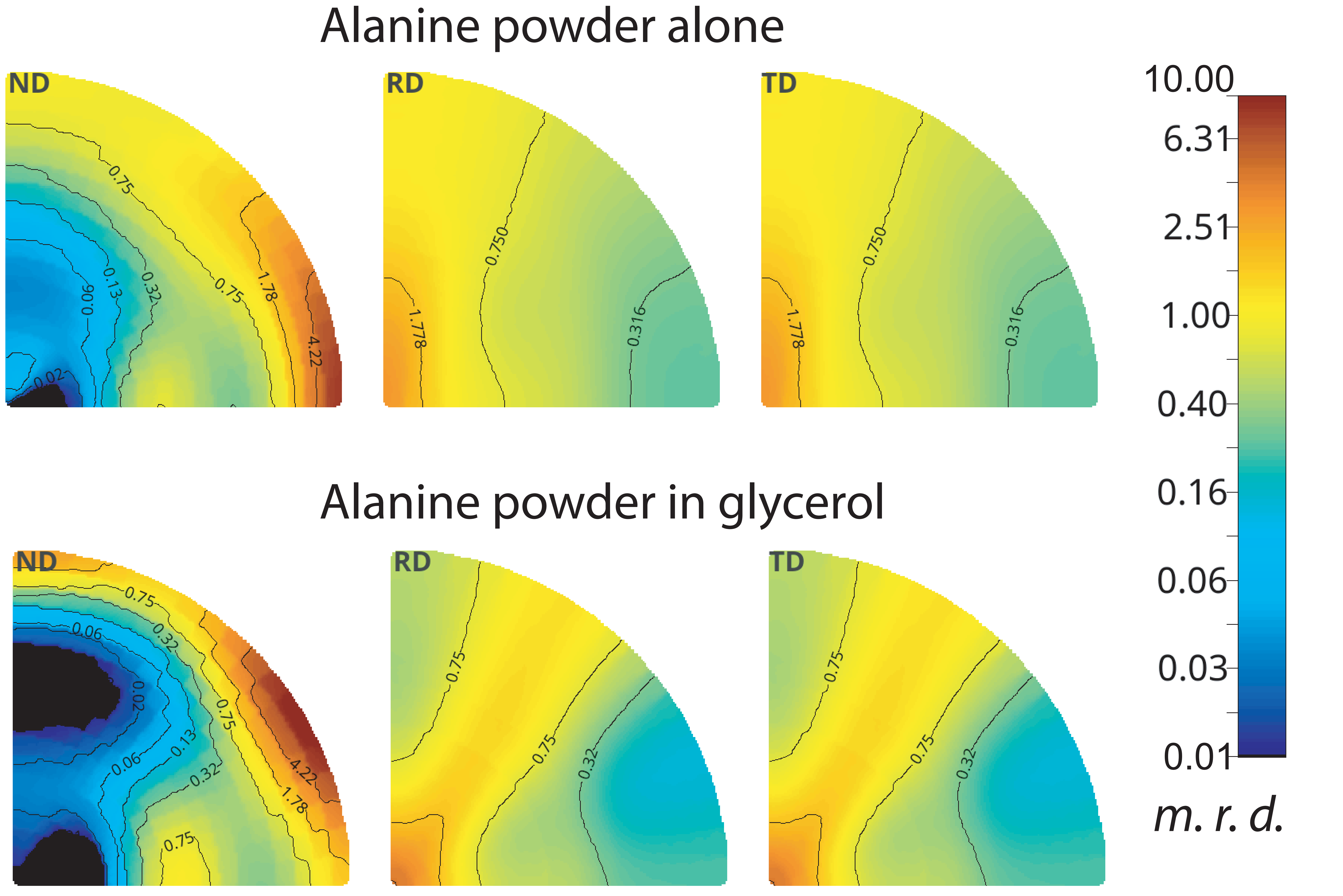
Alanine powder alone
ND
0.75
0.06
0.13
0.32
0.75
1.78
4.22
0.02
RD
0.750
1.778
0.316
TD
0.750
1.778
0.316
Alanine powder in glycerol
ND
0.75
0.06
0.32
0.02
0.75
0.13
0.06
0.32
4.22
1.78
0.75
RD
0.75
0.75
0.32
TD
0.75
0.75
0.32
10.00
6.31
2.51
1.00
0.40
0.16
0.06
0.03
0.01
m. r. d.
