# Supplementary material for: Stable electron-irradiated [1-13C]alanine radicals for metabolic imaging with dynamic nuclear polarization
Source: Sci Adv. 2025 Nov 21;11(47):eadz4334. doi: 10.1126/sciadv.adz4334 (PMC12637281; doi:10.1126/sciadv.adz4334)
Supplement: Supplementary file 2 — Data S1 and S2 [file sciadv.adz4334_data_s1_and_s2.zip › adz4334_data_s1.zip!/AlanineXRD-ScienceAdvances/Model_fit_comparisons.pdf]

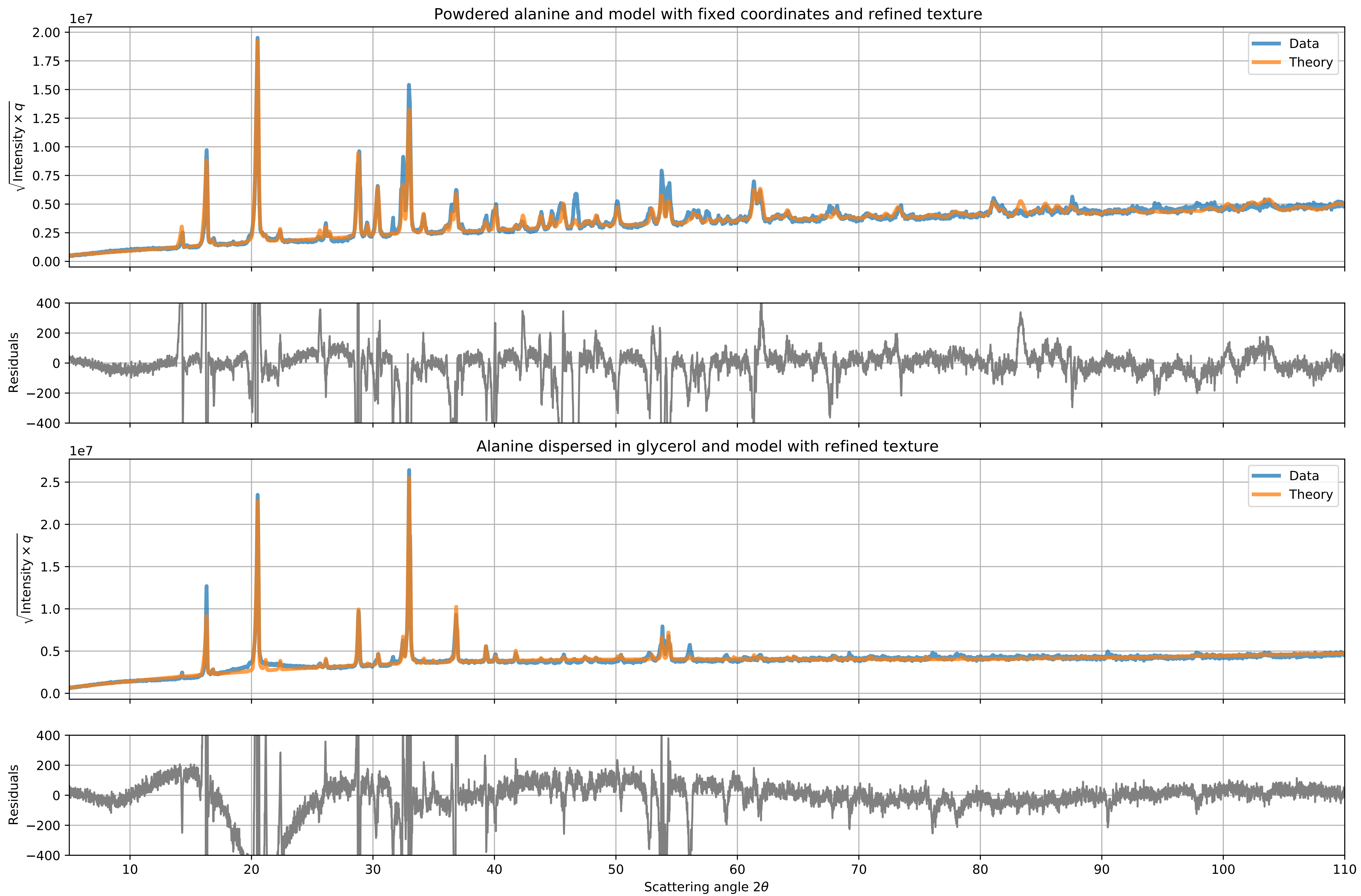

Powdered alanine and model with fixed coordinates and refined texture
1e7
2.00
1.75
1.50
1.25
1.00
0.75
0.50
0.25
0.00
$\sqrt{\text{Intensity}} \times q$
Data
Theory
400
200
0
−200
−400
Residuals
Alanine dispersed in glycerol and model with refined texture
1e7
2.5
2.0
1.5
1.0
0.5
0.0
$\sqrt{\text{Intensity}} \times q$
Data
Theory
400
200
0
−200
−400
Residuals
10
20
30
40
50
60
70
80
90
100
110
Scattering angle 2θ
