# Supplementary material for: Stable electron-irradiated [1-13C]alanine radicals for metabolic imaging with dynamic nuclear polarization
Source: Sci Adv. 2025 Nov 21;11(47):eadz4334. doi: 10.1126/sciadv.adz4334 (PMC12637281; doi:10.1126/sciadv.adz4334)
Supplement: Supplementary file 2 — Data S1 and S2 [file sciadv.adz4334_data_s1_and_s2.zip › adz4334_data_s1.zip!/AlanineXRD-ScienceAdvances/Model_fit_comparisons_Shrunk.pdf]

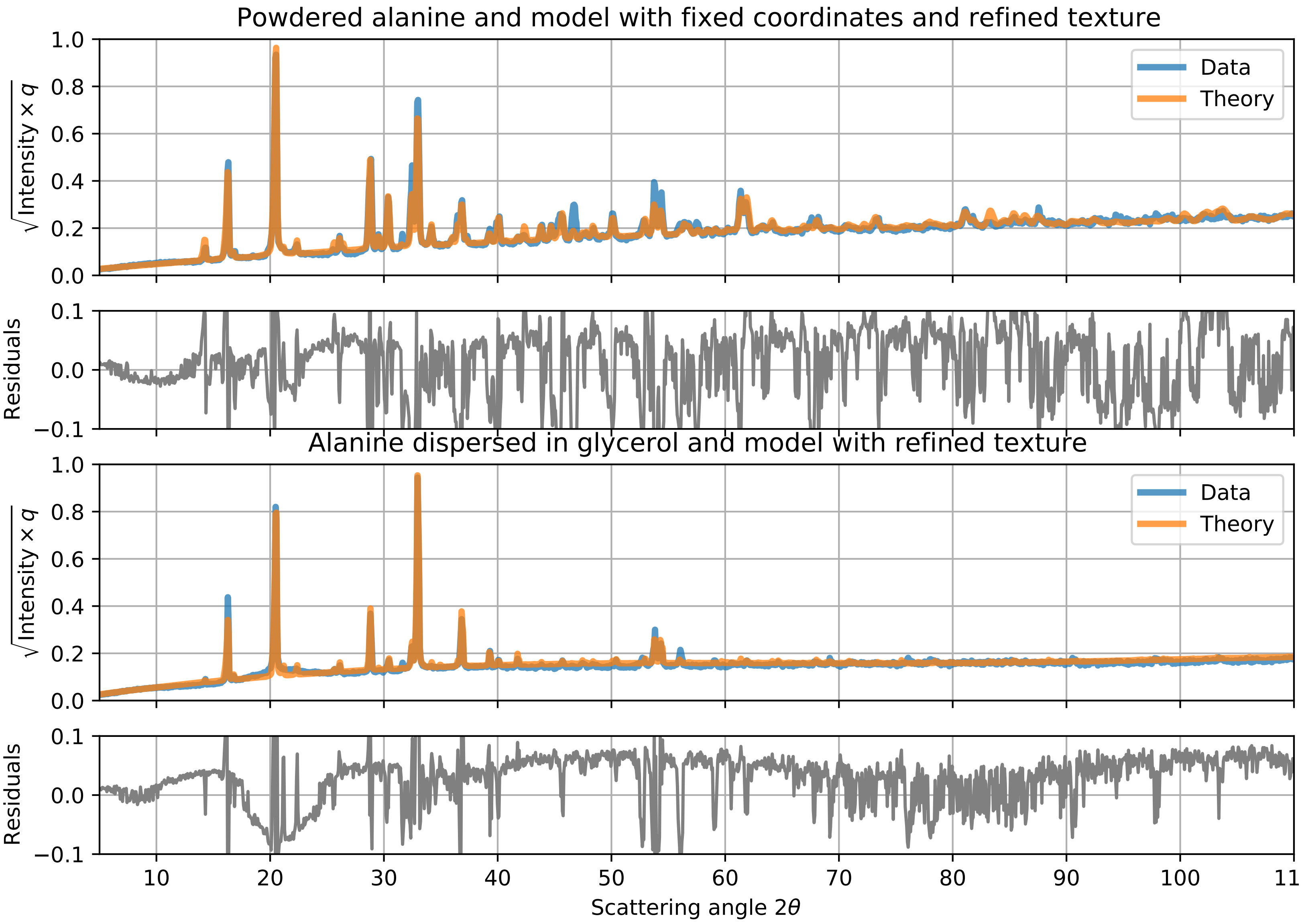

Powdered alanine and model with fixed coordinates and refined texture
Data
Theory
$\sqrt{\text{Intensity}} \times q$
Residuals
Alanine dispersed in glycerol and model with refined texture
Data
Theory
$\sqrt{\text{Intensity}} \times q$
Residuals
Scattering angle 2θ
